# Automated Evaluation of Reflection and Feedback Quality in Workplace-Based Assessments by Using Natural Language Processing: Cross-Sectional Competency-Based Medical Education Study

**DOI:** 10.2196/81718

**Published:** 2025-10-22

**Authors:** Jeng-Wen Chen, Hai-Lun Tu, Chun-Hsiang Chang, Wei-Chung Hsu, Pa-Chun Wang, Chun-Hou Liao, Mingchih Chen

**Affiliations:** 1 Department of Otolaryngology–Head and Neck Surgery Cardinal Tien Hospital Fu Jen Catholic University New Taipei City Taiwan; 2 Department of Otolaryngology–Head and Neck Surgery National Taiwan University Hospital and Children’s Hospital Taipei Taiwan; 3 Department of Hospital Management Graduate Institute of Business Administration Fu Jen Catholic University New Taipei City Taiwan; 4 Department of Education and Research Cardinal Tien Junior College of Healthcare and Management New Taipei City Taiwan; 5 Department of Library and Information Science Fu-Jen Catholic University New Taipei City Taiwan; 6 Cathay General Hospital Department of Otolaryngology Taipei Taiwan; 7 School of Medicine Fu-Jen Catholic University New Taipei City Taiwan; 8 Department of Medical Research China Medical University Hospital China Medical University Taichung Taiwan; 9 Department of Surgery, Division of Urology Cardinal Tien Hospital and School of Medicine Fu Jen Catholic University New Taipei City Taiwan; 10 Artificial Intelligence Development Center Fu Jen Catholic University New Taipei City Taiwan

**Keywords:** competency-based medical education, entrustable professional activities, otolaryngology, residency, workplace-based assessment, reflection, feedback, Emyway platform

## Abstract

**Background:**

Competency-based medical education relies heavily on high-quality narrative reflections and feedback within workplace-based assessments. However, evaluating these narratives at scale remains a significant challenge.

**Objective:**

This study aims to develop and apply natural language processing (NLP) models to evaluate the quality of resident reflections and faculty feedback documented in Entrustable Professional Activities (EPAs) on Taiwan’s nationwide Emyway platform for otolaryngology residency training.

**Methods:**

This 4-year cross-sectional study analyzes 300 randomly sampled EPA assessments from 2021 to 2025, covering a pilot year and 3 full implementation years. Two medical education experts independently rated the narratives based on relevance, specificity, and the presence of reflective or improvement-focused language. Narratives were categorized into 4 quality levels—effective, moderate, ineffective, or irrelevant—and then dichotomized into high quality and low quality. We compared the performance of logistic regression, support vector machine, and bidirectional encoder representations from transformers (BERT) models in classifying narrative quality. The best performing model was then applied to track quality trends over time.

**Results:**

The BERT model, a multilingual pretrained language model, outperformed other approaches, achieving 85% and 92% accuracy in binary classification for resident reflections and faculty feedback, respectively. The accuracy for the 4-level classification was 67% for both. Longitudinal analysis revealed significant increases in high-quality reflections (from 70.3% to 99.5%) and feedback (from 50.6% to 88.9%) over the study period.

**Conclusions:**

BERT-based NLP demonstrated moderate-to-high accuracy in evaluating the narrative quality in EPA assessments, especially in the binary classification. While not a replacement for expert review, NLP models offer a valuable tool for monitoring narrative trends and enhancing formative feedback in competency-based medical education.

## Introduction

Medical education has undergone a fundamental transformation, with competency-based medical education (CBME) emerging as a central paradigm [1]. In contrast to traditional time-based models that focus on the completion of predetermined curricula over fixed durations, CBME emphasizes the direct assessment of learner’s abilities to perform core professional activities safely and effectively in authentic clinical environments [2,3]. This outcomes-oriented approach aims to ensure that physicians are not only knowledgeable but also clinically competent, adaptable, and equipped to address the evolving complexities of patient care [4-6].

The field of otorhinolaryngology–head and neck surgery underscores the urgency of this educational shift, given its demand for proficiency in complex surgical procedures and nuanced clinical decision-making [7,8]. In response, the Taiwan Society of Otorhinolaryngology–Head and Neck Surgery (TSO-HNS) launched a structured competency framework in 2020, introducing 11 Entrustable Professional Activities (EPAs) as benchmarks for assessing resident performance (TSO-HNS Entrustable Professional Activities Assessment Framework for Resident Physician Training, second edition; see Multimedia Appendix 1). To support the systematic implementation of these EPAs, the Emyway digital platform was adopted in 2021, enabling more structured, transparent, and objective competency evaluations [9]. Central to Emyway is the integration of workplace-based assessment (WBA), which promotes continuous learning through direct observation, self-reflection, formative feedback, and performance appraisal in real-world clinical settings [10,11]. Unlike traditional assessments, WBAs offer dynamic, individualized insights that inform both clinical decision-making and technical skill development [9].

A key challenge in CBME is bridging the gap between assessment and learning. Reflection and feedback play complementary roles in this process. When aligned, feedback shapes the focus of reflection, and reflection deepens engagement with feedback, turning assessments into learning opportunities. However, prior studies show that reflections often remain descriptive, and feedback lacks specificity, limiting their combined educational value [12,13]. Evaluating the quality of both processes is therefore essential to understanding how WBAs contribute to learning. A growing body of evidence underscores the role of high-quality reflections and feedback in reinforcing core competencies and enhancing learning outcomes [14,15]. However, the quality of these narrative components within WBAs—particularly in otolaryngology residency programs and in multilingual training environments—remains insufficiently studied.

A major challenge in the implementation of CBME is managing the substantial volume of narrative data generated through WBAs [11]. On digital platforms such as Emyway, thousands of EPA evaluations are recorded, rendering manual review impractical. Traditional assessment methods that rely on human interpretation are time-consuming, resource-intensive, and susceptible to variability, limiting their ability to yield consistent and meaningful insights from large datasets [16]. Overcoming this challenge requires innovative strategies to ensure that narrative reflections and feedback remain relevant, specific, and actionable—supporting continuous learning and improvement in residency training [17,18].

This study aims to address the challenge of evaluating narrative data in CBME by applying natural language processing (NLP) to systematically assess the quality of resident reflections and faculty feedback recorded within the Emyway platform. To capture these distinct but interrelated processes at scale, we applied NLP models to evaluate reflection and feedback separately, allowing for a clearer analysis of their respective contributions to CBME. We hypothesize that NLP can provide an objective, consistent, and scalable method for evaluating the effectiveness of narrative assessments, offering valuable insights into how feedback contributes to residents’ competency development [16,19]. By leveraging NLP, this study seeks to improve the relevance, specificity, and actionability of reflections and feedback, thereby enhancing the guidance residents receive for their professional growth [19-22]. Resident reflections and faculty feedback are distinct constructs: reflections involve personal self-assessment, while feedback represents external evaluation from faculty. Although different, they occur simultaneously within the same WBA encounter. This study therefore examines both while ensuring that the NLP models and evaluation rubrics for reflections and feedback were developed and analyzed independently. Ultimately, this approach aims to bridge the gap between assessment and learning, strengthen CBME implementation, and support the development of a more robust otolaryngology residency training system.

## Methods

### Ethical Considerations

This study adheres to established ethical standards for medical education research. Informed consent was obtained actively. Participants were required to read the “Training-Related Data Collection and Privacy Information” and click an “I agree” button before accessing the Emyway platform. The participants did not receive any compensation for their participation. The system includes built-in data protection mechanisms to prevent confidential information from being displayed. All data were deidentified prior to analysis, with personal identifiers removed, and access was restricted to the research team through secure, password-protected servers. The study protocol was reviewed and approved by the institutional review board of Cardinal Tien Hospital (CTH-112-2-1-002).

### Study Design and Setting

This cross-sectional study examines the quality of resident reflections and faculty feedback recorded in the Emyway platform of TSO-HNS between 2021 and 2025. Emyway is a nationwide digital platform designed to support CBME by systematically collecting workplace-based EPA assessments from otolaryngology residency programs across Taiwan [9]. Basic clinical information, encounter descriptions, resident reflections, and subsequent faculty feedback and ad hoc entrustment decisions were collected within a single standardized electronic form on the Emyway platform [9]. The primary objective of this study was to evaluate the narrative quality of resident reflections and faculty feedback by using NLP algorithms, with the goal of improving assessment reliability and enhancing the educational value of feedback in clinical training.

### Data Collection and Sample Selection

We selected 300 EPA assessment entries from the Emyway national database, covering the period from 2021 to 2025. Each entry included structured fields such as the EPA title, clinical diagnosis, and narrative components authored by both residents and faculty [9]. To ensure diversity and representativeness, we employed stratified random sampling across training years, resident levels, and EPA categories. To reduce potential bias related to temporal improvements in narrative quality, we used cross-validation and ensured a balanced distribution of entries across earlier and later phases of implementation. Only complete assessments containing both resident reflections and faculty feedback were included in the final analysis.

### Narrative Quality Assessment

Two medical education experts—one a physician-educator specializing in otolaryngology residency training and the other a senior faculty developer with expertise in educational measurement and feedback assessment—independently evaluated the quality of resident reflections and faculty feedback by using a structured rubric based on the core principles of CBME. Narratives were evaluated using established rubrics developed by Solano et al [17] and Ötleş et al [18], which have been previously validated in surgical residency programs and were adopted in our study without modification to ensure consistency with the existing literature. The rubric assesses 3 key dimensions: relevance, specificity, and either reflection content (for resident narratives) or actionability (for faculty feedback). Relevance evaluates the alignment of the narrative with the EPA and the clinical context. Specificity measures the clarity and detail with which strengths, weaknesses, or areas for improvement were identified. Reflection content assesses the presence of self-directed learning goals in resident narratives, while actionability examines whether faculty feedback provided clear, constructive guidance to support resident development. The analysis of interrater reliability showed a fair to moderate agreement in the 4-level classification and a substantial to almost perfect agreement in the 2-level classification (Table S1 in Multimedia Appendix 2). In cases where the 2 expert raters had discrepancies in their ratings, a third reviewer (the corresponding author) adjudicated and made the final decision to ensure consistency and accuracy in the gold standard dataset.

Based on the evaluation criteria, narratives were categorized into 4 quality levels (Table 1): effective, moderate, ineffective, and irrelevant. Effective narratives were both relevant and specific; resident reflections demonstrated meaningful insight, and faculty feedback included actionable guidance. Moderate narratives maintained relevance but demonstrated only one additional element—either specificity or reflection content for residents or actionability for faculty. Ineffective narratives were superficially related to the EPA but lacked depth, with vague language and an absence of both specificity and meaningful reflection or guidance. Irrelevant narratives were off-topic, superficial, or disconnected from the clinical context. In this study, “high quality” refers to the combined category in the 2-level classification (encompassing both effective and moderate narratives) and “low quality” refers to ineffective and irrelevant narratives, whereas “effective” denotes the highest category within the 4-level classification.

**Table 1:** Classification of the quality levels in residents’ reflections and faculty feedback.

Characteristics according to the 4-level classification^a^	Quality of narrative content				
	Effective^b^	Moderate^b^	Moderate^b^	Ineffective^c^	Irrelevant^c^
Relevance	Yes	Yes	Yes	Yes	No
Specificity	Yes	Yes	No	No	N/A^d^
Reflection content in residents’ reflections	Yes	No	Yes	No	N/A
Action plan in faculty feedback	Yes	No	Yes	No	N/A

^a^In the 4-level classification, the categories are effective (highest quality), moderate, ineffective, and irrelevant.

^b^The combined group of effective and moderate narratives was classified as high quality per the 2-level classification.

^c^Ineffective and irrelevant narratives were classified as low quality per the 2-level classification.

^d^N/A: not applicable.

### NLP Framework

To enhance the scalability and objectivity of narrative assessment, NLP techniques were applied to analyze resident reflections and faculty feedback. Two independent NLP models were developed and trained separately for reflections and feedback, ensuring that the classification processes remained independent while allowing both dimensions to be examined within the same WBA encounter. Three supervised machine learning models were implemented for classification: logistic regression (LR) [23], support vector machine (SVM) [24], and bidirectional encoder representations from transformers (BERT) [25], which is a state-of-the-art deep learning model for natural language understanding.

### Data Preprocessing and Feature Extraction

For traditional machine learning models such as LR and SVM, text preprocessing included tokenization using CKIPtagger for Chinese language segmentation, followed by transformation into term frequency–inverse document frequency feature vectors. In contrast, the BERT model processed raw text inputs directly, structured as a combination of context, EPA title, diagnosis, and either reflection or feedback. This approach leveraged BERT’s ability to generate contextualized embeddings without requiring additional preprocessing.

### Model Training and Evaluation

To evaluate model performance, the dataset was randomly divided into a training set (80%) and a validation set (20%). Both fine-grained (4-level) and binary (2-level) classification models were developed to assess the impact of classification granularity. LR and SVM models were implemented using the *scikit-learn* library, while the BERT model was fine-tuned using the *simpletransformers* library with the pretrained BERT-base-multilingual-uncased model. BERT was trained for 10 epochs with a learning rate of 2e-5. The code used for training all the models is provided in Multimedia Appendix 3.

### Performance Metrics and Narrative Quality Trend Analysis

We evaluated model performance by using standard metrics, including accuracy, precision, recall, and *F*_1_-score. We generated confusion matrices to visualize classification outcomes and identify patterns of misclassification. The analysis aimed to assess the accuracy of distinguishing high-quality and low-quality reflections and feedback, compare the performance across different machine learning models, and explore longitudinal trends in the narrative quality by using the best performing model throughout the study period from 2021 to 2025.

## Results

### Overall Model Performance

Across the study period, the majority of EPA assessments were complete, containing both resident reflections and faculty feedback. Specifically, 90.1% (1422/1580) were complete in the pilot year (2021-2022), 95.1% (9939/10,447) in 2022-2023, 96.7% (10,601/10,966) in 2023-2024, and 97.1% (12,139/12,497) in 2024-2025. In total, 34,101 out of 35,490 assessments (96.1%) were complete and included in the final analysis. Table 2 presents the expert-assessed quality distribution of 300 randomly selected EPA entries, comprising resident reflections and faculty feedback, used for developing and validating the NLP models.

Table 3 summarizes the prediction outcomes from the 3 models evaluated in the study. The NLP-based classification models demonstrated substantial accuracy in assessing the quality of both resident reflections and faculty feedback, with the BERT model consistently outperforming the LR and SVM models. Specifically, for resident reflections, the BERT model achieved an accuracy of 85% for the 2-level classification and 67% for the more granular 4-level classification. Performance was even stronger for faculty feedback evaluation, where the BERT model attained an accuracy of 92% in the 2-level classification and maintained a 67% accuracy for the 4-level classification. Additionally, precision, recall, and *F*_1_-scores showed consistent patterns across these evaluations, supporting the robustness and reliability of the BERT model.

**Table 2:** Distribution of expert-assessed quality of 300 randomly selected Entrustable Professional Activity entries (resident reflections and faculty feedback) for natural language processing model development and validation.

Classification/quality rating			Resident reflections (n=300), n (%)		Faculty feedback (n=300), n (%)
**4-level classification**					
	Effective	134 (44.7)		168 (56)	
	Moderate	86 (28.7)		28 (9.3)	
	Ineffective	49 (16.3)		24 (8)	
	Irrelevant	31 (10.3)		80 (26.7)	
**2-level classification**					
	High-quality	220 (73.3)		196 (65.3)	
	Low-quality	80 (26.7)		104 (34.7)	

**Table 3:** Prediction results of the residents’ reflections and faculty feedback by the 3 models in the study.

Narrative content, model		4-level classification					2-level classification			
		Accuracy (%)	Precision (%)	Recall (%)	*F*_1_-score	Accuracy (%)		Precision (%)	Recall (%)	*F*_1_-score
**Resident reflections**										
	LR^a^	63	66	63	64	80		83	80	81
	SVM^b^	60	63	60	60	85		85	85	85
	BERT^c^	67	67	67	65	85		85	85	85
**Faculty feedback**										
	LR	63	55	63	59	78		78	78	78
	SVM	63	54	63	54	78		81	78	76
	BERT	67	65	67	64	92		92	92	92

^a^LR: logistic regression.

^b^SVM: support vector machine.

^c^BERT: bidirectional encoder representations from transformers.

### Confusion Matrix Analysis

**Figure 1:**
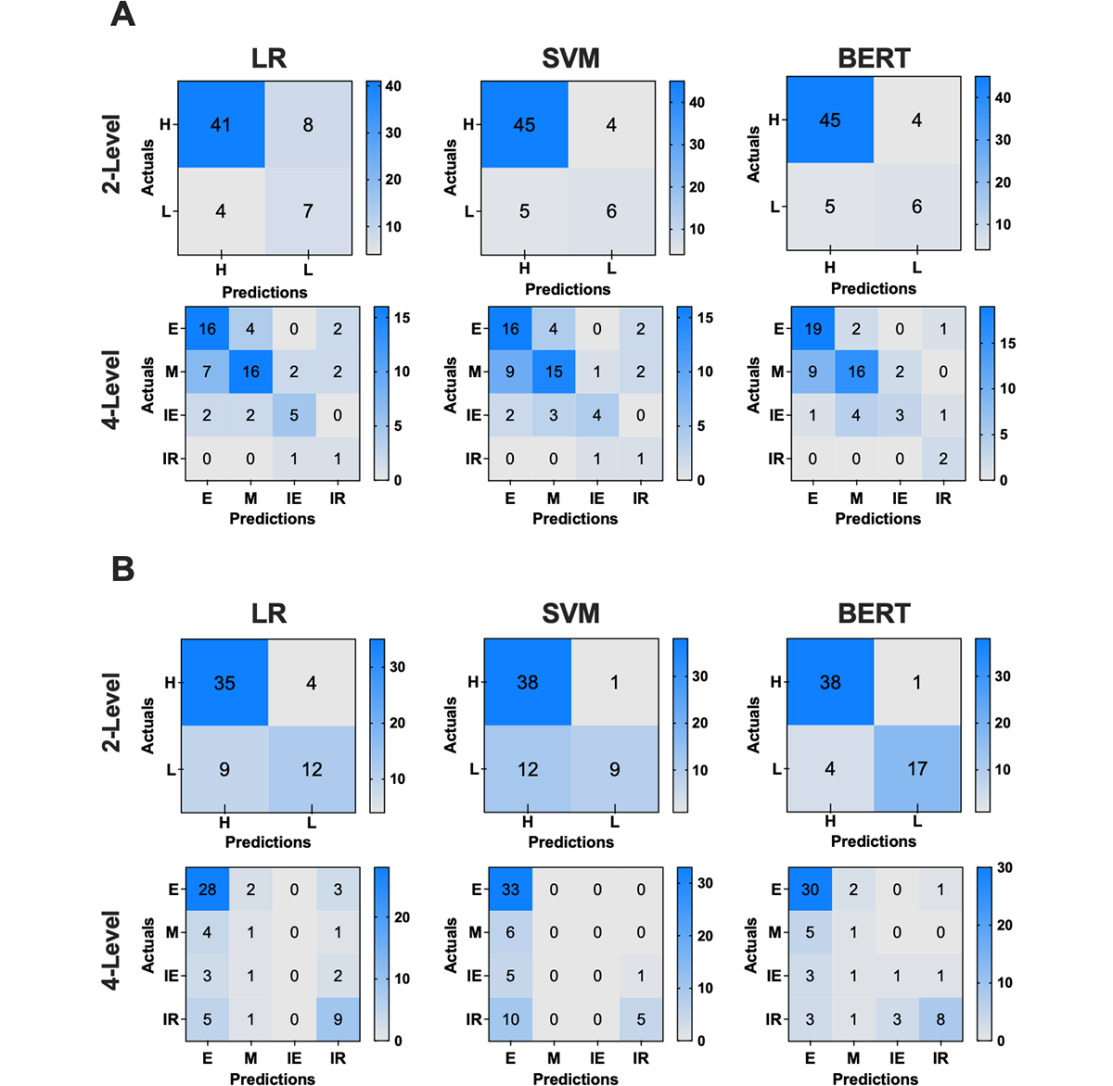
Confusion matrices illustrating the classification performance of 3 natural language processing models—LR, SVM, and BERT—in evaluating the quality of resident reflections (A) and faculty feedback (B). The x-axis represents predicted categories, and the y-axis represents actual expert ratings. For the 2-level classification, narratives were categorized as high quality (H) or low quality (L). For the 4-level classification, the categories are effective (E), moderate (M), ineffective (IE), and irrelevant (IR). Numbers within each cell indicate the count of narratives, while shading intensity reflects frequency (darker=higher count). Compared with LR and SVM, BERT demonstrated fewer misclassifications and stronger performance in distinguishing between adjacent categories, particularly for faculty feedback. BERT: bidirectional encoder representations from transformers; LR: logistic regression; SVM: support vector machine.

To further assess model performance, confusion matrices were generated (Figure 1). The BERT model exhibited fewer misclassifications than LR and SVM, particularly in distinguishing between effective and moderate narratives. In contrast, LR and SVM frequently misclassified effective narratives as moderate or irrelevant, reflecting their limitations in detecting subtle contextual cues. Notably, BERT’s superior classification capability was most evident in faculty feedback, where its accuracy surpassed 90%, demonstrating its potential to improve automated assessment reliability in competency-based education frameworks. To illustrate the model’s interpretability and limitations, Table S2 in Multimedia Appendix 4 presents anonymized examples of correctly classified and misclassified narratives.

### Two-Level and Four-Level Quality Classification Outcomes in the Emyway Platform

**Figure 2:**
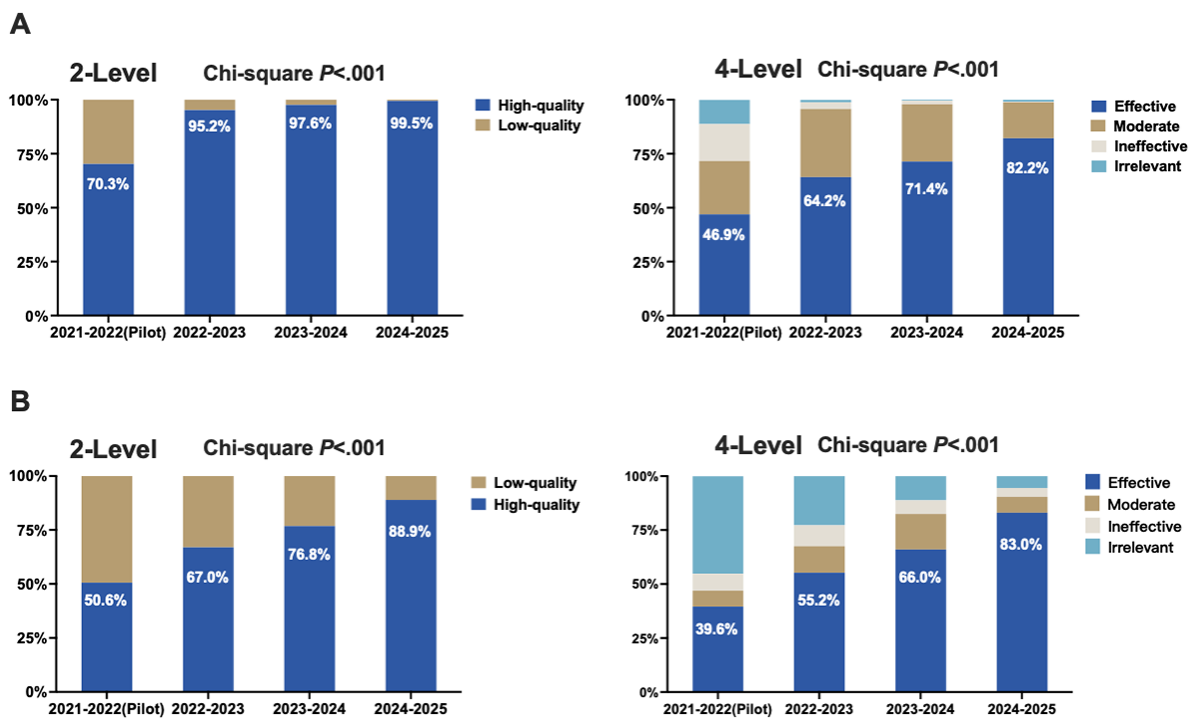
Longitudinal trends in the quality of narrative assessments from 2021 to 2025, as classified by the bidirectional encoder representations from transformers model. Panel A displays resident reflections; panel B displays faculty feedback. In each panel, the left graph shows the 2-level classification (high quality vs low quality), and the right graph shows the 4-level classification (effective, moderate, ineffective, irrelevant). The x-axis represents academic years, with 2021-2022 as the pilot year, followed by 3 full implementation years. The y-axis indicates the percentage distribution of the narratives. Over time, both resident reflections and faculty feedback showed a significant increase in the proportion of high-quality and effective narratives.

Figure 2 illustrates the longitudinal trends in the narrative quality of resident reflections and faculty feedback, as classified by the BERT model using both 2-level and 4-level rating algorithms, across 4 academic years: the pilot year (2021-2022) through 2024-2025. Detailed distributions of frequencies and percentages are presented in Table S3 of Multimedia Appendix 5.

In the 2-level classification, the proportion of high-quality resident reflections increased from 70.3% to 99.5%, while high-quality faculty feedback increased from 50.6% to 88.9% over the study period. Chi-square analyses confirmed that these improvements were statistically significant (*P*<.001 for both groups), reflecting meaningful enhancement in the quality of narrative documentation. Similarly, in the 4-level classification, the proportion of “effective” resident reflections increased from 46.9% to 82.2%, and “effective” faculty feedback increased from 39.6% to 83%. These gains were also statistically significant (*P*<.001), suggesting a sustained and substantive improvement in narrative quality over time, likely associated with the ongoing implementation of structured EPA frameworks and digital feedback systems.

## Discussion

### Principal Findings

This study demonstrates the utility of NLP, specifically the BERT algorithm, in evaluating the narrative quality within WBAs in otolaryngology residency training. The BERT model achieved high accuracy in the binary classification—85% for resident reflections and 92% for faculty feedback—supporting its potential as a scalable, objective adjunct to manual evaluation. Notably, narrative quality improved significantly over the study period, with high-quality reflections increasing from 70.3% to 99.5% and high-quality faculty feedback from 50.6% to 88.9%. These findings highlight the potential of NLP to enhance quality assurance and longitudinal monitoring in CBME.

Compared to traditional manual qualitative analysis, NLP offers unique advantages [26]. Although human raters can capture contextual nuance and interpret implicit meaning, their assessments are time-intensive and subject to interrater variability. In contrast, NLP enables consistent, rapid, and scalable evaluation across large datasets [27,28]. Prior research by Akbasli et al [29] has demonstrated the feasibility of applying fine-tuned language models to non-English and multilingual medical texts. Our findings further support this approach, showing that integrating structured contextual inputs such as EPA titles, clinical diagnoses, and narrative components substantially enhance model accuracy. With adequate structured contextual inputs, BERT approximates human interpretive depth while retaining the efficiency and objectivity of automation.

This approach should also be interpreted through the lens of the educational assessment theory. Beyond its statistical performance, the application of NLP algorithms in this study aligns closely with established educational assessment theories and feedback quality frameworks. The structured rubric used to generate the gold standard—encompassing relevance, specificity, and either having reflection content or actionability—reflects the core principles found in frameworks such as the Feedback Quality Instrument [30-32] and the R2C2 model (relationship building, exploring reactions, exploring content, coaching for change) [14,33,34]. These frameworks emphasize that effective feedback and reflection must be contextually relevant, sufficiently specific, and actionable to promote self-regulated learning and professional growth. By incorporating these dimensions into the training data, BERT’s decision-making process operationalizes these theoretical constructs, mapping narrative text to empirically validated quality indicators. In this way, the model does not merely classify text based on linguistic patterns but also embeds the pedagogical priorities of CBME and EPA assessment. This alignment ensures that automated scoring supports the same developmental goals as expert human raters, enabling the model to serve as a theoretically grounded, scalable complement to manual evaluation.

However, it is important to clarify that the R2C2 model is a coaching framework designed to structure feedback conversations rather than an evaluation rubric for written comments. In this study, R2C2 was referenced as a conceptual lens to underscore the coaching potential embedded in high-quality narrative feedback and not as a scoring tool. Recent literature has emphasized its role in facilitating meaningful faculty–learner interactions in WBAs [35,36]. Our findings on the quality of written reflections and feedback should therefore be viewed as complementary to, rather than substitutive of, coaching frameworks such as R2C2, providing a stronger foundation for effective feedback dialogue.

In addition to methodological contributions, our findings suggest practical applications for residency programs. NLP outputs could be integrated into dashboards that track reflection and feedback quality over time, enabling program directors to identify gaps and design targeted faculty development workshops. At the same time, residents could receive timely, formative, reflective prompts into the quality of their reflections. By embedding these tools into CBME frameworks, narrative data can serve not only as an assessment record but also as a resource to strengthen feedback culture and support continuous coaching.

### Comparison With Previous Studies

The superior performance of BERT relative to traditional machine learning models such as LR and SVM is a key contribution of this study. For instance, previous work by Ötleş et al [18] reported a mean accuracy of 0.64 by using SVM for the 4-level classification of surgical feedback, which improved to 0.83 when simplified to binary classification. Similarly, Solano et al [17] achieved an overall accuracy of 0.83 by using NLP but noted limitations in sensitivity (0.37), suggesting challenges in detecting lower quality feedback. In contrast, our BERT-based model achieved 85% accuracy for resident reflections and 92% for faculty feedback in binary classification, with balanced precision and recall scores. These results highlight BERT’s superior ability to contextualize text and detect nuanced linguistic patterns. Unlike traditional models, BERT effectively interprets the complex, often implicit nature of reflective narratives, validating its use in educational quality assessment within clinical training contexts [37]. This capacity is particularly valuable, as reflective writing in medical education is typically layered, context-sensitive, and difficult to assess using rule-based or shallow models [38,39].

Although the 4-level classification achieved only moderate accuracy, its outputs can still inform educational practice. Even without perfect distinction between adjacent categories, the model can highlight patterns of lower quality narratives that may warrant attention. For instance, faculty development dashboards could flag programs or individuals generating a higher proportion of ineffective or moderate entries, prompting targeted coaching or workshops. These applications position the model as a supportive tool for monitoring and guiding feedback culture, complementing human judgment rather than replacing it.

Unlike prior studies that emphasized cross-sectional performance [17,18], this research provides longitudinal evidence of NLP’s ability to track and support improvements in feedback quality over time. Consistent with earlier findings, the model maintained high specificity, particularly in identifying low-quality narratives—a valuable feature for faculty development and system-level monitoring. Although the 4-level classification performance remained moderate (67% accuracy), this aligns with known challenges in distinguishing subtle qualitative gradations and highlights areas for future enhancement.

The sustained improvement in the reflection quality across the study period underscores the value of structured WBA systems such as those implemented through the Emyway platform. These systems provide clear expectations and guidance, promoting deeper engagement, self-awareness, and professional development [40]. This observation aligns with literature indicating that structured reflection fosters clinical reasoning, self-regulated learning, and long-term growth [41-44].

Faculty feedback quality also improved substantially, increasing in specificity, relevance, and actionability. While still trailing resident reflections in overall quality, the upward trajectory from 50.6% to 88.9% suggests growing familiarity with EPA-based frameworks and greater faculty engagement. These findings reinforce the importance of structured systems in supporting effective feedback practices. NLP tools, in this context, can function as educational dashboards—tracking feedback quality across programs and timeframes, flagging low-quality entries, and informing faculty development and institutional policy.

It is important to note that reflection quality and feedback quality were not conflated in this study; rather, they were modeled separately using independent rubrics and NLP training processes. Presenting them together highlights how these complementary elements of the same assessment encounter can be studied in parallel to inform faculty development and resident learning.

We selected BERT over commercial large language models such as ChatGPT for both practical and performance-based reasons. As an open-source model, BERT is accessible to academic institutions without licensing constraints, facilitating integration into resource-limited settings. Moreover, internal comparisons indicated that ChatGPT, while powerful, lacked discriminative precision in this context and frequently defaulted to mid-range classifications (Multimedia Appendix 6). In contrast, BERT demonstrated greater reliability and accuracy, particularly when provided with structured contextual information.

### Generalizability

Although our findings highlight the utility of BERT-based NLP within Taiwan’s structured otolaryngology training system, their generalizability to other specialties, languages, and international contexts remains uncertain. Narrative style, cultural norms, and feedback practices vary widely across training environments, potentially affecting model performance. To ensure validity in non-Chinese language settings, rubric recalibration would be needed to align evaluation criteria with local educational practices and expectations. Furthermore, although multilingual pretrained models such as BERT provide a strong foundation, language-specific fine-tuning with locally generated narrative data would be required to capture semantic nuances and ensure accurate classification. These adaptations highlight the importance of international replication and validation, which will be essential to confirm generalizability and extend the impact of NLP-assisted evaluation across medical specialties and cultural contexts.

The use of open-source NLP tools such as BERT also carries important ethical and practical implications. Although these models provide scalability, accessibility, and adaptability for educational use, they raise concerns about confidentiality, data security, and potential bias. To ensure responsible application, future implementation should include secure data management, careful local fine-tuning, and ongoing evaluation of fairness so that such tools enhance rather than compromise educational integrity.

### Limitations

Despite encouraging results in binary classification, several limitations should be noted. First, the model’s 67% accuracy in the 4-level classification reflects the inherent difficulty of distinguishing subtle qualitative differences in narrative assessments. Overlap in language used across adjacent categories—such as moderate and ineffective—poses challenges for both human raters and machine learning models. This limitation is common in educational NLP research and underscores the need for larger, more diverse training datasets, domain-specific model fine-tuning, and potentially incorporating contextual metadata (eg, resident level or case type). Although model performance stabilized during cross-validation, suggesting that the sample was adequate for the study objectives, larger datasets could further strengthen robustness. Moreover, the limited sample size may have contributed to weaker performance in the 4-level classification. Future strategies to address this limitation include expanding the dataset as the Emyway platform accumulates more entries, exploring data augmentation and domain-adaptive pretraining, and pursuing cross-institutional collaborations to increase sample diversity. These steps would strengthen model robustness and improve its ability to support nuanced educational decision-making. Although 4-level predictions should be interpreted with caution, they can still offer valuable insights for faculty development and formative assessment when combined with human judgment.

Second, as with all text-based evaluations, important nonverbal cues and dynamic interpersonal interactions are not captured. Future work could extend beyond text-based analysis by integrating audio and video data with NLP. Multimodal inputs would capture tone, pacing, and nonverbal cues, complementing narrative content and offering a more holistic view of feedback interactions. This approach could strengthen competency-based medical education by providing richer insights to guide faculty development and resident learning.

Third, although improvements were observed in the narrative quality, this study did not directly measure faculty engagement or sustained educational change. Future research should examine how NLP-generated insights might be incorporated into faculty development initiatives and longitudinal assessment strategies to determine whether they enhance faculty participation and support lasting improvements in feedback and reflection quality.

Finally, the possibility of a Hawthorne effect should be considered. The awareness of being evaluated may have influenced improvements in reflection and feedback quality [45,46]. Complementary qualitative research such as interviews or focus groups with residents and faculty could elucidate underlying motivations and perceptions, providing a richer perspective on behavioral change.

### Conclusions

This study demonstrates that BERT-based NLP, when applied with structured contextual inputs, can effectively evaluate the quality of multilingual resident reflections and faculty feedback in WBAs. The model achieved moderate to high accuracy, particularly in binary classification, suggesting its utility as a scalable adjunct to human evaluation. While not a substitute for expert judgment, NLP can facilitate large-scale monitoring of narrative quality and enhance the analysis of formative feedback in CBME. The progressive improvement in the narrative quality over 4 years highlights the value of structured EPA frameworks and digital platforms such as Emyway in promoting reflective practice and faculty development. Future research should explore the generalizability of this approach across medical specialties and investigate the integration of multimodal data to further enhance assessment validity and educational outcomes.

## Appendix

Multimedia Appendix 1 Taiwan Society of Otorhinolaryngology–Head and Neck Surgery Entrustable Professional Activities Assessment Framework for Resident Physician Training, second edition.

Multimedia Appendix 2 Quantified agreement results (interrater reliability) for expert scoring.

Multimedia Appendix 3 Logistic regression, support vector machine, and bidirectional encoder representations from transformers codes in the Google Colaboratory.

Multimedia Appendix 4 Sample outputs from the bidirectional encoder representations from transformers model for classifying narrative quality in resident reflections and faculty feedback.

Multimedia Appendix 5 Distribution of numbers (percentages) of 4-level and 2-level quality ratings for resident reflections and faculty feedback across pilot year (2021-2022), 2022-2023, 2023-2024, and 2024-2025.

Multimedia Appendix 6 Detailed process and results for evaluating resident reflections and faculty feedback quality by using ChatGPT-4o.

## Abbreviations

BERT: bidirectional encoder representations from transformers
CBME: competency-based medical education
EPA: Entrustable Professional Activity
LR: logistic regression
NLP: natural language processing
SVM: support vector machine
TSO-HNS: Taiwan Society of Otorhinolaryngology–Head and Neck Surgery
WBA: workplace-based assessment

## Data Availability

The datasets used and analyzed during this study are available from the corresponding author on reasonable request.
