# Supplementary material for: Automated Evaluation of Reflection and Feedback Quality in Workplace-Based Assessments by Using Natural Language Processing: Cross-Sectional Competency-Based Medical Education Study
Source: JMIR Med Educ. 2025 Oct 22;11:e81718. doi: 10.2196/81718 (PMC12590046; doi:10.2196/81718)
Supplement: Multimedia Appendix 1 [file mededu_v11i1e81718_app1.pdf]

Multimedia Appendix 1

Taiwan Society of Otorhinolaryngology–Head and Neck Surgery Entrustable Professional Activities Assessment Framework for Resident Physician Training

Second Edition

20210508V2

The Entrustable Professional Activities assessment framework
integrates the core competencies and subcompetencies
in the "Otolaryngology Milestone Project."

BY

Taiwan Society of Otorhinolaryngology–Head and Neck Surgery Working Group on the development and implementation of Competency-Based Medical Education

Adviser: Pei-Jen Lou, Pen-Yuan Chu, Te-Huei Yeh, Hui-Ching Chuang
CBME Working Group Conveners: Jeng-Wen Chen, Wei-Chun Hsu
CBME Working Group Members:
Sen-Tien Tsai, Pa-Chun Wang, Chia -Der Lin, Chung-Han Hsin, Shih-An Liu, Cheng-Ming Hsu, Yen-Bin Hsu, Li-Jen Hsin, Li-Jen Liao, Po-Hsuan Wu, Po-Wen Cheng, Ming-Shao Tsai, Hung-Che Lin, Ching-Hui Hsu, Li-Ang Lee, Che-Wei Wu

Table of Contents

Framework of the Entrustable Professional Activities (EPAs) Assessment for Otolaryngologic Resident Physician Training	2
Blueprint of the Integrated Milestones and Entrustable Professional Activities (EPAs) Assessment for Otolaryngologic Resident Physician Training	3
General Description and User's Guide for the EPAs	4
EPA01(Airway) Assessing and managing patients with airway presentations	7
EPA02(FB) Assessing and managing patients with suspicious foreign body presentations	10
EPA03(Bleeding) Assessing and managing patients with upper aerodigestive tract bleeding presentations	13
EPA04(Vertigo) Assessing and managing patients with vertigo	16
EPA05(Infection) Assessing and managing patients with head and neck infections	19
EPA06(H&N) Assessing and managing patients with head and neck masses	22
EPA07(Ear) Assessing and managing patients with ear and hearing diseases	25
EPA08(Sinonasal) Assessing and managing patients with sinonasal diseases	28
EPA09(Larynx) Assessing and managing patients with laryngopharyngeal diseases (voice/speech/language/dysphagia)	32
EPA10(SDB) Assessing and managing patients with sleep-disordered breathing	35
EPA11(Plasty) Assessing and managing patients with facial plastic and reconstructive surgery	38

FRAMEWORK of Taiwan Society of Otorhinolaryngology–Head and Neck Surgery

Entrustable Professional Activities (EPAs) Assessment for Resident Physician Training (20210508V2)

EPA Code	EPA Title	Focused Observation in the Workplace-Based Evaluations				
		Outpatient Clinic	Ward (ICU)	Emergency Department	Operating Room	Consultation
**EPA01(Airway)**	**Assessing and managing patients with airway presentations**	✪	✪	✪✪	✪	✪✪
**EPA02(FB)**	**Assessing and managing patients with suspicious foreign body**	✪✪	✪	✪✪	✪	✪
**EPA03(Bleeding)**	**Assessing and managing patients with upper aerodigestive tract bleeding presentations**	✪	✪	✪✪	✪	✪✪
**EPA04(Vertigo)**	**Assessing and managing patients with vertigo**	✪✪	✪	✪	✪	✪
**EPA05(Infection)**	**Assessing and managing patients with head and neck infections**	✪✪	✪✪	✪✪	✪	✪
**EPA06(H&N)**	**Assessing and managing patients with head and neck masses**	✪✪	✪✪	✪	✪✪	✪
**EPA07(Ear)**	**Assessing and managing patients with ear and hearing diseases**	✪✪	✪✪	✪	✪✪	✪
**EPA08(Sinonasal)**	**Assessing and managing patients with sinonasal diseases**	✪✪	✪✪	✪	✪✪	✪
**EPA09(Larynx)**	**Assessing and managing patients with laryngopharyngeal diseases (voice/speech/language/dysphagia)**	✪✪	✪✪	✪	✪✪	✪
**EPA10(SDB)**	**Assessing and managing patients with sleep-disordered breathing**	✪✪	✪✪	✪	✪✪	✪
**EPA11(Plasty)**	**Assessing and managing patients with facial plastic and reconstructive surgery**	✪✪	✪✪	✪	✪✪	✪

✪✪ Indicates that the clinical instructors **MUST** observe the learners in these workplaces or scenarios before awarding them independence in the EPA task.

✪ Indicates that the clinical instructors **MAY** observe the learners in these workplaces or scenarios to build up entrustment for independence in the EPA task.

BLUEPRINT of the Taiwan Society of Otorhinolaryngology–Head and Neck Surgery

Integrated Milestones and Entrustment Professional Activities for Resident Physician Training (20210508V2)

Milestones 23 Sub-Competencies	Code	Entrustable Professional Activities (EPAs)										
		EPA1	EPA2	EPA3	EPA4	EPA5	EPA6	EPA7	EPA8	EPA9	EPA10	EPA11
		Airway	FB	Bleeding	Vertigo	Infection	H&N	Ear	Sinonasal	Larynx	SDB	Plasty
Airway Emergency and Management	PC1	✪✪	✪	✪		✪✪	✪			✪		
Foreign Body Management	PC2	✪✪	✪✪			✪		✪	✪	✪		
Head and Neck Neoplasm	PC3	✪		✪		✪	✪✪	✪	✪	✪		✪
Otologic Disease	PC4		✪		✪✪	✪	✪	✪✪				
Rhinologic Disease	PC5		✪	✪✪		✪	✪		✪✪		✪	✪
Laryngologic Disease	PC6	✪✪	✪	✪		✪✪	✪			✪✪	✪	
Pediatric Otolaryngology	PC7	✪	✪	✪		✪✪		✪	✪	✪	✪✪	
Facial Plastic and Reconstructive Surgery	PC8			✪		✪	✪	✪	✪	✪		✪✪
Sleep	PC9	✪		✪					✪	✪	✪✪	
Anatomy	MK1	✪	✪	✪	✪	✪	✪✪	✪✪	✪✪	✪✪	✪	✪
Allergy	MK2	✪							✪✪	✪	✪	
Pathophysiology	MK3				✪✪		✪✪	✪✪	✪✪	✪	✪	
Patient Safety and Quality Improvement	SBP1	✪✪	✪✪	✪✪	✪✪	✪✪	✪✪	✪✪	✪✪	✪✪	✪✪	✪✪
System Navigation for Patient-Centered Care	SBP2	✪✪	✪	✪	✪	✪	✪✪	✪	✪	✪	✪✪	✪
Physician Role in Health Care Systems	SBP3	✪✪	✪	✪✪	✪	✪	✪✪	✪	✪	✪	✪✪	✪
Evidence-Based and Informed Practice	PBLI1	✪	✪	✪	✪✪	✪	✪✪	✪	✪	✪	✪	✪
Reflective practice and Commitment to Personal Growth	PBLI2	✪	✪	✪	✪	✪	✪	✪	✪	✪	✪	✪
Professional Behavior and Ethical Principles	PROF1	✪	✪	✪	✪	✪	✪	✪	✪	✪	✪	✪
Accountability/Conscientiousness	PROF2	✪✪	✪✪	✪✪	✪✪	✪✪	✪✪	✪✪	✪✪	✪✪	✪✪	✪✪
Knowledge of Systemic and Individual Factors of Well-Being	PROF3	✪	✪	✪	✪	✪	✪	✪	✪	✪	✪	✪
Patient- and Family-Centered Communication	ICS1	✪✪	✪✪	✪✪	✪✪	✪✪	✪✪	✪✪	✪✪	✪✪	✪✪	✪✪
Interprofessional and Team Communication	ICS2	✪✪	✪✪	✪	✪	✪	✪✪	✪	✪	✪	✪✪	✪
Communication within Health Care Systems	ICS3	✪✪	✪	✪	✪	✪	✪✪	✪	✪	✪	✪	✪

✪✪ Indicates that these milestone core competencies and subcompetencies are **IMPORTANT** for this EPA.

✪ Indicates that these milestone core competencies and subcompetencies are **USEFUL** for this EPA.

General Description and User's Guide for the EPAs

The Taiwan Society of Otorhinolaryngology–Head and Neck Surgery Integrated Entrustable Professional Activities Assessment Framework (TSOHNS-EPA) is developed based on Professor Olle ten Cate's recommended description of an entrustable professional activity: AMEE Guide No. 140. (*Med Teach* 2021;43:1106-1114. Doi:10.1080/0142159X.2020.1838465) This AMEE Guide described eight sections of a full EPA description, and provides explanations and justifications for each. These sections were: title; specification and limitations; risks in case of failure; most relevant competency domains; knowledge, skills, attitudes and experiences; information sources to assess progress and support summative entrustment; entrustment-supervision level expected at which stage of training; and time period to expiration if not practiced.

<table>
<tr><td colspan="2">1. EPA title</td></tr>
<tr><td colspan="2">An adequate title that make the EPA comprehensible to learners, clinical instructors, regulators, examination boards, and nursing staff, etc.</td></tr>
<tr><td colspan="2">2. Specification and limitations</td></tr>
<tr><td rowspan="2">A clearly defined clinical context without ambiguity:
1. A detailed description of the core EPAs to avoid confusion or differences in interpretation when assessing or awarding the EPA. For example, "assessing and managing patients with presenting symptoms that were related to airway obstruction..."
2. EPAs can be single tasks with components that may be chronological and can be detailed in a bulleted list.</td><td>Limitations:
Elements or situations in which the individual is not necessarily qualified to perform when certified for this EPA, which further serve to clarify its scope to the outside world. (Clinical boundary of the EPAs)</td></tr>
<tr><td>Clinical situations that are necessary (but not limited) to complete training:
By the scope of the situation (or disease), the EPA lists relevant workplaces for observation: When this EPA is used for a summative assessment to determine the competence of an otolaryngologist, which clinical situations are essential for the otolaryngologist to demonstrate their competencies that are required by the local communities. However, one should not limit the ability to observe and evaluate the items listed, and any other circumstances that meet the description of this EPA task should also be taken as a reference for observation and judgment. (Coverage requirements for the EPAs)</td></tr>
<tr><td colspan="2">3. Potential risks in case of failure</td></tr>
<tr><td colspan="2">Descriptions:
1. This section is used to specify and understand (to some extent) the adverse events that can occur when the task is not performed properly.
2. Potential risks involved several aspects of failure, including diagnosis, management, communications, and different roles, such as patients, family, team, or social perception.</td></tr>
<tr><td colspan="2">4. Most relevant competency domains</td></tr>
<tr><td colspan="2">This section serves to make the connection between the EPA and the relevant competency framework by identifying the most relevant domains or roles. Those competencies and sub-competencies can subsequently guide the development of curriculum, assessment tools, learning plans, etc.</td></tr>
<tr><td colspan="2">5. Required knowledge, skills, attitudes and experiences to allow for summative entrustment</td></tr>
<tr><td colspan="2">This section lists required knowledge, skills, attitudes, and experiences to guide learners toward the expectations that supervisors and programs may hold as criteria for readiness for decreased supervision. Relevant knowledge, skills, and attitudes, necessary experience is also involved. In</td></tr>
</table>

addition, this will be used as a reference for the curriculum design of the training unit to facilitate the creation of relevant core courses before the clinical implementation of the EPAs.		
**Knowledge:**<br>List the knowledge required to perform the task.<br>For example: to manage symptoms related to airway obstruction, you need to know the anatomy of the airway, the possible manifestations of priority and others.	**Skills and attitudes:**<br>List the skills and professional attitudes required to perform the task.<br>For example: handling patients with suspected upper respiratory and digestive tract foreign bodies requires focused medical history taking and examination skills; handling patients with sleep-disordered breathing requires teamwork skills and attitudes.	**Required experiences**:<br>List the certifications or disciplines required to perform the task.<br>For example, to perform surgery independently to treat sinus diseases, one must go through FESS dissection course, etc., to perform surgery independently to treat middle ear or mastoid disease, one must go through temporal bone dissection course, etc.
**6. Information sources to assess progress and support summative entrustment**		
The EPA needs a blueprint to maintain the flexibility of the current training program's planning and to continue to build consensus. This version of EPAs recommends the planning of the assessment blueprint for the following six types of assessment tools (the CBME core group members consented to follow the principle of "at least two clinical teachers, at least two observations at different points of time," but not to that limited numbers of evaluations, multi-point, multi-oriented observations are the focus of EPAs assessment):<br>1. Knowledge tests<br>2. Simulation<br>3. Case-based discussions (CbD) or Entrustment-based discussion (EbD)<br>4. Short practice observations (SPOs, eg, ad-hoc EPA-based tool, mini-CEX, Direct Observation Procedural Skill, DOPS)<br>5. Long practice observations (LPOs, eg, multi-source feedback, MSF)<br>6. Products (eg, reports, medical record, portfolio, case-log<br>Every training program should arrange the EPA evaluation based on the principles of multiple assessment tools (using various assessment tools according to the descriptions of each EPA task) and numerous points (arranging enough assessment times) to achieve the effectiveness of workplace-based observations and the reliability of summative assessment. There is no evidence of the number of time-dependent assessments required for each EPA to achieve entrustment or the validity at the early stage of the implementation. This version of Otolaryngology–Head and Neck Surgery EPAs is based only on individual characteristic, recommendations on the application of these six types of assessment tools, the information required to demonstrate progress. Upcoming consensus for the required number of evaluations and level of entrustment will be made after collecting data from initial implementation.		
**7. Entrustment / supervision level expected at which stage of training**		
This section states for which level the entrustment decision is being made at which stage of training. Five main levels have been described: Level 1: the learner is allowed to be present and observe, not to enact an EPA; Level 2: the learner is allowed to execute the EPA with direct, pro-active supervision, present in the room; Level 3: the learner is allowed to carry out the EPA without a supervisor in the room, but quickly available if needed, i.e. with indirect, reactive, supervision; Level 4: the learner is allowed to work unsupervised; Level 5: the learner is allowed to provide supervision to more junior learners.		

**8. Time period to expiration if not practiced**
The skill to perform an EPA task can decay if an activity is not practiced or just by prolonged routine practice. Considering the possible impact of the decline in proficiency on the quality of medical care when relevant professional activities have not been implemented for a defined period of time. This section describes how long it can last if the learner temporarily leaves the training with the original level of entrustment.

Otorhinolaryngology–Head and Neck Surgery EPA01 (Airway)

<table>
<tr><td colspan="2">1. EPA Title</td></tr>
<tr><td colspan="2">Assessing and managing patients with airway presentations</td></tr>
<tr><td colspan="2">2. Specification and limitations</td></tr>
<tr><td rowspan="2">Outpatient clinic/emergency department/ward patient (adult and child) presenting with symptoms of airway obstruction:
1. Focused history taking and physical examination
2. Application of diagnostic examination and differential diagnosis
3. Evaluating, and identifying of the necessity and urgency of airway examinations, management, or surgery (including the evaluation and assessment of complicated airway)
4. Establishing artificial airway (intubation or tracheotomy)
5. Evaluating, and identifying of the necessity of oxygen supplement or artificial airway
6. Emergency airway management or postoperative care
7. Follow-up treatment or follow-up plan
8. Managing complication
9. Referral using systemic resources
10. Airway care-associated counseling
11. Keeping medical record</td><td>Limitation:
Only applicable to assessing and managing patients with ear/nose/throat/and head and neck tumors or difficult intubation.</td></tr>
<tr><td>Clinical situations required (but not limited to) observation for completion of training:
1. Mirror/headlight or endoscopic airway assessment
2. Fiberoptic nasopharyngeal endoscopic airway examination
3. Image interpretation and explanation
4. Endotracheal intubation
5. Tracheostomy case
6. Tracheostomy cannula change case
7. Systemic referral and handover to the next shift
8. Postoperative care or managing cases with complications
9. Laryngotracheal endoscopic and bronchial airway examination and endoscopic surgery (observation)
10. Laryngotracheal reconstruction and plastic surgical cases (observation)</td></tr>
<tr><td colspan="2">3. Potential risks in case of failure</td></tr>
<tr><td colspan="2">Many patients suffer complications that could otherwise be avoided (such as hypoxia, respiratory failure, airway obstruction, and even death). Differential diagnosis errors or disposal of waste of medical resources, complications, improper disability or even death, medical teams facing disputes or litigation pressure, loss of society’s trust in the profession of otolaryngology, head, and neck surgery, etc.</td></tr>
<tr><td colspan="2">4. Most relevant competency domains</td></tr>
</table>

**Patient Care**	**Medical Knowledge**	**Professionalism**	**Interpersonal & Communication Skills**	**Practice-based learning and improvement**	**System-based Practice**
✪✪<br>PC1<br>Airway Emergency and Manageme nt<br>PC2<br>Foreign Body Manageme nt PC6<br>Laryngolog i-c Disease	✪<br>MK1<br>Anatomy<br>MK2<br>Allergy	✪✪<br>PROF2<br>Accountability/Con scientiousness<br>✪<br>PROF1<br>Professional Behavior and Ethical Principles<br>PROF3<br>Knowledge of Systemic and Individual Factors of Well-Being	✪✪<br>ICS1<br>Patient- and Family-Centered Communication<br>ICS2<br>Interprofessional and Team Communication<br>ICS3<br>Communication within Health Care Systems	✪<br>PBLI1<br>Evidence-Based and Informed Practice<br>PBLI2<br>Reflective practice and Commitment to Personal Growth	✪✪<br>SBP1<br>Patient Safety and Quality Improvement<br>SBP2<br>System Navigation for Patient-Centered Care<br>SBP3<br>Physician Role in Health Care Systems

✪<br>PC3<br>Head and Neck Neoplasm<br>PC7<br>Pediatric Otolaryngo lo-gy<br>PC9<br>Sleep					

**5. Required knowledge, skills, attitudes and experiences to allow for summative entrustment**		
**Knowledge:**<br>1. Upper respiratory and digestive tract anatomy,<br>2. Larynx, trachea, and neck anatomy,<br>3. Interpretation of respiratory physiological examination results<br>4. Interpretation of image studies<br>5. Common complications and their management,<br>6. Other internal medicine/surgery/alter native treatment methods,<br>7. Follow up plan	**Skills and Attitudes:**<br>1. Focused history taking<br>2. Mirror/headlight or endoscopy operation skills,<br>3. Fiberoptic nasopharyngeal endoscope skills,<br>4. Laryngoscopy and intubation skills,<br>5. Tracheostomy,<br>6. Airway surgery, preoperative, intraoperative, and postoperative care, and health education,<br>7. Perform bronchoscopy<br>8. Timely detection of potential symptoms of complications<br>9. Timely referral using resources<br>10. Explain the condition, informed consent, inform bad news (unexpected results) and other medical communication skills,<br>11. Teamwork skills and attitude,<br>12. Patient-centered attitude	**Required experience:**<br>1. Basic science course of otolaryngology, head, and neck surgery,<br>2. Airway core curriculum<br>3. Children's advanced airway course
**6. Information sources to assess progress and support summative entrustment**		
The EPA needs a blueprint to maintain the flexibility of the current training program's planning and to continue to build consensus. This version of EPAs recommends the planning of the assessment blueprint for the following six types of assessment tools (the CBME core group members consented to follow the principle of "at least two clinical teachers, at least two observations at different points of time," but not to that limited numbers of evaluations, multi-point, multi-oriented observations are the focus of EPAs assessment):<br>1. Written test (knowledge test): To design a valid knowledge test according to the connotation of the EPA task, and to confirm learners with required knowledge performing assessment and management of patients with airway presentations. Type of question included, but not limited to those testing understanding, analyzing, judgement, and application.<br>2. Simulation: To design a valid situational simulation test to describe the project's task and test the students' "situational ability."<br>3. Case-based discussion: To test learner's clinical thinking, reasoning, logic, attitude, etc., related to the EPA task. The recommended tools are CBD and EbD.		

4. Short-practice observation (short-practice observation): Observe and evaluate student's performance in a task in the workplace. Recommended tools include DOPS, mini-CEX, EPAs, etc.
5. Long-practice observation and evaluation: Observe and evaluate the actual performance of trainees in the workplace during the previous period. This observation can avoid the "Hawthorne effect" of direct observation and evaluation in the workplace for a short period. It is recommended to collect feedback from peers, colleagues, or patients through "multi-source feedback" to confirm student's performance in terms of accountability, communication, teamwork, and resilience.
6. Portfolio: To record the learning progress, including quantitative (for example, number of cases, number of operations) and qualitative (self-evaluation, experience, reflection) content, which can be used as a reference for accumulation of learning experience and self-learning ability. The recommended tools include medical certificate, clinic and inpatient medical records, surgical records, case log, journal reading, case-report, research paper publication.

7. Entrustment / supervision level expected at which stage of training

Schedule Entrustment-Supervision Level	Level 1	Level 2	Level 3	Level 4	Level 5
Before entering R2 training (R1)		✪			
Before entering R3 training (R2)			✪		
Before entering R4 training (R3)			✪		
Before entering R5 training (R4)				✪	
Before completing the training (R5)					✪

Level 1	the learner is allowed to be present and observe, not to enact an EPA
level 2	the learner is allowed to execute the EPA with direct, pro-active supervision, present in the room
level 3	the learner is allowed to carry out the EPA without a supervisor in the room, but quickly available if needed, i.e. with indirect, reactive, supervision
level 4	the learner is allowed to work unsupervised
level 5	the learner is allowed to provide supervision to more junior learners

8. Time period to expiration if not practiced

The entrustment / supervision level should be re-evaluated if relevant professional activities have not been implemented for 1 year.

Otorhinolaryngology–Head and Neck Surgery EPA02 (FB)

1. EPA Title

Assessing and managing patients with suspicious foreign body

2. Specification and limitations

Main complaint for patients (adults and children) in clinic/emergency/ward settings with symptoms related to upper respiratory and digestive tract (including esophagus) or ear foreign bodies:<br>1. Focused examination<br>2. Use of diagnostic tests to identify causes<br>3. Preoperative explanation<br>4. Execution of procedures or surgeries related to foreign body removal<br>5. Postoperative care<br>6. Establishment of treatment or follow-up plan<br>7. Management of complications<br>8. Utilization of system resource referrals<br>9. Explanation and health education after foreign body removal<br>10. Explanation and health education in the absence of foreign bodies<br>11. Medical records	Limitation: Applicable only to the assessment and management of patients with ear, nose, throat, or esophageal foreign bodies, excluding tracheal or bronchial foreign bodies (observation only).<br><br>Clinical situations necessary (not limited to) to complete training:<br>1. Foreign body removal cases under headlight/headlamp or otomicroscope<br>2. Esophagoscopy foreign body removal cases<br>3. Cases involving referrals and handovers within the system<br>4. Postoperative care or management of related complications cases<br>5. Explanation and health education cases after foreign body removal<br>6. Follow-up cases in the absence of foreign bodies<br>7. Bronchoscopy foreign body removal cases (observation)Evaluation of the Lower Respiratory Tract of Frontal Reflector/Headlamp or Endoscope

3. Potential risks in case of failure

Patients may suffer from otherwise avoidable complications (such as bleeding, infection, perforation, respiratory obstruction, or even death). Misdiagnosis or improper management can lead to the waste of medical resources, improper handling of complications may result in disability or death, the medical team may face disputes or litigation pressure, and the trust in the ENT (ear, nose, throat, and head and neck surgery) profession by society may be compromised.

4. Most relevant competency domains

**Patient Care**	**Medical Knowledge**	**Professionalism**	**Interpersonal & Communication Skills**	**Practice-based learning and improvement**	**System-based Practice**
✪✪<br>PC2<br>Foreign Body Management<br>✪<br>PC1<br>Airway Emergency and Management<br>PC4<br>Otologic Disease<br>PC5	✪<br>MK1<br>Anatomy	✪✪<br>PROF2<br>Accountability/ Conscientiousnes-s<br>✪<br>PROF1<br>Professional Behavior and Ethical Principles<br>PROF3<br>Knowledge of Systemic and Individual Factors	✪✪<br>ICS1<br>Patient- and Family-Centered Communication<br>ICS2<br>Interprofessional and Team Communication<br>✪<br>ICS3<br>Communication	✪<br>PBLI1<br>Evidence-Based and Informed Practice<br>PBLI2<br>Reflective practice and Commitment to Personal	✪✪<br>SBP1<br>Patient Safety and Quality Improvement<br>✪<br>SBP2<br>System Navigation for Patient-Centered Care<br>SBP3<br>Physician

Rhinologic Disease<br>PC6<br>Laryngologic Disease<br>PC7<br>Pediatric Otolaryngolog-y		of Well-Being	within Health Care Systems	Growth	Role in Health Care Systems

5. Required knowledge, skills, attitudes and experiences to allow for summative entrustment		
**Knowledge:**<br>1. Anatomy of the upper respiratory and digestive tracts<br>2. Ear anatomy<br>3. Interpretation of radiological examination reports<br>4. Common complications and their management methods<br>5. Medical/surgical/alternative treatment methods<br>6. Formulation of follow-up plans	**Skills and Attitudes:**<br>1. Focused history taking<br>2. Operation techniques for head mirror/headlamp or otomicroscope<br>3. Nasopharyngeal fiber endoscopy examination<br>4. Selection of instruments and foreign body removal techniques<br>5. Preoperative, intraoperative, and postoperative care and health education<br>6. Performing esophagoscopy<br>7. Detecting complications and providing timely management<br>8. Utilizing resources for timely referrals<br>9. Medical communication skills for explaining conditions, obtaining informed consent, and delivering bad news (unexpected outcomes)<br>10. Teamwork skills and attitude<br>11. Patient-centered attitude	**Required experience:**<br>1. Essential core curriculum for ear, nose, and neck surgery<br>2. Respiratory core curriculum
**6. Information sources to assess progress and support summative entrustment**		
The EPA needs a blueprint to maintain the flexibility of the current training program's planning and to continue to build consensus. This version of EPAs recommends the planning of the assessment blueprint for the following six types of assessment tools (the CBME core group members consented to follow the principle of "at least two clinical teachers, at least two observations at different points of time," but not to that limited numbers of evaluations, multi-point, multi-oriented observations are the focus of EPAs assessment):<br>1. Written test (knowledge test): To design a valid knowledge test according to the connotation of the EPA task, and to confirm learners with required knowledge performing assessment and management of patients with airway presentations. Type of question included, but not limited to those testing understanding, analyzing, judgement, and application.<br>2. Simulation: To design a valid situational simulation test to describe the project's task and test the students' "situational ability."<br>3. Case-based discussion: To test learner's clinical thinking, reasoning, logic, attitude, etc., related to the EPA task. The recommended tools are CBD and EbD.		

4. Short-practice observation (short-practice observation): Observe and evaluate student's performance in a task in the workplace. Recommended tools include DOPS, mini-CEX, EPAs, etc.
5. Long-practice observation and evaluation: Observe and evaluate the actual performance of trainees in the workplace during the previous period. This observation can avoid the "Hawthorne effect" of direct observation and evaluation in the workplace for a short period. It is recommended to collect feedback from peers, colleagues, or patients through "multi-source feedback" to confirm student's performance in terms of accountability, communication, teamwork, and resilience.
6. Portfolio: To record the learning progress, including quantitative (for example, number of cases, number of operations) and qualitative (self-evaluation, experience, reflection) content, which can be used as a reference for accumulation of learning experience and self-learning ability. The recommended tools include medical certificate, clinic and inpatient medical records, surgical records, case log, journal reading, case-report, research paper publication.

7. Entrustment / supervision level expected at which stage of training

Schedule Entrustment-Supervision Level	Level 1	Level 2	Level 3	Level 4	Level 5
Before entering R2 training (R1)		✪			
Before entering R3 training (R2)			✪		
Before entering R4 training (R3)			✪		
Before entering R5 training (R4)				✪	
Before completing the training (R5)					✪

Level 1	the learner is allowed to be present and observe, not to enact an EPA
level 2	the learner is allowed to execute the EPA with direct, pro-active supervision, present in the room
level 3	the learner is allowed to carry out the EPA without a supervisor in the room, but quickly available if needed, i.e. with indirect, reactive, supervision
level 4	the learner is allowed to work unsupervised
level 5	the learner is allowed to provide supervision to more junior learners

8. Time period to expiration if not practiced

The entrustment / supervision level should be re-evaluated if relevant professional activities have not been implemented for 1 year.

Otorhinolaryngology–Head and Neck Surgery EPA03 (Bleeding)

<table>
<tr><td colspan="6">1. EPA Title</td></tr>
<tr><td colspan="6">Assessing and managing patients with upper aerodigestive tract bleeding presentations</td></tr>
<tr><td colspan="6">2. Specification and limitations</td></tr>
<tr><td colspan="3" rowspan="2">Otorhinolaryngology and head and neck (including oral) bleeding-related symptoms clinic emergency/inpatient with the main complaint (Adults and children):
1. The focused diagnosis
2. Uses diagnostic examinations to identify the bleeding location and the cause.
3. Monitoring patient vital signs.
4. Immediate hemostasis treatment to
5. Fill the bleeding location.
6. Judging and explaining the necessity of hemostasis treatment (vascular embolization) or surgery (vascular ligation) under anesthesia.
7. Provide treatment or follow the plan
8. Stop bleeding aftercare (blood pressure control, drip, blood transfusion, etc.)
9. Complications disposal
10. Use system resources referred
11. After repeatedly stopping bleeding prevention bleeding in health education and illustrate
12. Medical records</td><td colspan="3">Limitation: Only applicable to ear/nose/throat/and head and neck tumors or difficulties intubation. Evaluation and treatment of patients.</td></tr>
<tr><td colspan="3">Clinical situations necessary (not limited to) to complete training:
the amount of microscopic view with a reflecting mirror bleeding lesions / or the headlight is determined
1. Diagnosis of Hemorrhage Focus by Frontal Reflector/Headlamp or Endoscope
2. Case of Nasal Hemorrhage Occlusion (Including Anterior and Posterior Nasal Occlusion)
3. The Case of Filling after Oral and Throat Hemorrhage
4. Treatment of head and neck tumors with massive hemorrhage
5. Angiographic and Embolization Cases (Observation)
6. Case of Surgical Hemostasis (Observation)
7. Packing removal and subsequent care items
8. System Down-Transfer and Handover Cases
9. Cases of Postoperative Care or Treatment of Related Complications</td></tr>
<tr><td colspan="6">3. Potential risks in case of failure</td></tr>
<tr><td colspan="6">Patients may suffer from the inevitable complications (such as anemia, shock, infection, respiratory obstruction, and even death due to massive hemorrhage), the medical resources waste due to the error of differential diagnosis or treatment, the failure or death due to improper treatment of the complications, the medical team is faced with disputes or litigation pressure. Society has lost trust in the specialty of ear, nose, and neck surgery.</td></tr>
<tr><td colspan="6">4. Most relevant competency domains</td></tr>
<tr><td>Patient Care
✪✪
<u>PC5</u>
Rhinologic Disease
✪
<u>PC1</u>
Airway Emergency and</td><td>Medical Knowledge
✪
<u>MK1</u>
Anatomy</td><td>Professionalism
✪✪
<u>PROF2</u>
Accountability/Conscientiousness
✪
<u>PROF1</u>
Professional Behavior and Ethical Principles</td><td>Interpersonal & Communication Skills
✪✪
<u>ICS1</u>
Patient- and Family-Centered Communication
✪
<u>ICS2</u></td><td>Practice-based learning and improvement
✪
<u>PBLI1</u>
Evidence-Based and Informed Practice
<u>PBLI2</u></td><td>System-based Practice
✪✪
<u>SBP1</u>
Patient Safety and Quality Improvement
<u>SBP3</u>
Physician Role in Health Care Systems</td></tr>
</table>

Management<br>PC3<br>Head and Neck Neoplasm<br>PC6<br>Laryngologic Disease<br>PC7<br>Pediatric Otolaryngolog-y<br>PC8<br>Facial Plastic and Reconstructive Surgery<br>PC9<br>Sleep		PROF3<br>Knowledge of Systemic and Individual Factors of Well-Being	Interprofessional and Team Communication<br>ICS3<br>Communication within Health Care Systems	Reflective practice and Commitment to Personal Growth	✪<br>SBP2<br>System Navigation for Patient-Centered Car

5. Required knowledge, skills, attitudes and experiences to allow for summative entrustment		
**Knowledge:**<br>1. Anatomy of upper respiratory digestive tract<br>2. Ear anatomy<br>3. Interpretation of Imaging Inspection Report<br>4. Common complications and treatment methods<br>5. Internal/surgical/alternative treatment<br>6. List tracking plans	**Skills and Attitudes:**<br>1. Focus Medical History Inquiry<br>2. Technique for operating frontal reflector/headlamp or auricular mirror<br>3. Endoscopic examination of nasopharynx fibers<br>4. Instrument Selection and Foreign Body Removal Skill<br>5. Nursing and Nursing Care before, during, and after Operation<br>6. Performing a Gastroscopy<br>7. Detect complications and dispose of them immediately<br>8. Using Resources to Transfer Timely<br>9. Explain illness, informed consent, terrible news (non-expected result), etc. Medical communication skills<br>10. Team Cooperation Skills and Attitudes<br>11. patient-centered attitude	**Required experience:**<br>1. Essential core curriculum for ear, nose, and neck surgery<br>2. Respiratory core curriculum

6. Information sources to assess progress and support summative entrustment

The EPA needs a blueprint to maintain the flexibility of the current training program's planning and to continue to build consensus. This version of EPAs recommends the planning of the assessment blueprint for the following six types of assessment tools (the CBME core group members consented to follow the principle of "at least two clinical teachers, at least two observations at different points of time," but not to that limited numbers of evaluations, multi-point, multi-oriented observations are the focus of EPAs assessment):

1. Written test (knowledge test): To design a valid knowledge test according to the connotation of the EPA task, and to confirm learners with required knowledge performing assessment and management of patients with airway presentations. Type of question included, but not limited to those testing understanding, analyzing, judgement, and application.
2. Simulation: To design a valid situational simulation test to describe the project's task and test the students' "situational ability."

3. Case-based discussion: To test learner's clinical thinking, reasoning, logic, attitude, etc., related to the EPA task. The recommended tools are CBD and EbD.
4. Short-practice observation (short-practice observation): Observe and evaluate student's performance in a task in the workplace. Recommended tools include DOPS, mini-CEX, EPAs, etc.
5. Long-practice observation and evaluation: Observe and evaluate the actual performance of trainees in the workplace during the previous period. This observation can avoid the "Hawthorne effect" of direct observation and evaluation in the workplace for a short period. It is recommended to collect feedback from peers, colleagues, or patients through "multi-source feedback" to confirm student's performance in terms of accountability, communication, teamwork, and resilience.
6. Portfolio: To record the learning progress, including quantitative (for example, number of cases, number of operations) and qualitative (self-evaluation, experience, reflection) content, which can be used as a reference for accumulation of learning experience and self-learning ability. The recommended tools include medical certificate, clinic and inpatient medical records, surgical records, case log, journal reading, case-report, research paper publication.

7. Entrustment / supervision level expected at which stage of training

Schedule Entrustment-Supervision Level	Level 1	Level 2	Level 3	Level 4	Level 5
Before entering R2 training (R1)		✪			
Before entering R3 training (R2)			✪		
Before entering R4 training (R3)			✪		
Before entering R5 training (R4)				✪	
Before completing the training (R5)					✪

Level 1	the learner is allowed to be present and observe, not to enact an EPA
level 2	the learner is allowed to execute the EPA with direct, pro-active supervision, present in the room
level 3	the learner is allowed to carry out the EPA without a supervisor in the room, but quickly available if needed, i.e. with indirect, reactive, supervision
level 4	the learner is allowed to work unsupervised
level 5	the learner is allowed to provide supervision to more junior learners

8. Time period to expiration if not practiced

The entrustment / supervision level should be re-evaluated if relevant professional activities have not been implemented for 1 year.

Otorhinolaryngology–Head and Neck Surgery EPA04 (Vertigo)

**1. EPA Title**
Assessing and managing patients with vertigo

**2. Specification and limitations**	
clinic/emergency adult patients with vertigo-related symptoms:<br>1. Focused examination<br>2. Execution and use of diagnostic examinations (auditory or advanced neuro-otology physical examination, including electrical nystagmus examination, inner ear temperature difference test, auditory brainstem response examination) and identification of the cause, making<br>3. Treatment or tracking plans, implementing necessary treatments (including vestibular rehabilitation),<br>4. Using system resource referrals<br>5. Health education and explanation after evaluation or treatment.<br>6. Medical record	Limitation: Only applicable to vertigo patients<br>Clinical situations necessary (not limited to) to complete training:<br>1. Cases of patients with acute vertigo attacks.<br>2. Cases of patients with repetitive vertigo attacks.<br>3. Cases of chronic dizziness patients<br>4. Include (not limited to) vestibular neuritis, benign paroxysmal vertigo, vestibular migraine, Meniere's disease, vertebrobasilar circulatory insufficiency, cervical vertigo, other central vertigo, etc. and their differential diagnosis

**3. Potential risks in case of failure**
The patient's diagnosis or treatment is improper due to the misinterpretation of hearing. Or inadequate or faulty diagnosis or treatment of dizziness in the ear. Patients may lose their good treatment opportunities and suffer from avoidable complications (such as falls). Errors in diagnosis or treatment lead to a waste of medical resources and loss of social trust in otorhinolaryngology and neck surgery.

4. Most relevant competency domains

**Patient Care**	**Medical Knowledge**	**Professionalism**	**Interpersonal & Communication Skills**	**Practice-based learning and improvement**	**System-based Practice**
✪✪<br><u>PC4</u><br>Otologic Disease	✪✪<br><u>MK3</u><br>Pathophysiolog-y<br>✪<br><u>MK1</u><br>Anatomy	✪✪<br><u>PROF2</u><br>Accountability/Conscientiousness<br>✪<br><u>PROF1</u><br>Professional Behavior and Ethical Principles<br><u>PROF3</u><br>Knowledge of Systemic and Individual Factors of Well-Being	✪✪<br><u>ICS1</u><br>Patient- and Family-Centered Communication<br>✪<br><u>ICS2</u><br>Interprofessional and Team Communication<br><u>ICS3</u><br>Communication within Health Care Systems	✪✪<br><u>PBLI1</u><br>Evidence-Based and Informed Practice<br>✪<br><u>PBLI2</u><br>Reflective practice and Commitment to Personal Growth	✪✪<br><u>SBP1</u><br>Patient Safety and Quality Improvement<br>✪<br><u>SBP2</u><br>System Navigation for Patient-Centered Care<br><u>SBP3</u><br>Physician Role in Health Care Systems

**5. Required knowledge, skills, attitudes and experiences to allow for summative entrustment**

**Knowledge:**	**Skills and Attitudes:**	**Required experience:**
1. Sufficient knowledge of vestibular cochlear nerve and temporal bone anatomy<br>2. Complete differential diagnosis of an ear-related vertigo disease.<br>3. The present invention can describe the diagnosis standard and natural history of benign paroxysmal positional vertigo (BPPV), vestibular neuritis, vaginitis, and Meniere's disease.<br>4. Non-operative treatment of positional vertigo, vaginitis, vestibular neuritis, and Meniere's disease<br>5. It can distinguish vestibular lesions and other non-earthy vertigo.<br>6. The present invention can describe the significant compensatory effect of peripheral vestibular lesions.	1. Stable execution of complete focus history interrogation and head and neck examination<br>2. Examining the ear using an earpiece helps to understand how to care for patient safety and comfort properly.<br>3. able to perform balance function check and basic neurology check (including cranial nerve check), identify and describe spontaneous, head-to-head and head-to-head change eye vibration to perform head, head shake, and Romberg tests skillfully<br>4. Medical communication skills such as explaining illness and informed consent<br>5. Team Cooperation Skills and Attitudes<br>6. patient-centered attitude	1. Essential core curriculum for ear, nose, and neck surgery

6. Information sources to assess progress and support summative entrustment

The EPA needs a blueprint to maintain the flexibility of the current training program's planning and to continue to build consensus. This version of EPAs recommends the planning of the assessment blueprint for the following six types of assessment tools (the CBME core group members consented to follow the principle of "at least two clinical teachers, at least two observations at different points of time," but not to that limited numbers of evaluations, multi-point, multi-oriented observations are the focus of EPAs assessment):

1. Written test (knowledge test): To design a valid knowledge test according to the connotation of the EPA task, and to confirm learners with required knowledge performing assessment and management of patients with airway presentations. Type of question included, but not limited to those testing understanding, analyzing, judgement, and application.
2. Simulation: To design a valid situational simulation test to describe the project's task and test the students' "situational ability."
3. Case-based discussion: To test learner's clinical thinking, reasoning, logic, attitude, etc., related to the EPA task. The recommended tools are CBD and EbD.
4. Short-practice observation (short-practice observation): Observe and evaluate student's performance in a task in the workplace. Recommended tools include DOPS, mini-CEX, EPAs, etc.
5. Long-practice observation and evaluation: Observe and evaluate the actual performance of trainees in the workplace during the previous period. This observation can avoid the "Hawthorne effect" of direct observation and evaluation in the workplace for a short period. It

is recommended to collect feedback from peers, colleagues, or patients through "multi-source feedback" to confirm student's performance in terms of accountability, communication, teamwork, and resilience.

6. Portfolio: To record the learning progress, including quantitative (for example, number of cases, number of operations) and qualitative (self-evaluation, experience, reflection) content, which can be used as a reference for accumulation of learning experience and self-learning ability. The recommended tools include medical certificate, clinic and inpatient medical records, surgical records, case log, journal reading, case-report, research paper publication.

7. Entrustment / supervision level expected at which stage of training

Schedule Entrustment-Supervision Level	Level 1	Level 2	Level 3	Level 4	Level 5
Before entering R2 training (R1)		✪			
Before entering R3 training (R2)			✪		
Before entering R4 training (R3)			✪		
Before entering R5 training (R4)				✪	
Before completing the training (R5)					✪

Level 1	the learner is allowed to be present and observe, not to enact an EPA
level 2	the learner is allowed to execute the EPA with direct, pro-active supervision, present in the room
level 3	the learner is allowed to carry out the EPA without a supervisor in the room, but quickly available if needed, i.e. with indirect, reactive, supervision
level 4	the learner is allowed to work unsupervised
level 5	the learner is allowed to provide supervision to more junior learners

8. Time period to expiration if not practiced

The entrustment / supervision level should be re-evaluated if relevant professional activities have not been implemented for 1 year.

Otorhinolaryngology–Head and Neck Surgery EPA05 (Infection)

**1. EPA Title**	
Assessing and managing patients with head and neck infections	
**2. Specification and limitations**	
The main complaint is symptoms associated with upper digestive tract or head and neck infection.<br>Clinic/Emergency Patient for Consultation: Focus-type diagnosis<br>1. Focus-type diagnosis<br>2. Use diagnostic tests to identify causes<br>3. Assessment of respiratory tract, if necessary<br>4. Choose the right antibiotics, set up treatment plans<br>5. Pre-operative Instructions and Operative Drainage and Clean-up<br>6. Postoperative wound care and complication treatment<br>7. Leverage system resource referrals<br>8. Health Education after Assessment or Disposal<br>9. Post-discharge medication and follow-up plan<br>10. Medical records	Limitation: Only applicable to exclude trauma patients<br>Clinical situations necessary (not limited to) to complete training:<br>1. Deep neck infection<br>2. Amygdala and Amygdala<br>3. Thyroid and salivary gland inflammation<br>4. Respiratory obstruction caused by infection<br>5. Mediastinum<br>6. Ear infection<br>7. Facial soft tissue infection.
**3. Potential risks in case of failure**	
1. If respiratory obstruction or delay in treatment leads to death<br>2. The infection leads to hyphema and another organ failure, which increases the consumption of medical resources and the financial burden on patients<br>3. Medical Disputes and Possible Pressure of Litigation	
**4. Most relevant competency domains**	

**Patient Care**	**Medical Knowledge**	**Professionalism**	**Interpersonal & Communication Skills**	**Practice-based learning and improvement**	**System-based Practice**
✪✪<br>PC1<br>Airway Emergency and Management<br>PC6<br>Laryngologic Disease<br>PC7<br>Pediatric Otolaryngology<br>✪<br>PC2<br>Foreign Body Management<br>PC3<br>Head and Neck	✪<br>MK1<br>Anatomy	✪✪<br>PROF2<br>Accountability/Conscientiousness<br>✪<br>PROF1<br>Professional Behavior and Ethical Principles<br>PROF3<br>Knowledge of Systemic and Individual Factors of Well-Being	✪✪<br>ICS1<br>Patient- and Family-Centered Communication<br>✪<br>ICS2<br>Interprofessional and Team Communication<br>ICS3<br>Communication within Health Care Systems	✪<br>PBLI1<br>Evidence-Based and Informed Practice<br>PBLI2<br>Reflective practice and Commitment to Personal Growth	✪✪<br>SBP1<br>Patient Safety and Quality Improvement<br>✪<br>SBP2<br>System Navigation for Patient-Centered Care<br>SBP3<br>Physician Role in Health Care Systems

Neoplasm<br>PC4<br>Otologic Disease<br>PC5<br>Rhinologic Disease<br>PC8<br>Facial Plastic and Reconstructive Surgery					

**5. Required knowledge, skills, attitudes and experiences to allow for summative entrustment**		
**Knowledge:**<br>1. Clinical manifestations and diagnosis of head and neck infections<br>2. Head and neck and upper digestive tract anatomy<br>3. Imaging Interpretation<br>4. Diagnosis and emergency treatment of respiratory obstruction<br>5. Antibiotic drug use<br>6. Indication of surgical drainage	**Skills and Attitude:**<br>1. Focus-based Medical History Examination of Head and Neck<br>2. Soft nasopharyngeal endoscopy<br>3. Airway establishment technology: Include surgical airway treatment<br>4. Infection site drainage and debridement includes a preoperative explanation and postoperative care.<br>5. Soft nasopharyngeal endoscopy<br>6. Explain medical conditions, informed consent, bad news (unexpected results), and other doctors<br>7. Disease communication skills<br>8. Team Attitude	**Required experience**:<br>1. Otorhinolaryngology Head and Neck Surgery Basic Core Course<br>2. Respiratory Tract Core Course

**6. Information sources to assess progress and support summative entrustment**
The EPA needs a blueprint to maintain the flexibility of the current training program's planning and to continue to build consensus. This version of EPAs recommends the planning of the assessment blueprint for the following six types of assessment tools (the CBME core group members consented to follow the principle of "at least two clinical teachers, at least two observations at different points of time," but not to that limited numbers of evaluations, multi-point, multi-oriented observations are the focus of EPAs assessment):<br>1. Written test (knowledge test): To design a valid knowledge test according to the connotation of the EPA task, and to confirm learners with required knowledge performing assessment and management of patients with airway presentations. Type of question included, but not limited to those testing understanding, analyzing, judgement, and application.<br>2. Simulation: To design a valid situational simulation test to describe the project's task and test the students' "situational ability."<br>3. Case-based discussion: To test learner's clinical thinking, reasoning, logic, attitude, etc., related to the EPA task. The recommended tools are CBD and EbD.

4. Short-practice observation (short-practice observation): Observe and evaluate student's performance in a task in the workplace. Recommended tools include DOPS, mini-CEX, EPAs, etc.
5. Long-practice observation and evaluation: Observe and evaluate the actual performance of trainees in the workplace during the previous period. This observation can avoid the "Hawthorne effect" of direct observation and evaluation in the workplace for a short period. It is recommended to collect feedback from peers, colleagues, or patients through "multi-source feedback" to confirm student's performance in terms of accountability, communication, teamwork, and resilience.
6. Portfolio: To record the learning progress, including quantitative (for example, number of cases, number of operations) and qualitative (self-evaluation, experience, reflection) content, which can be used as a reference for accumulation of learning experience and self-learning ability. The recommended tools include medical certificate, clinic and inpatient medical records, surgical records, case log, journal reading, case-report, research paper publication.

7. Entrustment / supervision level expected at which stage of training

Schedule Entrustment-Supervision Level	Level 1	Level 2	Level 3	Level 4	Level 5
Before entering R2 training (R1)		✪			
Before entering R3 training (R2)			✪		
Before entering R4 training (R3)			✪		
Before entering R5 training (R4)				✪	
Before completing the training (R5)					✪

Level 1	the learner is allowed to be present and observe, not to enact an EPA
level 2	the learner is allowed to execute the EPA with direct, pro-active supervision, present in the room
level 3	the learner is allowed to carry out the EPA without a supervisor in the room, but quickly available if needed, i.e. with indirect, reactive, supervision
level 4	the learner is allowed to work unsupervised
level 5	the learner is allowed to provide supervision to more junior learners

8. Time period to expiration if not practiced

The entrustment / supervision level should be re-evaluated if relevant professional activities have not been implemented for 1 year.

Otorhinolaryngology–Head and Neck Surgery EPA06 (H&N)

1. EPA Title

Assessing and managing patients with head and neck masses

2. Specification and limitations

The patient complained of a neck (including oral) mass to the clinic.<br>1. Focus-based diagnosis<br>2. Diagnostic tests and identification of causes<br>3. Schedule treatment and follow-up<br>4. Pre-operative instruction and assistance or execution of surgery<br>5. Postoperative care and treatment of complications<br>6. To collect staging results of patients with head and neck cancer, report and record discussion in a team meeting.<br>7. Leverage system resource referral<br>8. Hygiene and instruction after assessment or disposal<br>9. Medical records	Limitation: Only applicable to clinic and inpatients<br><br>Training must be completed.<br>1. Cases of cervical (including oral) tumor biopsy<br>2. Assisted completion of lymphatic or cervical mass surgery cases<br>3. A Case Study of Staging System for Lymphatic Metastasis (AJCC-TNM)<br>4. Complete Case Report for Diagnosis of Head and Neck Cancer<br>5. Participate in the head and neck cancer team meeting case discussion process, learn new ideas about diagnosis and treatment of cancer patients

3. Potential risks in case of failure

Cervical (including oral) neoplasms cause delayed diagnosis, misdiagnosis, treatment, and misdiagnosed complications of treatment, reduce treatment survival, or increase complications and quality of life after treatment.

4. Most relevant competency domains

**Patient Care**	**Medical Knowledge**	**Professionalism**	**Interpersonal & Communication Skills**	**Practice-based learning and improvement**	**System-based Practice**
✪✪<br>PC3<br>Head and Neck Neoplasm<br>✪<br>PC1<br>Airway Emergency and Management<br>PC4<br>Otologic Disease<br>PC5<br>Rhinologic Disease<br>PC6<br>Laryngologic Disease<br>PC8<br>Facial Plastic and Reconstructive Surgery	✪✪<br>MK1<br>Anatomy<br>MK3<br>Pathophysiology	✪✪<br>PROF2<br>Accountability/ Conscientiousness<br>✪<br>PROF1<br>Professional Behavior and Ethical Principles<br>PROF3<br>Knowledge of Systemic and Individual Factors of Well-Being	✪✪<br>ICS1<br>Patient- and Family-Centered Communication<br>ICS2<br>Interprofessional and Team Communication<br>ICS3<br>Communication within Health Care Systems	✪✪<br>PBLI1<br>Evidence-Based and Informed Practice<br>✪<br>PBLI2<br>Reflective practice and Commitment to Personal Growth	✪✪<br>SBP1<br>Patient Safety and Quality Improvement<br>SBP2<br>System Navigation for Patient-Centered Care<br>SBP3<br>Physician Role in Health Care Systems

5. Required knowledge, skills, attitudes and experiences to allow for summative entrustment		
**Knowledge:**<br>1. Cervical embryo development<br>2. Epidemiology of cervical cancer in Taiwan<br>3. Common diseases of neck masses<br>4. TNM staging of cervical neoplasms<br>5. treatment of common neck tumor<br>6. Common complications in the treatment of cervical tumors	**Skills and Attitudes:**<br>1. Local findings<br>2. Lumps, including lymph node dissection<br>3. neck ultrasound<br>4. Neck ultrasonic fine needle puncture or thick needle section examination<br>5. Lymph or neck swelling surgery<br>6. Surgical complication management of lymph or neck tumor<br>7. Medical communication skills include interpreting symptoms, informed consent, and communicating bad news (death, unintended consequences).<br>8. Teamwork skills and attitudes: Include leadership, communication, situational and solidarity skills, building a team shared mental model, shared purpose, and respectful attitudes.	**Required experience**:<br>1. Basic core course of otolaryngology head and neck surgery

6. Information sources to assess progress and support summative entrustment

The EPA needs a blueprint to maintain the flexibility of the current training program's planning and to continue to build consensus. This version of EPAs recommends the planning of the assessment blueprint for the following six types of assessment tools (the CBME core group members consented to follow the principle of "at least two clinical teachers, at least two observations at different points of time," but not to that limited numbers of evaluations, multi-point, multi-oriented observations are the focus of EPAs assessment):

1. Written test (knowledge test): To design a valid knowledge test according to the connotation of the EPA task, and to confirm learners with required knowledge performing assessment and management of patients with airway presentations. Type of question included, but not limited to those testing understanding, analyzing, judgement, and application.
2. Simulation: To design a valid situational simulation test to describe the project's task and test the students' "situational ability."
3. Case-based discussion: To test learner's clinical thinking, reasoning, logic, attitude, etc., related to the EPA task. The recommended tools are CBD and EbD.
4. Short-practice observation (short-practice observation): Observe and evaluate student's performance in a task in the workplace. Recommended tools include DOPS, mini-CEX, EPAs, etc.
5. Long-practice observation and evaluation: Observe and evaluate the actual performance of trainees in the workplace during the previous period. This observation can avoid the "Hawthorne effect" of direct observation and evaluation in the workplace for a short period. It is recommended to collect feedback from peers, colleagues, or patients through "multi-source

feedback" to confirm student's performance in terms of accountability, communication, teamwork, and resilience.

6. Portfolio: To record the learning progress, including quantitative (for example, number of cases, number of operations) and qualitative (self-evaluation, experience, reflection) content, which can be used as a reference for accumulation of learning experience and self-learning ability. The recommended tools include medical certificate, clinic and inpatient medical records, surgical records, case log, journal reading, case-report, research paper publication.

7. Entrustment / supervision level expected at which stage of training

Schedule / Entrustment-Supervision Level	Level 1	Level 2	Level 3	Level 4	Level 5
Before entering R2 training (R1)		✪			
Before entering R3 training (R2)		✪			
Before entering R4 training (R3)			✪		
Before entering R5 training (R4)				✪	
Before completing the training (R5)					✪

Level 1	the learner is allowed to be present and observe, not to enact an EPA
level 2	the learner is allowed to execute the EPA with direct, pro-active supervision, present in the room
level 3	the learner is allowed to carry out the EPA without a supervisor in the room, but quickly available if needed, i.e. with indirect, reactive, supervision
level 4	the learner is allowed to work unsupervised
level 5	the learner is allowed to provide supervision to more junior learners

8. Time period to expiration if not practiced

The entrustment / supervision level should be re-evaluated if relevant professional activities have not been implemented for 1 year.

Otorhinolaryngology–Head and Neck Surgery EPA07 (Ear)

**1. EPA Title**
Assessing and managing patients with ear and hearing diseases

**2. Specification and limitations**	
For common ear and hearing diseases, it has the ability of diagnosis and treatment, including:<br><br>1. Focus-based diagnosis<br>2. Perform and apply diagnostic tests and identify causes<br>3. Prescribe treatment or follow-up plan, perform necessary disposal<br>4. Assist or perform pediatric and adult otitis media surgery<br>5. Hygiene and instruction after assessment or disposal<br>6. Leverage system resource referral<br>7. Medical records	Limitation: Only applicable to conscious vital signs stabilize patient's ear and hearing the assessment and the disposal<br><br>Clinical situations necessary (not limited to) to complete training:<br>1. Diagnosis and treatment of patients with sudden deafness<br>2. Diagnosis and treatment of patients with acute low-frequency hearing loss<br>3. Diagnosis and Treatment of Tinnitus and Noisy Hearing Impairment<br>4. Diagnosis and Treatment of Otitis Media in Children and Adults<br>5. Diagnosis and surgical cases of chronic otitis media

**3. Potential risks in case of failure**
Misdiagnosis or mishandling of patients due to hearing interpretation, the ear disease diagnosis insufficiency, or the unjust treatment and the mistake, causes the patient to lose the right treatment opportunity, suffer initially and avoid complications (such as infection, hearing damage, etc.). The differential diagnosis or the treatment mistake causes medical resources waste, the society to the ear nose throat head and neck surgery profession trust loss and so on.

4. Knowledge, Skills and Attitude, and Experience required

**Patient Care**	**Medical Knowledge**	**Professionalism**	**Interpersonal & Communication Skills**	**Practice-based learning and improvement**	**System-based Practice**
✪✪<br>PC4<br>Otologic Disease<br>✪<br>PC2<br>Foreign Body Management<br>PC3<br>Head and Neck Neoplasm<br>PC7<br>Pediatric Otolaryngology<br>PC8<br>Facial Plastic and Reconstructive Surgery	✪✪<br>MK1<br>Anatomy<br>MK3<br>Pathophysiology	✪✪<br>PROF2<br>Accountability/Conscientiousness<br>✪<br>PROF1<br>Professional Behavior and Ethical Principles<br>PROF3<br>Knowledge of Systemic and Individual Factors of Well-Being	✪✪<br>ICS1<br>Patient- and Family-Centered Communication<br>✪<br>ICS2<br>Interprofessional and Team Communication<br>ICS3<br>Communication within Health Care Systems	✪<br>PBLI1<br>Evidence-Based and Informed Practice<br>PBLI2<br>Reflective practice and Commitment to Personal Growth	✪✪<br>SBP1<br>Patient Safety and Quality Improvement<br>✪<br>SBP2<br>System Navigation for Patient-Centered Care<br>SBP3<br>Physician Role in Health Care Systems

5. **Required knowledge, skills, attitudes and experiences to allow for summative entrustment**		
**Knowledge:**<br>1. Be familiar with the knowledge of temporal bone, cochlear vestibular anatomy, and embryology.<br>2. To understand the mechanism of middle ear sound transfer and cochlear physiology.<br>3. Understanding the causes and natural processes of hearing loss in children and adults<br>4. Understand the central auditory pathway.<br>5. Understand otitis media, otitis externa and ear infections.<br>6. Understand chronic ear disorders.<br>7. Understanding the internal and surgical treatment of ear disorders<br>8. To identify the physiological and pathological causes of otorhinolaryngeal dysfunction.	**Skills and Attitude:**<br>1. It can stably execute complete focus and neck examination.<br>2. Can use the ear lens examination, can understand how to pay appropriate attention to patient safety and comfort;<br>3. Can perform hearing tests and readings.<br>4. It is capable of performing advanced auditory neurological examinations such as auditory brainstem response, electrical ophthalmogram, and temperature difference test of the inner ear<br>5. Common ear surgery can be performed, including middle ear ventilation and tracheal surgery, tympanoplasty surgery, mastoid surgery, and so on.<br>6. Explain medical communication skills such as illness and informed consent.<br>7. Teamwork skills and attitudes<br>8. Patient-centered attitude	**Required experience**:<br>1. Basic core courses of ENT Head and Neck Surgery

6. **Information sources to assess progress and support summative entrustment**
The EPA needs a blueprint to maintain the flexibility of the current training program’s planning and to continue to build consensus. This version of EPAs recommends the planning of the assessment blueprint for the following six types of assessment tools (the CBME core group members consented to follow the principle of “at least two clinical teachers, at least two observations at different points of time,” but not to that limited numbers of evaluations, multi-point, multi-oriented observations are the focus of EPAs assessment):<br>1. Written test (knowledge test): To design a valid knowledge test according to the connotation of the EPA task, and to confirm learners with required knowledge performing assessment and management of patients with airway presentations. Type of question included, but not limited to those testing understanding, analyzing, judgement, and application.<br>2. Simulation: To design a valid situational simulation test to describe the project's task and test the students' "situational ability."<br>3. Case-based discussion: To test learner's clinical thinking, reasoning, logic, attitude, etc., related to the EPA task. The recommended tools are CBD and EbD.<br>4. Short-practice observation (short-practice observation): Observe and evaluate student's performance in a task in the workplace. Recommended tools include DOPS, mini-CEX, EPAs, etc.<br>5. Long-practice observation and evaluation: Observe and evaluate the actual performance of trainees in the workplace during the previous period. This observation can avoid the "Hawthorne effect" of direct observation and evaluation in the workplace for a short period. It

is recommended to collect feedback from peers, colleagues, or patients through "multi-source feedback" to confirm student's performance in terms of accountability, communication, teamwork, and resilience.

6. Portfolio: To record the learning progress, including quantitative (for example, number of cases, number of operations) and qualitative (self-evaluation, experience, reflection) content, which can be used as a reference for accumulation of learning experience and self-learning ability. The recommended tools include medical certificate, clinic and inpatient medical records, surgical records, case log, journal reading, case-report, research paper publication.

7. Entrustment / supervision level expected at which stage of training

Schedule Entrustment-Supervision Level	Level 1	Level 2	Level 3	Level 4	Level 5
Before entering R2 training (R1)		✪			
Before entering R3 training (R2)			✪		
Before entering R4 training (R3)			✪		
Before entering R5 training (R4)				✪	
Before completing the training (R5)					✪

Level 1	the learner is allowed to be present and observe, not to enact an EPA
level 2	the learner is allowed to execute the EPA with direct, pro-active supervision, present in the room
level 3	the learner is allowed to carry out the EPA without a supervisor in the room, but quickly available if needed, i.e. with indirect, reactive, supervision
level 4	the learner is allowed to work unsupervised
level 5	the learner is allowed to provide supervision to more junior learners

8. Time period to expiration if not practiced

The entrustment / supervision level should be re-evaluated if relevant professional activities have not been implemented for 1 year.

Otorhinolaryngology–Head and Neck Surgery EPA08 (Sinonasal)

<table>
<tr><td colspan="6">1. EPA Title</td></tr>
<tr><td colspan="6">Assessing and managing patients with sinonasal diseases</td></tr>
<tr><td colspan="6">2. Specification and limitations</td></tr>
<tr><td colspan="3">Clinic or inpatients with nasal and sinus diseases:
1. Focus-based diagnosis and etiology identification
2. Use Endoscope and Image Inspection to Determine Diagnosis
3. Develop medication
4. Determine that need for surgical treatment
5. Preoperative instructions and perform nose/sinus surgery
6. Hygiene and instruction after assessment or disposal
7. Postoperative wound care and follow-up treatment plan
8. Medical records</td><td colspan="3">Limitation: Only applicable for adults

Clinical situations necessary (not limited to) to complete training:
1. Perform inferior turbinate, nasal septum, and sinus surgery cases
2. Nose Treatment of complications after surgery (observation)</td></tr>
<tr><td colspan="6">3. Potential risks in case of failure</td></tr>
<tr><td colspan="6">1. Misdiagnosis leads to inadequate treatment and even loss of trust
2. Inadequate treatment leads to poor outcomes and even to risks of medical errors and disputes</td></tr>
<tr><td colspan="6">4. Most relevant competency domains</td></tr>
<tr><td>Patient Care
✪✪
<u>PC5</u>
Rhinologi-c Disease
✪
<u>PC2</u>
Foreign Body Managem ent
<u>PC3</u>
Head and Neck Neoplasm
<u>PC7</u>
Pediatric Otolaryng ology</td><td>Medical Knowledge
✪✪
<u>MK1</u>
Anatomy
<u>MK2</u>
Allergy
<u>MK3</u>
Pathophysiolo gy</td><td>Professionalism
✪✪
<u>PROF2</u>
Accountability/Con scientiousness
✪
<u>PROF1</u>
Professional Behavior and Ethical Principles
<u>PROF3</u>
Knowledge of Systemic and Individual Factors of Well-Being</td><td>Interpersonal & Communication Skills
✪✪
<u>ICS1</u>
Patient- and Family-Centered Communication
✪
<u>ICS2</u>
Interprofessional and Team Communication
<u>ICS3</u>
Communication within Health Care Systems</td><td>Practice-based learning and improvement
✪
<u>PBLI1</u>
Evidence-Based and Informed Practice
<u>PBLI2</u>
Reflective practice and Commitment to Personal Growth</td><td>System-based Practice
✪✪
<u>SBP1</u>
Patient Safety and Quality Improvement
✪
<u>SBP2</u>
System Navigation for Patient-Centered Care
<u>SBP3</u>
Physician Role in Health Care Systems</td></tr>
</table>

PC8 Facial Plastic and Reconstructive Surgery<br>PC9 Sleep					

5. Required knowledge, skills, attitudes and experiences to allow for summative entrustment		
**Knowledge:**<br>1. Clinical manifestations and diagnostic basis of various rhinitis include allergic rhinitis, hypertrophic rhinitis, vasomotor rhinitis, and drug-induced rhinitis.<br>2. Clinical manifestation and diagnostic basis of various rhinosinusitis, including rhinosinusitis with nasal polyps, bacterial sinusitis, mycotic sinusitis, and odontogenic sinusitis.<br>3. Upper and lower respiratory allergy rotation and the association between the two.<br>4. Olfactory abnormalities are common causes and handling.<br>5. Clinical manifestations and diagnostic basis of common nasal tumors, including inverted mastoid tumor, olfactory mother cell tumor, squamous epithelium, and cell carcinoma.<br>6. Signs, doses, and adverse effects of various nasal medications, including antihistamines, antibiotics, oral	**Skills and Attitudes:**<br>1. Focus-based diagnosis<br>2. Physical Examination of Nasal Cavity<br>3. Interpretation of nasal imaging examination<br>4. Operation of nasal endoscope<br>5. Inferior turbinate surgery<br>6. Nasal septal surgery<br>7. Endoscopic sinus surgery<br>8. Detect and Act on Complications<br>9. Explaining medical communication skills such as medical condition and informed consent<br>10. Patient-centered attitude	**Necessary experience**:<br>1. The basic core course of otolaryngology head and neck surgery<br>2. Endoscopy, sinus, and skull base Hand-on dissection course

steroids, and steroid nasal sprays.<br>7. Indications and surgical methods requiring surgical intervention include inferior turbinate surgery, nasal medial phrenic surgery, and endoscopic sinus surgery.<br>8. The postoperative treatment plan, detection, and treatment of intraoperative/postoperative complications of endoscopic sinus surgery.		

6. Information sources to assess progress and support summative entrustment

The EPA needs a blueprint to maintain the flexibility of the current training program's planning and to continue to build consensus. This version of EPAs recommends the planning of the assessment blueprint for the following six types of assessment tools (the CBME core group members consented to follow the principle of "at least two clinical teachers, at least two observations at different points of time," but not to that limited numbers of evaluations, multi-point, multi-oriented observations are the focus of EPAs assessment):

1. Written test (knowledge test): To design a valid knowledge test according to the connotation of the EPA task, and to confirm learners with required knowledge performing assessment and management of patients with airway presentations. Type of question included, but not limited to those testing understanding, analyzing, judgement, and application.
2. Simulation: To design a valid situational simulation test to describe the project's task and test the students' "situational ability."
3. Case-based discussion: To test learner's clinical thinking, reasoning, logic, attitude, etc., related to the EPA task. The recommended tools are CBD and EbD.
4. Short-practice observation (short-practice observation): Observe and evaluate student's performance in a task in the workplace. Recommended tools include DOPS, mini-CEX, EPAs, etc.
5. Long-practice observation and evaluation: Observe and evaluate the actual performance of trainees in the workplace during the previous period. This observation can avoid the "Hawthorne effect" of direct observation and evaluation in the workplace for a short period. It is recommended to collect feedback from peers, colleagues, or patients through "multi-source feedback" to confirm student's performance in terms of accountability, communication, teamwork, and resilience.
6. Portfolio: To record the learning progress, including quantitative (for example, number of cases, number of operations) and qualitative (self-evaluation, experience, reflection) content, which can be used as a reference for accumulation of learning experience and self-learning ability. The recommended tools include medical certificate, clinic and inpatient medical records, surgical records, case log, journal reading, case-report, research paper publication.

7. Entrustment / supervision level expected at which stage of training

Schedule Entrustment-Supervision Level	Level 1	Level 2	Level 3	Level 4	Level 5
Before entering R2 training (R1)		✪			
Before entering R3 training (R2)			✪		
Before entering R4 training (R3)			✪		
Before entering R5 training (R4)				✪	
Before completing the training (R5)					✪

Level 1	the learner is allowed to be present and observe, not to enact an EPA
level 2	the learner is allowed to execute the EPA with direct, pro-active supervision, present in the room
level 3	the learner is allowed to carry out the EPA without a supervisor in the room, but quickly available if needed, i.e. with indirect, reactive, supervision
level 4	the learner is allowed to work unsupervised
level 5	the learner is allowed to provide supervision to more junior learners

8. Time period to expiration if not practiced
The entrustment / supervision level should be re-evaluated if relevant professional activities have not been implemented for 1 year.

Otorhinolaryngology–Head and Neck Surgery EPA09 (Larynx)

<table>
<tr><td colspan="2">1. EPA Title</td></tr>
<tr><td colspan="2">Assessing and managing patients with laryngopharyngeal diseases (voice/speech/language/dysphagia)</td></tr>
<tr><td colspan="2">2. Specification and limitations</td></tr>
<tr><td rowspan="2">For clinic/emergency patients with symptoms related to voice, speech, and swallowing disorders:
1. Focused examination
2. Use of diagnostic tests to differentiate causes and attention to potential underlying cancer and central nervous system diseases
3. Establishment of a treatment plan
4. Preoperative explanation
5. Performing vocal cord/pharyngeal surgery
6. Postoperative wound care
7. Management of complications
8. Health education and explanation after assessment or treatment
9. Utilization of system resource referrals
10. Medical records</td><td>Limitation:: No need to</td></tr>
<tr><td>Clinical situations necessary (not limited to) to complete training:
1. Benign vocal cord lesions (nodules, polyps, and cysts)
2. Unilateral and Bilateral Vocal Cord Paralysis (including Recurrent Laryngeal Nerve Injury and Compression Caused by Associated Cancer)
3. Abnormal sound formation in children
4. Old People's Voice and Dysphagia
5. Central nervous disorders combined with dysphagia, language disorders
6. Throat cancer</td></tr>
<tr><td colspan="2">3. Potential risks in case of failure</td></tr>
<tr><td colspan="2">1. Untreated voice problems may affect the patient’s daily routine Work, resulting in a significant decline in the quality of life.
2. Some patients may therefore affect their studies or social activities, and cause withdrawal behavior.
3. If aspiration pneumonia is caused, it will increase the consumption of medical resources. In severe cases, it may also lead to the death of the patient.
4. No possible potential tumors have been found. , delay in treatment will lead to disease progression, prognosis impact</td></tr>
<tr><td colspan="2">4. Most relevant competency domains</td></tr>
</table>

**Patient Care**	**Medical Knowledge**	**Professionalism**	**Interpersonal & Communication Skills**	**Practice-based learning and improvement**	**System-based Practice**
✪✪<br>PC6<br>Laryngologic Disease<br>✪<br>PC1<br>Airway Emergency and Management<br>PC2<br>Foreign Body Management<br>PC3<br>Head and Neck Neoplasm<br>PC7	✪✪<br>MK1<br>Anatomy<br>✪<br>MK2<br>Allergy<br>MK3<br>Pathophysiology	✪✪<br>PROF2<br>Accountability/ Conscientiousness<br>✪<br>PROF1<br>Professional Behavior and Ethical Principles<br>PROF3<br>Knowledge of Systemic and Individual	✪✪<br>ICS1<br>Patient- and Family-Centered Communication<br>✪<br>ICS2<br>Interprofessional and Team Communication<br>ICS3<br>Communication within Health Care Systems	✪<br>PBLI1<br>Evidence-Based and Informed Practice<br>PBLI2<br>Reflective practice and Commitment to Personal Growth	✪✪<br>SBP1<br>Patient Safety and Quality Improvement<br>✪<br>SBP2<br>System Navigation for Patient-Centered Care<br>SBP3<br>Physician Role in Health Care Systems

Pediatric Otolaryngology<br>PC8<br>Facial Plastic and Reconstructive Surgery<br>PC9<br>Sleep		Factors of Well-Being			

5. Required knowledge, skills, attitudes and experiences to allow for summative entrustment		
**Knowledge:**<br>1. Clinical Manifestations and Diagnosis of Abnormal Voice and Dysphagia<br>2. Anatomy, Neurology of Upper Digestive Tract<br>3. Manifestation of Central Nervous Disease in Throat<br>4. Interpretation of Imaging Inspection<br>5. Language, Swallowing, and Surgical Treatment of Adaptation	**Skills and Attitude:**<br>1. Focus Medical History Inquiry<br>2. Distinguishing abnormal voice<br>3. Head and neck body diagnosis<br>4. Soft nasopharynx endoscopy<br>5. Develop a treatment or rehabilitation plan<br>6. Nursing and Nursing Care before, during, and after Operation<br>7. Team cooperation	**Required Experience**:<br>1. Otorhinolaryngology Head and Neck Surgery Basic Core Course<br>2. Respiratory Tract Core Course

6. Information sources to assess progress and support summative entrustment
The EPA needs a blueprint to maintain the flexibility of the current training program's planning and to continue to build consensus. This version of EPAs recommends the planning of the assessment blueprint for the following six types of assessment tools (the CBME core group members consented to follow the principle of "at least two clinical teachers, at least two observations at different points of time," but not to that limited numbers of evaluations, multi-point, multi-oriented observations are the focus of EPAs assessment):<br>1. Written test (knowledge test): To design a valid knowledge test according to the connotation of the EPA task, and to confirm learners with required knowledge performing assessment and management of patients with airway presentations. Type of question included, but not limited to those testing understanding, analyzing, judgement, and application.<br>2. Simulation: To design a valid situational simulation test to describe the project's task and test the students' "situational ability."<br>3. Case-based discussion: To test learner's clinical thinking, reasoning, logic, attitude, etc., related to the EPA task. The recommended tools are CBD and EbD.<br>4. Short-practice observation (short-practice observation): Observe and evaluate student's performance in a task in the workplace. Recommended tools include DOPS, mini-CEX, EPAs, etc.<br>5. Long-practice observation and evaluation: Observe and evaluate the actual performance of trainees in the workplace during the previous period. This observation can avoid the "Hawthorne effect" of direct observation and evaluation in the workplace for a short period. It is recommended to collect feedback from peers, colleagues, or patients through "multi-source feedback" to confirm student's performance in terms of accountability, communication, teamwork, and resilience.

6. Portfolio: To record the learning progress, including quantitative (for example, number of cases, number of operations) and qualitative (self-evaluation, experience, reflection) content, which can be used as a reference for accumulation of learning experience and self-learning ability. The recommended tools include medical certificate, clinic and inpatient medical records, surgical records, case log, journal reading, case-report, research paper publication.

7. Entrustment / supervision level expected at which stage of training

Schedule / Entrustment-Supervision Level	Level 1	Level 2	Level 3	Level 4	Level 5
Before entering R2 training (R1)		✪			
Before entering R3 training (R2)			✪		
Before entering R4 training (R3)			✪		
Before entering R5 training (R4)				✪	
Before completing the training (R5)					✪

Level 1	the learner is allowed to be present and observe, not to enact an EPA
level 2	the learner is allowed to execute the EPA with direct, pro-active supervision, present in the room
level 3	the learner is allowed to carry out the EPA without a supervisor in the room, but quickly available if needed, i.e. with indirect, reactive, supervision
level 4	the learner is allowed to work unsupervised
level 5	the learner is allowed to provide supervision to more junior learners

8. Time period to expiration if not practiced

The entrustment / supervision level should be re-evaluated if relevant professional activities have not been implemented for 1 year.

Otorhinolaryngology–Head and Neck Surgery EPA10 (SDB)

<table>
<tr><td colspan="6">1. EPA Title</td></tr>
<tr><td colspan="6">Assessing and managing patients with sleep-disordered breathing</td></tr>
<tr><td colspan="6">2. Specification and limitations</td></tr>
<tr><td colspan="3" rowspan="2">Clinic/inpatient patients (adults and children) with snoring or suspected obstructive sleep apnea-related symptoms as the primary complaint:

1. Focus-type diagnosis
2. Use diagnostic tests to identify causes
3. Setting up treatment and tracking programs
4. Preoperative Instructions
5. Perform nasal/palate/throat/laryngopharynx surgery
6. Postoperative care and complications
7. Health Education after Assessment or Disposal
8. Leverage system resource referrals
9. Medical records</td><td colspan="3">Limitation: Only applicable for sleep breathing disorder patients, and can exclude simple nose or throat diseases and sleep breathing disorder patients.</td></tr>
<tr><td colspan="3">Clinical situations necessary (not limited to) to complete training:
1. The Case of Endoscopic Examination of Nasopharynx Fibers (Awake/Induced Sleep)
2. Case Study on Diagnosis and Explanation of Sleep Respiration Physiological Examination Report
3. Case of Performing Palatal/Pharyngeal Tonsils/Soft Palate/Tongue Surgery
4. Emergency treatment of postoperative hemorrhage complications</td></tr>
<tr><td colspan="6">3. Potential risks in case of failure</td></tr>
<tr><td colspan="6">Patients may suffer from chronic diseases, such as hypertension, cardiovascular/cerebrovascular diseases, metabolic syndrome, traffic accidents, or even sudden death, medical resources wasted due to identification errors, disability, or death due to improper treatment of complications. Medical teams face disputes or litigation pressure and loss of social trust in otolaryngology, head, and neck surgery.</td></tr>
<tr><td colspan="6">4. Most relevant competency domains</td></tr>
<tr><td>Patient Care
✪✪
<u>PC7</u>
Pediatric Otolaryngology
<u>PC9</u>
Sleep
✪
<u>PC5</u>
Rhinologic Disease
<u>PC6</u>
Laryngologic Disease</td><td>Medical Knowledge
✪
<u>MK1</u>
Anatomy
<u>MK2</u>
Allergy
<u>MK3</u>
Pathophysiology</td><td>Professionalism
✪✪
<u>PROF2</u>
Accountability/Conscientiousness
✪
<u>PROF1</u>
Professional Behavior and Ethical Principles
<u>PROF3</u>
Knowledge of Systemic and Individual Factors of Well-Being</td><td>Interpersonal & Communication Skills
✪✪
<u>ICS1</u>
Patient- and Family-Centered Communication
<u>ICS2</u>
Interprofessional and Team Communication)
✪
<u>ICS3</u>
Communication within Health Care Systems</td><td>Practice-based learning and improvement
✪
<u>PBLI1</u>
Evidence-Based and Informed Practice
<u>PBLI2</u>
Reflective practice and Commitment to Personal Growth</td><td>System-based Practice
✪✪
<u>SBP1</u>
Patient Safety and Quality Improvement
<u>SBP2</u>
System Navigation for Patient-Centered Care
<u>SBP3</u>
Physician Role in Health Care Systems</td></tr>
</table>

5. **Required knowledge, skills, attitudes and experiences to allow for summative entrustment**		
**Knowledge:**<br>1. Anatomy of the upper respiratory and digestive tract<br>2. Breathe to body<br>3. Interpretation of Sleep Respiratory Physiological Examination Report<br>4. Sleep Apnea in Adults and Children<br>5. Internal/surgical/alternative treatment<br>6. Complications and methods of treatment<br>7. List of treatment and tracking plans	**Skills and Attitudes:**<br>1. Focus Medical History Inquiry and Medical Diagnosis<br>2. Endoscopic examination of nasopharynx fibers<br>3. Schedule treatment for typical patients<br>4. Nursing and Nursing Care before, during, and after Operation<br>5. Performing nasal/palate and pharyngeal tonsils/soft palate surgery<br>6. Detect complications and dispose of them immediately<br>7. Using Resources to Transfer Timely<br>8. Medical communication skills such as explaining illness, informed consent, and informing bad news (non-expected result)<br>9. Team Cooperation Skills and Attitudes<br>10. Patient-centered attitude	**Required Experience**:<br>1. Essential core curriculum for ear, nose and neck surgery<br>2. Respiratory core curriculum

6. **Information sources to assess progress and support summative entrustment**
The EPA needs a blueprint to maintain the flexibility of the current training program's planning and to continue to build consensus. This version of EPAs recommends the planning of the assessment blueprint for the following six types of assessment tools (the CBME core group members consented to follow the principle of "at least two clinical teachers, at least two observations at different points of time," but not to that limited numbers of evaluations, multi-point, multi-oriented observations are the focus of EPAs assessment):<br>1. Written test (knowledge test): To design a valid knowledge test according to the connotation of the EPA task, and to confirm learners with required knowledge performing assessment and management of patients with airway presentations. Type of question included, but not limited to those testing understanding, analyzing, judgement, and application.<br>2. Simulation: To design a valid situational simulation test to describe the project's task and test the students' "situational ability."<br>3. Case-based discussion: To test learner's clinical thinking, reasoning, logic, attitude, etc., related to the EPA task. The recommended tools are CBD and EbD.<br>4. Short-practice observation (short-practice observation): Observe and evaluate student's performance in a task in the workplace. Recommended tools include DOPS, mini-CEX, EPAs, etc.<br>5. Long-practice observation and evaluation: Observe and evaluate the actual performance of trainees in the workplace during the previous period. This observation can avoid the "Hawthorne effect" of direct observation and evaluation in the workplace for a short period. It is recommended to collect feedback from peers, colleagues, or patients through "multi-source feedback" to confirm student's performance in terms of accountability, communication, teamwork, and resilience.<br>6. Portfolio: To record the learning progress, including quantitative (for example, number of cases, number of operations) and qualitative (self-evaluation, experience, reflection) content, which can be used as a reference for accumulation of learning experience and self-learning

ability. The recommended tools include medical certificate, clinic and inpatient medical records, surgical records, case log, journal reading, case-report, research paper publication.

7. Entrustment / supervision level expected at which stage of training

Schedule Entrustment-Supervision Level	Level 1	Level 2	Level 3	Level 4	Level 5
Before entering R2 training (R1)		✪			
Before entering R3 training (R2)		✪			
Before entering R4 training (R3)			✪		
Before entering R5 training (R4)				✪	
Before completing the training (R5)					✪

Level 1	the learner is allowed to be present and observe, not to enact an EPA
level 2	the learner is allowed to execute the EPA with direct, pro-active supervision, present in the room
level 3	the learner is allowed to carry out the EPA without a supervisor in the room, but quickly available if needed, i.e. with indirect, reactive, supervision
level 4	the learner is allowed to work unsupervised
level 5	the learner is allowed to provide supervision to more junior learners

8. Time period to expiration if not practiced

The entrustment / supervision level should be re-evaluated if relevant professional activities have not been implemented for 1 year.

Otorhinolaryngology–Head and Neck Surgery EPA11 (Plasty)

1. EPA Title

Assessing and managing patients with facial plastic and reconstructive surgery

2. Specification and limitations

Reconstruction of patients with facial reconstruction:
1. Diagnosis and Collection of Medical History
2. face analysis
3. image recording
4. Schedule objective checks
5. Medication
6. Assistance or surgical treatment
7. Postoperative wound care
8. Communication with patients and family members
9. Health Education after Assessment or Disposal
10. Medical records

Limitation: For adults only

Clinical situations necessary (not limited to) to complete training:
1. Evaluation and treatment of facial trauma
2. Repair and reconstruction after tumor resection
3. Functional repair of upper respiratory obstruction
4. Treatment of Beauty Requirements and Operation

3. Potential risks in case of failure

1. Physical and psychological harm caused by wrong diagnosis or improper treatment
2. The loss of confidence in doctors can lead to future burnout.
3. The conflict and dispute between the two sides have caused mental and financial loss.

4. Most relevant competency domains

Patient Care	Medical Knowledge	Professionalism	Interpersonal & Communication Skills	Practice-based learning and improvement	System-based Practice
✪✪<br>PC8<br>Facial Plastic and Reconstructive Surgery<br>✪<br>PC3<br>Head and Neck Neoplasm<br>PC5<br>Rhinologic Disease	✪<br>MK1<br>Anatomy	✪✪<br>PROF2<br>Accountability/Conscientiousness<br>✪<br>PROF1<br>Professional Behavior and Ethical Principles<br>PROF3<br>Knowledge of Systemic and Individual Factors of Well-Being	✪✪<br>ICS1<br>Patient- and Family-Centered Communication<br>✪<br>ICS2<br>Interprofessional and Team Communication<br>ICS3<br>Communication within Health Care Systems	✪<br>PBLI1<br>Evidence-Based and Informed Practice<br>PBLI2<br>Reflective practice and Commitment to Personal Growth	✪✪<br>SBP1<br>Patient Safety and Quality Improvement<br>✪<br>SBP2<br>System Navigation for Patient-Centered Care<br>SBP3<br>Physician Role in Health Care Systems

5. Required knowledge, skills, attitudes and experiences to allow for summative entrustment		
**Knowledge:**<br>1. Various facial anatomy and physiological knowledge<br>2. Familiarity with various facial structural deformations and physiological disorders<br>3. Respiratory tract allergies, rhinitis, and sinusitis are associated with facial structural deformations and physiological disorders.<br>4. Treatment of Various Facial Micro-Texturing<br>5. A variety of facial plastic surgery.<br>6. The familiarity with the individual cartilage and artificial material needed for facial treatment and surgery.<br>7. The long-term effects of various facial treatments.<br>8. Various facial treatments and surgical complications.	**Skills and Attitudes:**<br>1. Complete medical history<br>2. The Body Diagnosis of Face<br>3. Face analysis<br>4. Interpretation of Facial Image Examination<br>5. Operation of nasal endoscope<br>6. The precision of Facial Image Recording<br>7. Face Micro-Shaping and Treatment<br>8. Facial surgery<br>9. Medical communication skills such as explaining illness and informed consent<br>10. Patient-centered attitude	**Required Experience**:<br><br>1. Essential core curriculum for ear, nose, and neck surgery<br>2. The Basic Core Course of Face Reconstruction<br>3. Operation Practice of Face Reconstruction

6. Information sources to assess progress and support summative entrustment

The EPA needs a blueprint to maintain the flexibility of the current training program's planning and to continue to build consensus. This version of EPAs recommends the planning of the assessment blueprint for the following six types of assessment tools (the CBME core group members consented to follow the principle of "at least two clinical teachers, at least two observations at different points of time," but not to that limited numbers of evaluations, multi-point, multi-oriented observations are the focus of EPAs assessment):

1. Written test (knowledge test): To design a valid knowledge test according to the connotation of the EPA task, and to confirm learners with required knowledge performing assessment and management of patients with airway presentations. Type of question included, but not limited to those testing understanding, analyzing, judgement, and application.
2. Simulation: To design a valid situational simulation test to describe the project's task and test the students' "situational ability."
3. Case-based discussion: To test learner's clinical thinking, reasoning, logic, attitude, etc., related to the EPA task. The recommended tools are CBD and EbD.
4. Short-practice observation (short-practice observation): Observe and evaluate student's performance in a task in the workplace. Recommended tools include DOPS, mini-CEX, EPAs, etc.
5. Long-practice observation and evaluation: Observe and evaluate the actual performance of trainees in the workplace during the previous period. This observation can avoid the "Hawthorne effect" of direct observation and evaluation in the workplace for a short period. It is recommended to collect feedback from peers, colleagues, or patients through "multi-source feedback" to confirm student's performance in terms of accountability, communication, teamwork, and resilience.

6. Portfolio: To record the learning progress, including quantitative (for example, number of cases, number of operations) and qualitative (self-evaluation, experience, reflection) content, which can be used as a reference for accumulation of learning experience and self-learning ability. The recommended tools include medical certificate, clinic and inpatient medical records, surgical records, case log, journal reading, case-report, research paper publication.

7. Entrustment / supervision level expected at which stage of training

Schedule Entrustment-Supervision Level	Level 1	Level 2	Level 3	Level 4	Level 5
Before entering R2 training (R1)		✪			
Before entering R3 training (R2)		✪			
Before entering R4 training (R3)			✪		
Before entering R5 training (R4)				✪	
Before completing the training (R5)					✪

Level 1	the learner is allowed to be present and observe, not to enact an EPA
level 2	the learner is allowed to execute the EPA with direct, pro-active supervision, present in the room
level 3	the learner is allowed to carry out the EPA without a supervisor in the room, but quickly available if needed, i.e. with indirect, reactive, supervision
level 4	the learner is allowed to work unsupervised
level 5	the learner is allowed to provide supervision to more junior learners

8. Time period to expiration if not practiced

The entrustment / supervision level should be re-evaluated if relevant professional activities have not been implemented for 1 year.
