# Supplementary material for: Automated Evaluation of Reflection and Feedback Quality in Workplace-Based Assessments by Using Natural Language Processing: Cross-Sectional Competency-Based Medical Education Study
Source: JMIR Med Educ. 2025 Oct 22;11:e81718. doi: 10.2196/81718 (PMC12590046; doi:10.2196/81718)
Supplement: Multimedia Appendix 2 [file mededu_v11i1e81718_app2.pdf]

Multimedia Appendix 2

Table S1. Quantified agreement results (Inter-rater reliability) for expert scoring.

	Resident Reflections			Faculty Feedback		
	IRR	95% CI Lower	95% CI Upper	IRR	95% CI Lower	95% CI Upper
4 Level						
Percent Agreement	0.64	0.59	0.70	0.55	0.50	0.61
Cohen's Kappa	0.49	0.42	0.57	0.38	0.31	0.46
2 Level						
Percent Agreement	0.93	0.90	0.96	0.90	0.87	0.94
Cohen's Kappa	0.82	0.74	0.89	0.78	0.70	0.85

IRR: inter-rater reliability

< 0: Poor agreement.

0.01 - 0.20: Slight agreement.

0.21 - 0.40: Fair agreement.

0.41 - 0.60: Moderate agreement.

0.61 - 0.80: Substantial agreement.

0.81 - 1.00: Almost perfect agreement.
