# Supplementary material for: Automated Evaluation of Reflection and Feedback Quality in Workplace-Based Assessments by Using Natural Language Processing: Cross-Sectional Competency-Based Medical Education Study
Source: JMIR Med Educ. 2025 Oct 22;11:e81718. doi: 10.2196/81718 (PMC12590046; doi:10.2196/81718)
Supplement: Multimedia Appendix 3 [file mededu_v11i1e81718_app3.pdf]

Multimedia Appendix 3

Logistic Regression (LR), Support Vector Machine (SVM), and Bidirectional Encoder Representations from Transformers (BERT) codes in the Google Colaboratory (https://colab.research.google.com/).

Part I Split: python codes used in the study to preprocess data

Part II ML: python codes used in the study to implement Logistic Regression (LR) and Support Vector Machine (SVM) algorithms

Part III Bert: python codes used in the study to implement Bidirectional Encoder Representations from Transformers (BERT) algorithms

Part I

Split

```
from google.colab import drive
drive.mount('/content/drive')
```

```
Mounted at /content/drive
```

```
%cd /content/drive/MyDrive/HE
```

```
/content/drive/MyDrive/HE
```

```
import csv from sklearn.model_selection
import train_test_split
```

```
def ran_split(data_x, data_y, rate):
    return train_test_split(data_x, data_y, test_size=rate, random_state=42
```

```
def w_list_to_csv(x,y4,y2):
    w_data=[]

    for i in range(len(x)):
        w_data.append([x[i],y4[i],y2[i]])

    return w_data
```

```
# Preparing train&eval data for students' records
x_s4 = []
y_s4 = []
x_s2 = []
y_s2 = []

with open('HE_S.csv', newline='', encoding="utf-8") as f:
    rows = csv.reader(f)
    for row in rows:
        x_s4.append(row[0])
        y_s4.append(int(row[1])-1)


        x_s2.append(row[0])
        y_s2.append(int(row[2])-1)

x_train_s2_10, x_test_s2_10, y_train_s2_10, y_test_s2_10=ran_split(x_s2, y_
x_train_s4_10, x_test_s4_10, y_train_s4_10, y_test_s4_10=ran_split(x_s4, y_
x_train_s2_20, x_test_s2_20, y_train_s2_20, y_test_s2_20=ran_split(x_s2, y_
x_train_s4_20, x_test_s4_20, y_train_s4_20, y_test_s4_20=ran_split(x_s4, y_
x_train_s2_25, x_test_s2_25, y_train_s2_25, y_test_s2_25=ran_split(x_s2, y_
x_train_s4_25, x_test_s4_25, y_train_s4_25, y_test_s4_25=ran_split(x_s4, y_
```

```
train_s_10=w_list_to_csv(x_train_s2_10,y_train_s4_10, y_train_s2_10)
with open('train_s_10.csv', 'w') as f:
    writer = csv.writer(f)
    writer.writerows(train_s_10)
```

```
test_s_10=w_list_to_csv(x_test_s4_10,y_test_s4_10,y_test_s2_10)
with open('test_s_10.csv', 'w') as f:
    writer = csv.writer(f)
    writer.writerows(test_s_10)
```

```
train_s_20=w_list_to_csv(x_train_s2_20,y_train_s4_20, y_train_s2_20)
with open('train_s_20.csv', 'w') as f:
    writer = csv.writer(f)
    writer.writerows(train_s_20)
```

```
test_s_20=w_list_to_csv(x_test_s4_20,y_test_s4_20,y_test_s2_20)
with open('test_s_20.csv', 'w') as f:
    writer = csv.writer(f)
    writer.writerows(test_s_20)
```

```
train_s_25=w_list_to_csv(x_train_s4_25,y_train_s4_25,y_train_s2_25)
with open('train_s_25.csv', 'w') as f:
    writer = csv.writer(f)
    writer.writerows(train_s_25)
```

```
test_s_25=w_list_to_csv(x_test_s4_25,y_test_s4_25,y_test_s2_25)
with open('test_s_25.csv', 'w') as f:
    writer = csv.writer(f)
    writer.writerows(test_s_25)
```

```
# Preparing train&eval data for teachers' records
x_t4 = []
y_t4 = []
x_t2 = []
y_t2 = []

with open('HE_T.csv', newline='', encoding="utf-8") as f:
    rows = csv.reader(f)
    for row in rows:
        x_t4.append(row[0])
        y_t4.append(int(row[1])-1)


        x_t2.append(row[0])
        y_t2.append(int(row[2])-1)

x_train_t2_10, x_test_t2_10, y_train_t2_10, y_test_t2_10=ran_split(x_t2, y_t
x_train_t4_10, x_test_t4_10, y_train_t4_10, y_test_t4_10=ran_split(x_t4, y_t
x_train_t2_20, x_test_t2_20, y_train_t2_20, y_test_t2_20=ran_split(x_t2, y_t
x_train_t4_20, x_test_t4_20, y_train_t4_20, y_test_t4_20=ran_split(x_t4, y_t
x_train_t2_25, x_test_t2_25, y_train_t2_25, y_test_t2_25=ran_split(x_t2, y_t
x_train_t4_25, x_test_t4_25, y_train_t4_25, y_test_t4_25=ran_split(x_t4, y_t
```

```
train_t_10=w_list_to_csv(x_train_t2_10,y_train_t4_10,y_train_t2_10)
with open('train_t_10.csv', 'w') as f:
    writer = csv.writer(f)
    writer.writerows(train_t_10)
```

```
test_t_10=w_list_to_csv(x_test_t4_10,y_test_t4_10,y_test_t2_10)
with open('test_t_10.csv', 'w') as f:
    writer = csv.writer(f)
    writer.writerows(test_t_10)
```

```
train_t_20=w_list_to_csv(x_train_t2_20,y_train_t4_20,y_train_t2_20)
with open('train_t_20.csv', 'w') as f:
    writer = csv.writer(f)
    writer.writerows(train_t_20)
```

```
test_t_20=w_list_to_csv(x_test_t4_20,y_test_t4_20,y_test_t2_20)
with open('test_t_20.csv', 'w') as f:
    writer = csv.writer(f)
    writer.writerows(test_t_20)
```

```
train_t_25=w_list_to_csv(x_train_s4_25,y_train_s4_25,y_train_s2_25)
with open('train_t_25.csv', 'w') as f:
    writer = csv.writer(f)
    writer.writerows(train_t_25)
```

```
test_t_25=w_list_to_csv(x_test_t4_25,y_test_t4_25,y_test_t2_25)
with open('test_t_25.csv', 'w') as f:
    writer = csv.writer(f)
    writer.writerows(test_t_25)
```

```
開始使用 AI 編寫或生成程式碼。
```

Part II

ML

```
import pandas as pd
import csv
```

```
from google.colab import drive
drive.mount('/content/drive')
```

```
Mounted at /content/drive
```

```
%cd /content/drive/MyDrive/HE/
```

```
/content/drive/MyDrive/HE
```

```
!pip install ckiptagger
```

顯示隱藏的輸出內容

```
from ckiptagger import WS, POS, NER

ws = WS("../data")
```

顯示隱藏的輸出內容

```
# Preparing train&eval data for 90/10 students' records

x_train_s_10 = []
y_train_s4_10 = []
y_train_s2_10 =[]
x_test_s_10 = []
y_test_s4_10 = []
y_test_s2_10 = []


with open('train_s_10.csv', newline='', encoding="utf-8") as f:
    rows = csv.reader(f)
    for row in rows:
        str_raw.append(str(row[0]))
        str_raw[0]=ws(str_raw)[0]
        row[0]= " ".join(str_raw[0])
        x_train_s_10.append(row[0])
        y_train_s4_10.append(int(row[1]))
        y_train_s2_10.append(int(row[2]))

with open('test_s_10.csv', newline='', encoding="utf-8") as f:
    rows = csv.reader(f)
    for row in rows:
        str_raw = []
        str_raw.append(str(row[0]))
        str_raw[0]=ws(str_raw)[0]
        row[0]= " ".join(str_raw[0])
        x_test_s_10.append(row[0])
        y_test_s4_10.append(int(row[1]))
        y_test_s2_10.append(int(row[2]))

```

```
from sklearn.feature_extraction.text import TfidfVectorizer
```

```
#student 90/10 tfidf

tfidf_vectorizer = TfidfVectorizer()
x_train_s_10_tfidf = tfidf_vectorizer.fit_transform(x_train_s_10)
x_test_s_10_tfidf = tfidf_vectorizer.transform(x_test_s_10)

feature_names_tfidf = tfidf_vectorizer.get_feature_names_out()
```

```
from sklearn.linear_model import LogisticRegression
```

```
classifier_lr_s4_10 = LogisticRegression(class_weight='balanced')
classifier_lr_s4_10.fit(x_train_s_10_tfidf, y_train_s4_10)
```

```
LogisticRegression(class_weight='balanced')
```

```
lr_s4_10_y_pred = classifier_lr_s4_10.predict(x_test_s_10_tfidf)
```

```
from sklearn.metrics import confusion_matrix
import matplotlib.pyplot as plt
```

```
# creates confusion matrix
def create_con_mat(pred, label, label_range, title):

    mat_con = (confusion_matrix(list(pred), label, labels=label_range))

# Setting the attributes
    fig, px = plt.subplots(figsize=(7.5, 7.5))
    px.matshow(mat_con, cmap=plt.cm.YlOrRd, alpha=0.5)
    for m in range(mat_con.shape[0]):
        for n in range(mat_con.shape[1]):
            px.text(x=m,y=n,s=mat_con[m, n], va='center', ha='center', size

# Sets the labels
    plt.xlabel('Predictions', fontsize=16)
    plt.ylabel('Actuals', fontsize=16)
    plt.title(title, fontsize=15)
    plt.show()
```

```
#confusion matrix LR s4 90/10
create_con_mat(lr_s4_10_y_pred, y_test_s4_10, [0,1,2,3], "S4 LR Confusion M
```

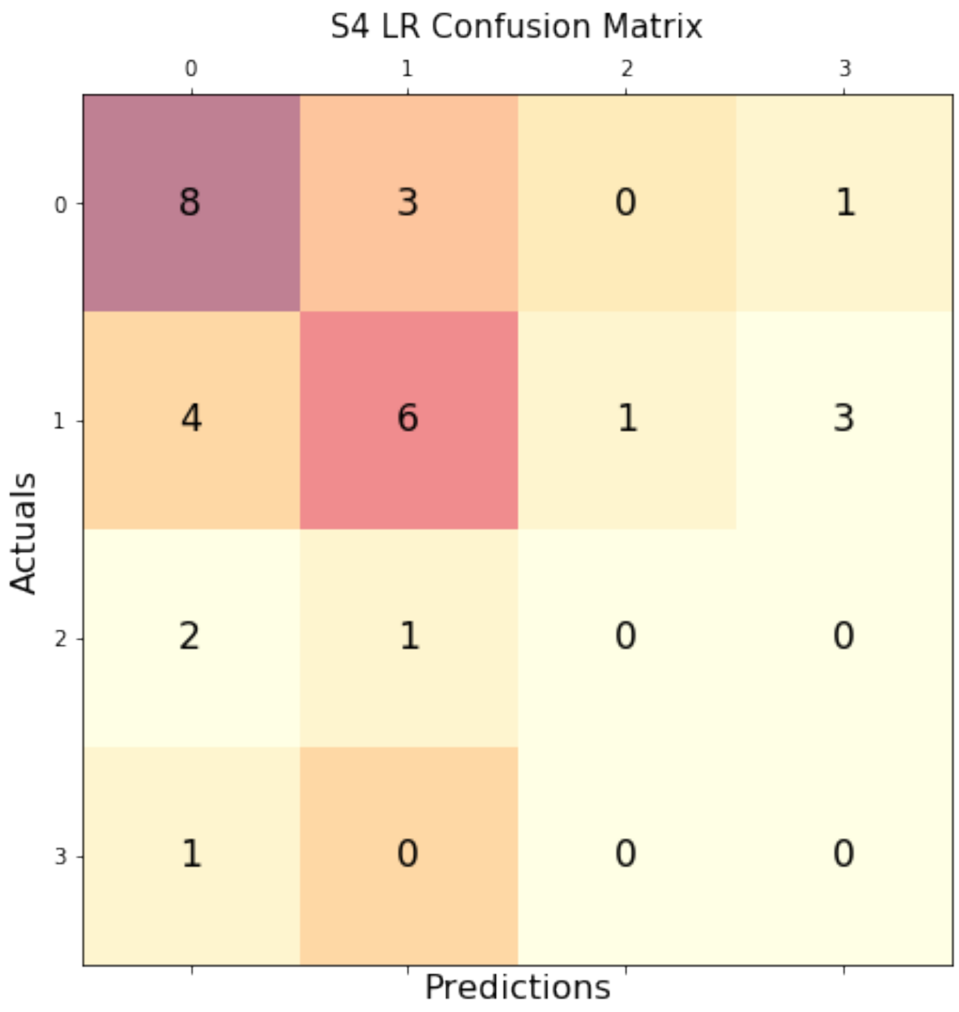


```
from sklearn.metrics import classification_report
print(classification_report(y_test_s4_10, list(lr_s4_10_y_pred), labels=[0,
```

```
              precision    recall  f1-score   support

           0       0.53      0.67      0.59        12
           1       0.60      0.43      0.50        14
           2       0.00      0.00      0.00         3
           3       0.00      0.00      0.00         1

    accuracy                           0.47        30
   macro avg       0.28      0.27      0.27        30
weighted avg       0.49      0.47      0.47        30
```

```
classifier_lr_s2_10 = LogisticRegression(class_weight='balanced')
classifier_lr_s2_10.fit(x_train_s_10_tfidf, y_train_s2_10)
```

```
LogisticRegression(class_weight='balanced')
```

```
lr_s2_10_y_pred = classifier_lr_s2_10.predict(x_test_s_10_tfidf)
```

```
#confusion matrix LR s4 90/10
create_con_mat(lr_s2_10_y_pred, y_test_s2_10, [0,1], "S2 LR Confusion Matri
```

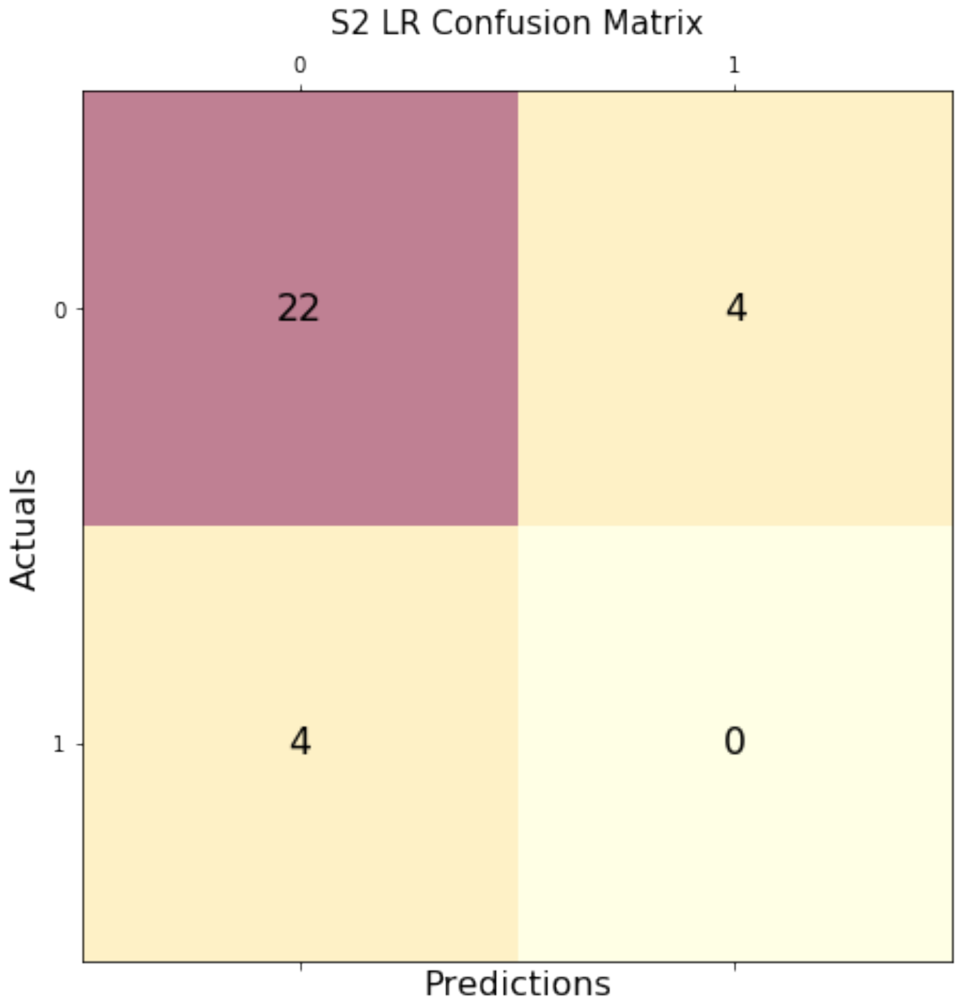


```
print(classification_report(y_test_s2_10, list(lr_s2_10_y_pred), labels=[0,
```

```
              precision    recall  f1-score   support

           0       0.85      0.85      0.85        26
           1       0.00      0.00      0.00         4

    accuracy                           0.73        30
   macro avg       0.42      0.42      0.42        30
weighted avg       0.73      0.73      0.73        30
```

```
from sklearn.svm import SVC
```

```
#SVM s4 90/10
classifier_SVM_s4_10 = SVC(kernel='rbf', class_weight='balanced')  # Use ba
classifier_SVM_s4_10.fit(x_train_s_10_tfidf, y_train_s4_10)

# Prediction and Evaluation
SVM_s4_10_y_pred = classifier_SVM_s4_10.predict(x_test_s_10_tfidf)

#confusion matrix SVM s4 90/10
create_con_mat(SVM_s4_10_y_pred, y_test_s4_10, [0,1,2,3], "S4 SVM Confusior
```

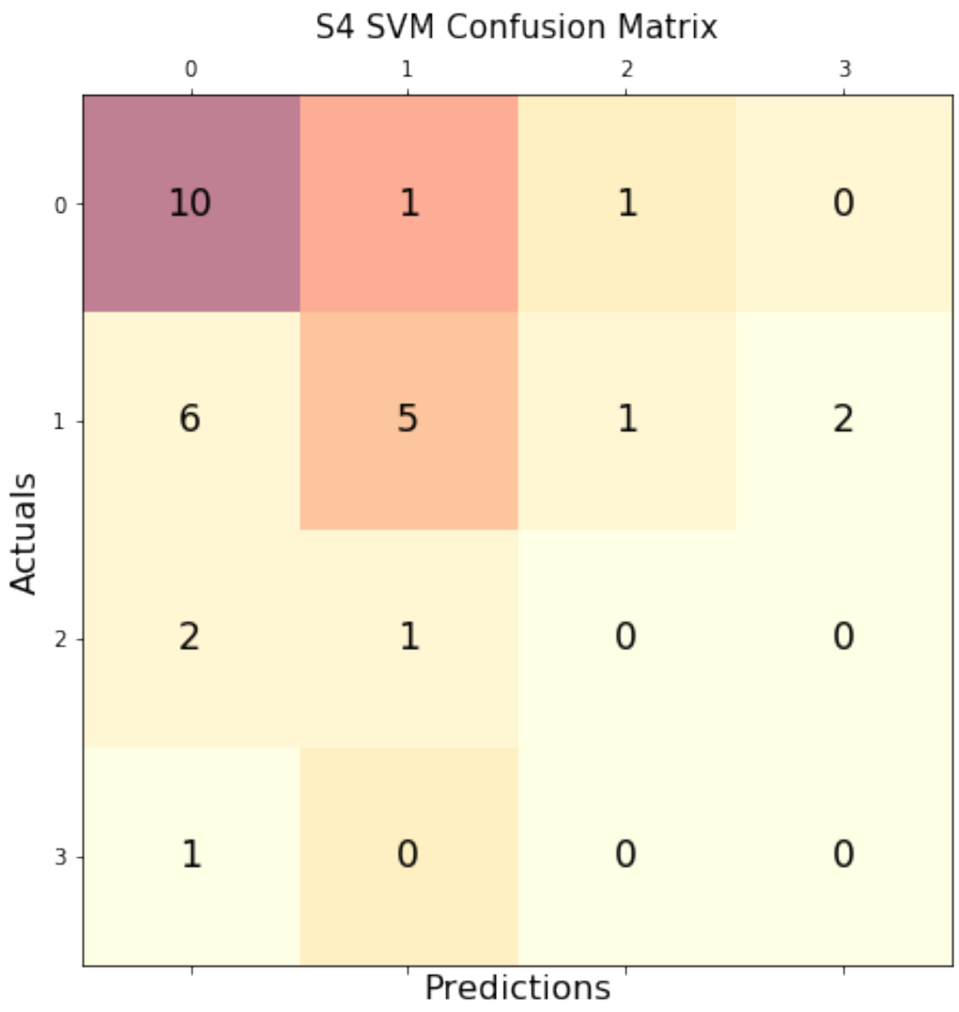


```
print(classification_report(y_test_s4_10, list(SVM_s4_10_y_pred), labels=[0
```

```
              precision    recall  f1-score   support

           0       0.53      0.83      0.65        12
           1       0.71      0.36      0.48        14
           2       0.00      0.00      0.00         3
           3       0.00      0.00      0.00         1

    accuracy                           0.50        30
   macro avg       0.31      0.30      0.28        30
weighted avg       0.54      0.50      0.48        30
```

```
#SVM s2 90/10
classifier_SVM_s2_10 = SVC(kernel='rbf', class_weight='balanced')  # Use ba
classifier_SVM_s2_10.fit(x_train_s_10_tfidf, y_train_s2_10)

# Prediction and Evaluation
SVM_s2_10_y_pred = classifier_SVM_s2_10.predict(x_test_s_10_tfidf)

#confusion matrix SVM s2 90/10
create_con_mat(SVM_s2_10_y_pred, y_test_s2_10, [0,1], "S2 SVM Confusion Mat
```

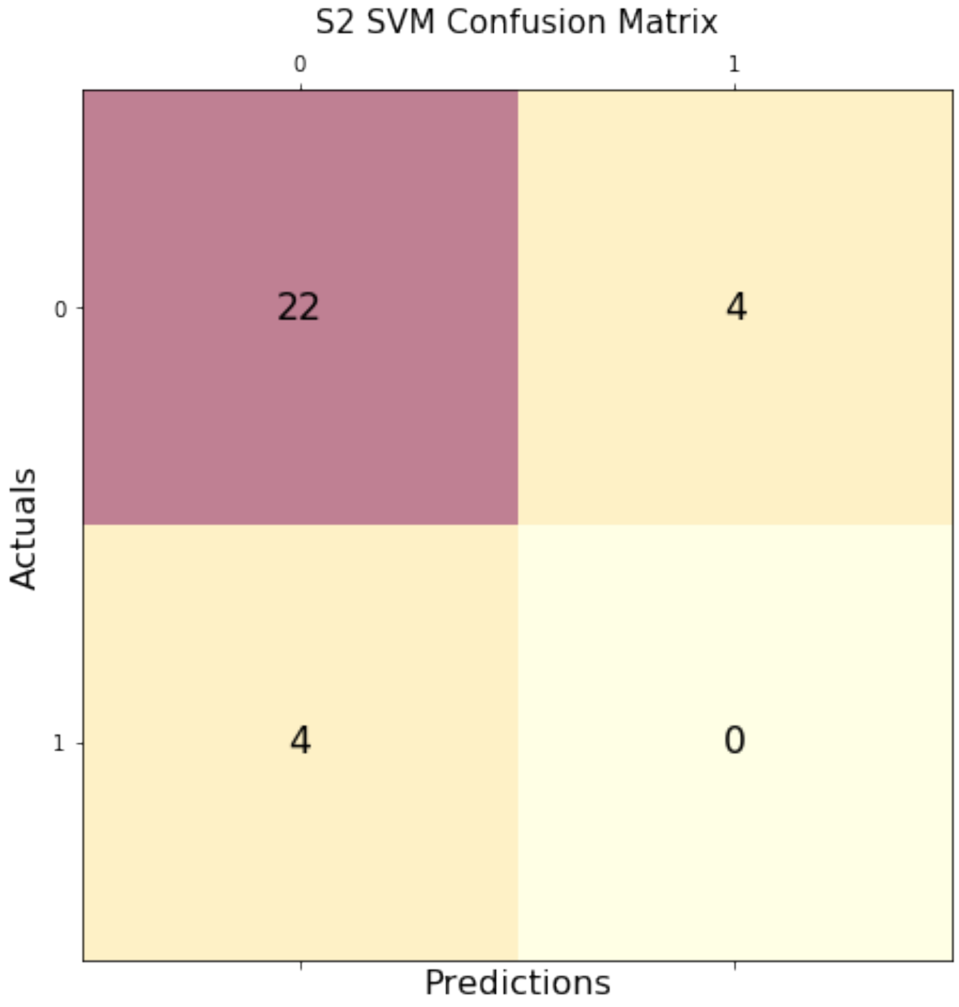


```
print(classification_report(y_test_s2_10, list(SVM_s2_10_y_pred), labels=[0
```

```
              precision    recall  f1-score   support

           0       0.85      0.85      0.85        26
           1       0.00      0.00      0.00         4

    accuracy                           0.73        30
   macro avg       0.42      0.42      0.42        30
weighted avg       0.73      0.73      0.73        30
```

```python
# Preparing train&eval data for 90/10 Teachers' records

x_train_t_10 = []
y_train_t4_10 = []
y_train_t2_10 =[]
x_test_t_10 = []
y_test_t4_10 = []
y_test_t2_10 = []

with open('train_t_10.csv', newline='', encoding="utf-8") as f:
    rows = csv.reader(f)
    for row in rows:
        str_raw = []
        str_raw.append(str(row[0]))
        str_raw[0]=ws(str_raw)[0]
        row[0]= " ".join(str_raw[0])
        x_train_t_10.append(row[0])
        y_train_t4_10.append(int(row[1]))
        y_train_t2_10.append(int(row[2]))

with open('test_t_10.csv', newline='', encoding="utf-8") as f:
    rows = csv.reader(f)
    for row in rows:
        str_raw = []
        str_raw.append(str(row[0]))
        str_raw[0]=ws(str_raw)[0]
        row[0]= " ".join(str_raw[0])
        x_test_t_10.append(row[0])
        y_test_t4_10.append(int(row[1]))
        y_test_t2_10.append(int(row[2]))

```

```python
#teacher 90/10 tfidf

tfidf_vectorizer = TfidfVectorizer()
x_train_t_10_tfidf = tfidf_vectorizer.fit_transform(x_train_t_10)
x_test_t_10_tfidf = tfidf_vectorizer.transform(x_test_t_10)

feature_names_tfidf = tfidf_vectorizer.get_feature_names_out()

```

```
#T4 LR 90/10

classifier_lr_t4_10 = LogisticRegression(class_weight='balanced')
classifier_lr_t4_10.fit(x_train_t_10_tfidf, y_train_t4_10)

lr_t4_10_y_pred = classifier_lr_t4_10.predict(x_test_t_10_tfidf)

create_con_mat(lr_t4_10_y_pred, y_test_t4_10, [0,1,2,3], "T4 LR Confusion M

```

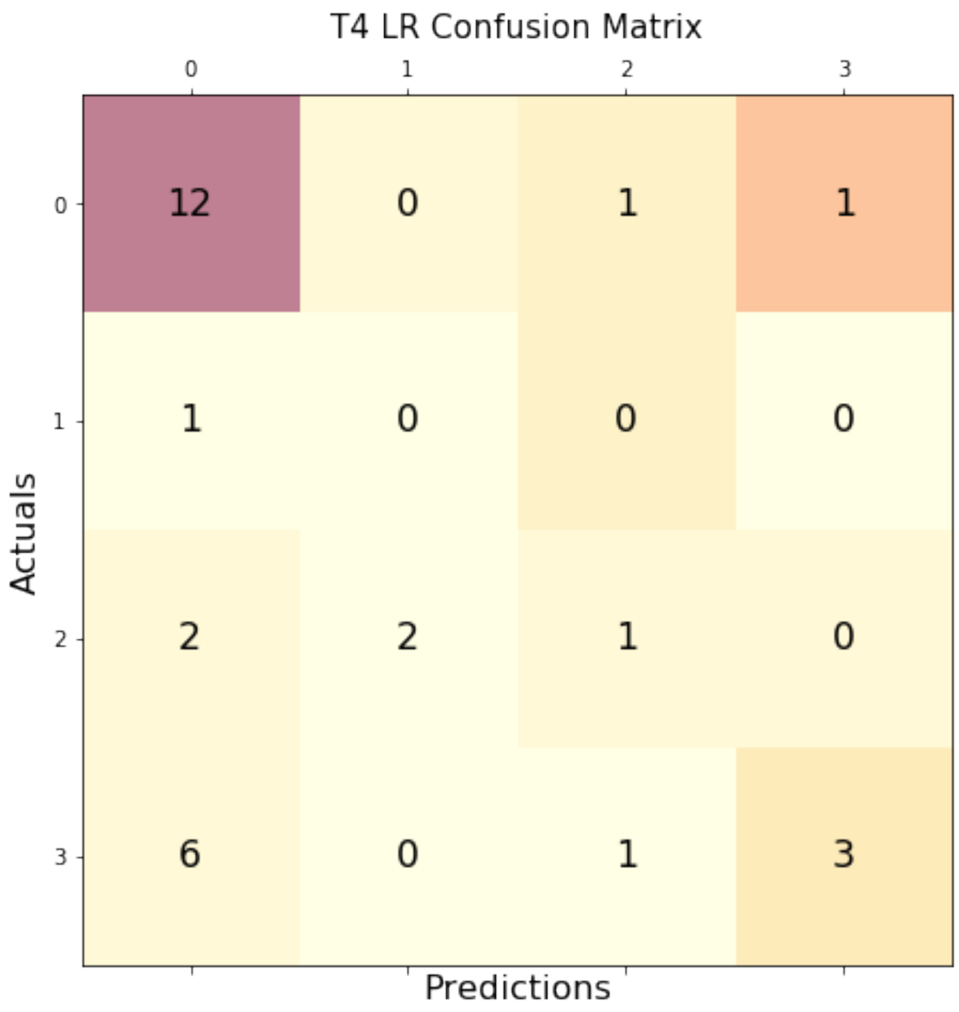


```
print(classification_report(y_test_t4_10, list(lr_t4_10_y_pred), labels=[0,
```

```
              precision    recall  f1-score   support

           0       0.57      0.86      0.69        14
           1       0.00      0.00      0.00         1
           2       0.33      0.20      0.25         5
           3       0.75      0.30      0.43        10

    accuracy                           0.53        30
   macro avg       0.41      0.34      0.34        30
weighted avg       0.57      0.53      0.50        30
```

```
#T2 LR 90/10

classifier_lr_t2_10 = LogisticRegression(class_weight='balanced')
classifier_lr_t2_10.fit(x_train_t_10_tfidf, y_train_t2_10)

lr_t2_10_y_pred = classifier_lr_t2_10.predict(x_test_t_10_tfidf)

create_con_mat(lr_t2_10_y_pred, y_test_t2_10, [0,1], "T2 LR Confusion Matri

```

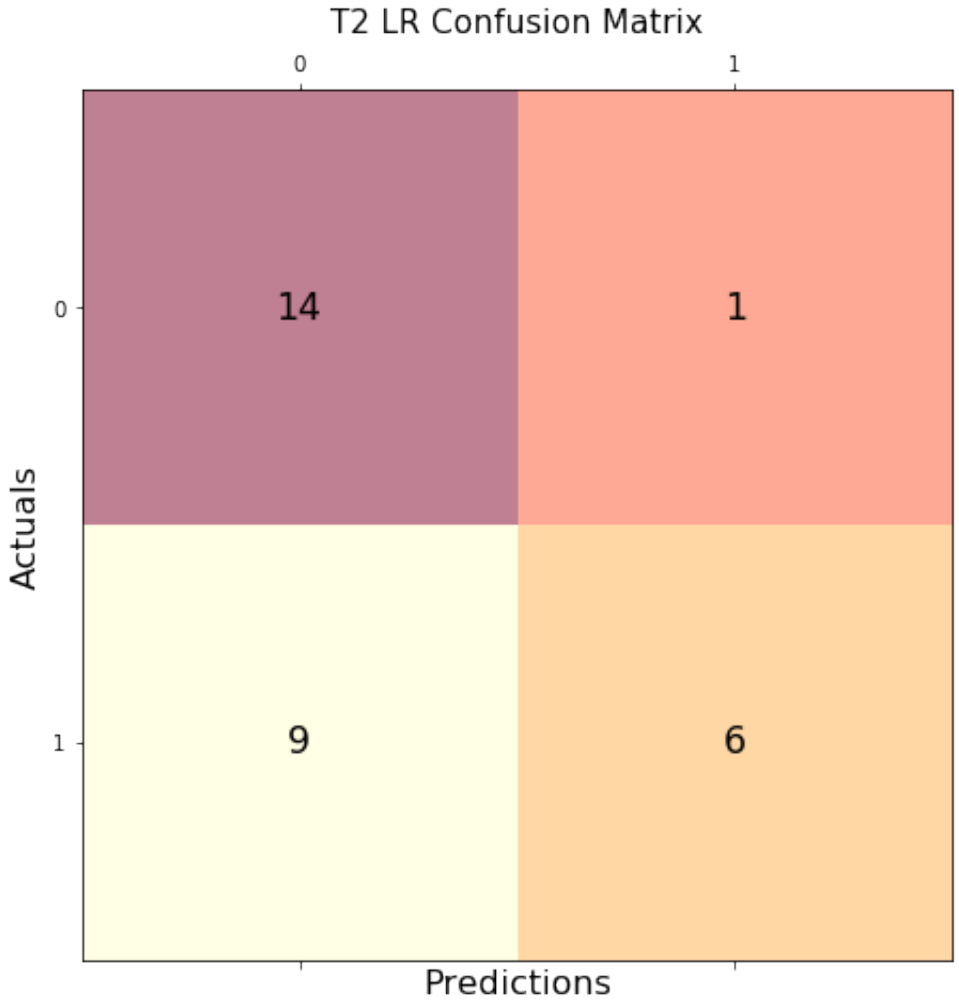


```
print(classification_report(y_test_t2_10, list(lr_t2_10_y_pred), labels=[0,
```

```
              precision    recall  f1-score   support

           0       0.61      0.93      0.74        15
           1       0.86      0.40      0.55        15

    accuracy                           0.67        30
   macro avg       0.73      0.67      0.64        30
weighted avg       0.73      0.67      0.64        30
```

```
#SVM t4 90/10
classifier_SVM_t4_10 = SVC(kernel='rbf', class_weight='balanced')  # Use ba
classifier_SVM_t4_10.fit(x_train_t_10_tfidf, y_train_t4_10)

# Prediction and Evaluation
SVM_t4_10_y_pred = classifier_SVM_t4_10.predict(x_test_t_10_tfidf)

#confusion matrix SVM t4 90/10
create_con_mat(SVM_t4_10_y_pred, y_test_t4_10, [0,1,2,3], "T4 SVM Confusior

```

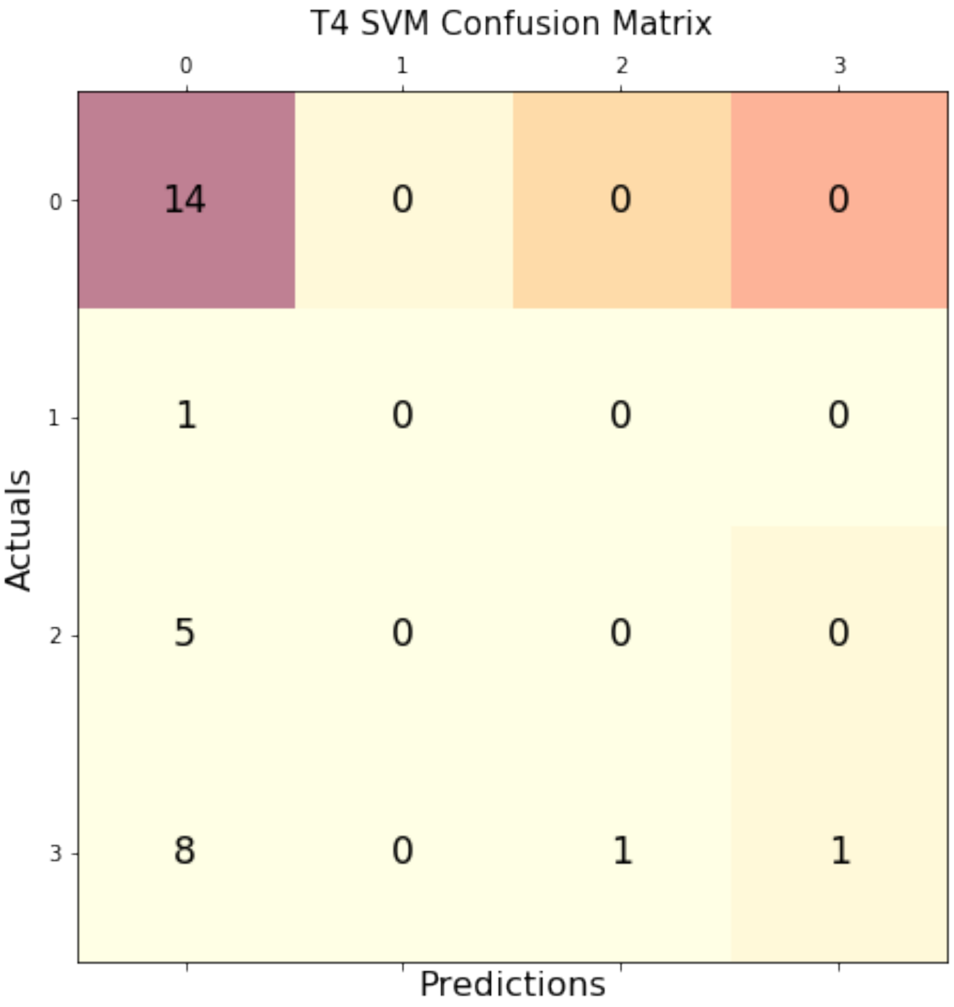

```
1 print(classification_report(y_test_t4_10, list(SVM_t4_10_y_pred), labels=[0
```

```
              precision    recall  f1-score   support

           0       0.50      1.00      0.67        14
           1       0.00      0.00      0.00         1
           2       0.00      0.00      0.00         5
           3       1.00      0.10      0.18        10

    accuracy                           0.50        30
   macro avg       0.38      0.28      0.21        30
weighted avg       0.57      0.50      0.37        30

/usr/local/lib/python3.7/site-packages/sklearn/metrics/_classification.py:1
  _warn_prf(average, modifier, msg_start, len(result))
/usr/local/lib/python3.7/site-packages/sklearn/metrics/_classification.py:1
  _warn_prf(average, modifier, msg_start, len(result))
/usr/local/lib/python3.7/site-packages/sklearn/metrics/_classification.py:1
  _warn_prf(average, modifier, msg_start, len(result))
```

```
#SVM t2 90/10
classifier_SVM_t2_10 = SVC(kernel='rbf', class_weight='balanced')  # Use ba
classifier_SVM_t2_10.fit(x_train_t_10_tfidf, y_train_t2_10)

# Prediction and Evaluation
SVM_t2_10_y_pred = classifier_SVM_t2_10.predict(x_test_t_10_tfidf)

#confusion matrix SVM t2 90/10
create_con_mat(SVM_t2_10_y_pred, y_test_t2_10, [0,1], "T2 SVM Confusion Mat


```

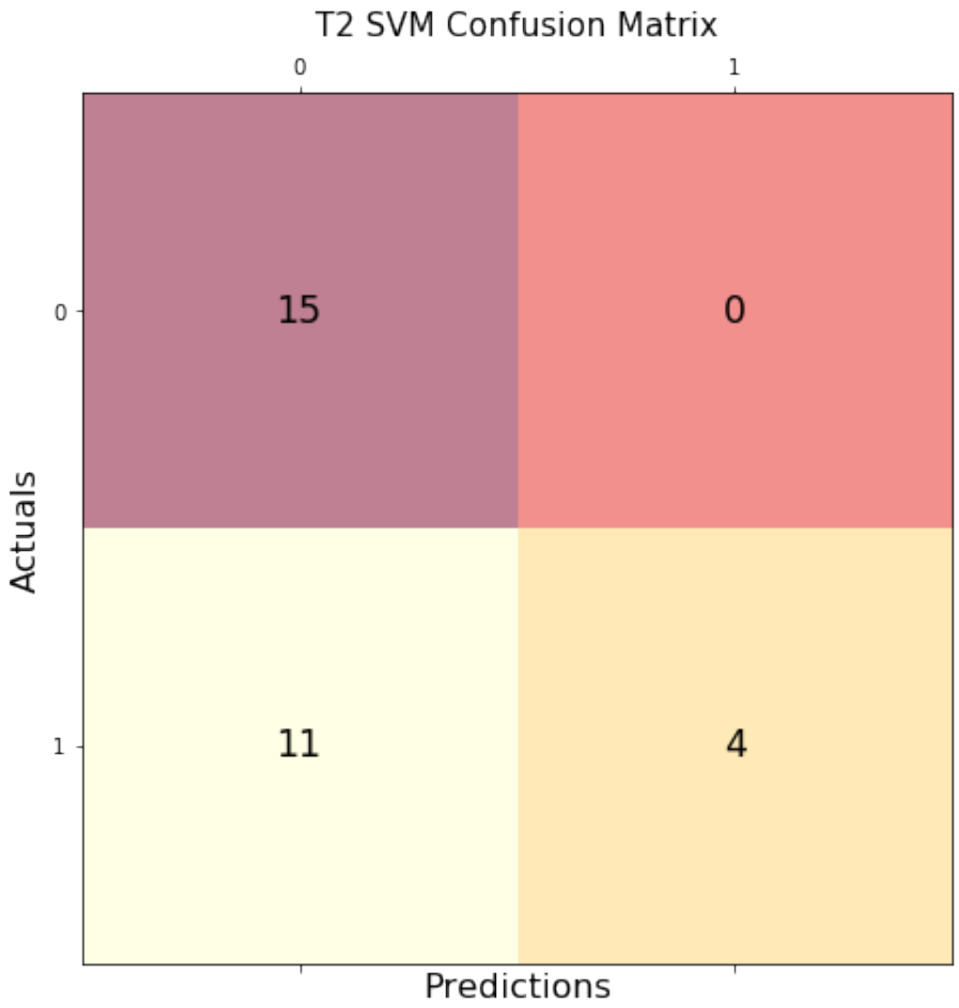

```
print(classification_report(y_test_t2_10, list(SVM_t2_10_y_pred), labels=[0
```

```
              precision    recall  f1-score   support

           0       0.58      1.00      0.73        15
           1       1.00      0.27      0.42        15

    accuracy                           0.63        30
   macro avg       0.79      0.63      0.58        30
weighted avg       0.79      0.63      0.58        30
```

```
# Preparing train&eval data for 80/20 students' records
x_train_s_20 = []
y_train_s4_20 = []
y_train_s2_20 =[]
x_test_s_20 = []
y_test_s4_20 = []
y_test_s2_20 = []

with open('train_s_20.csv', newline='', encoding="utf-8") as f:
    rows = csv.reader(f)
    for row in rows:
        str_raw.append(str(row[0]))
        str_raw[0]=ws(str_raw)[0]
        row[0]= " ".join(str_raw[0])
        x_train_s_20.append(row[0])
        y_train_s4_20.append(int(row[1]))
        y_train_s2_20.append(int(row[2]))

with open('test_s_20.csv', newline='', encoding="utf-8") as f:
    rows = csv.reader(f)
    for row in rows:
        str_raw = []
        str_raw.append(str(row[0]))
        str_raw[0]=ws(str_raw)[0]
        row[0]= " ".join(str_raw[0])
        x_test_s_20.append(row[0])
        y_test_s4_20.append(int(row[1]))
        y_test_s2_20.append(int(row[2]))
```

```
#student 80/20 tfidf

tfidf_vectorizer = TfidfVectorizer()
x_train_s_20_tfidf = tfidf_vectorizer.fit_transform(x_train_s_20)
x_test_s_20_tfidf = tfidf_vectorizer.transform(x_test_s_20)

feature_names_tfidf = tfidf_vectorizer.get_feature_names_out()
```

```
classifier_lr_s4_20 = LogisticRegression(class_weight='balanced')
classifier_lr_s4_20.fit(x_train_s_20_tfidf, y_train_s4_20)

lr_s4_20_y_pred = classifier_lr_s4_20.predict(x_test_s_20_tfidf)
```

```
#confusion matrix LR s4 80/20
create_con_mat(lr_s4_20_y_pred, y_test_s4_20, [0,1,2,3], "Confusion Matrix L
```

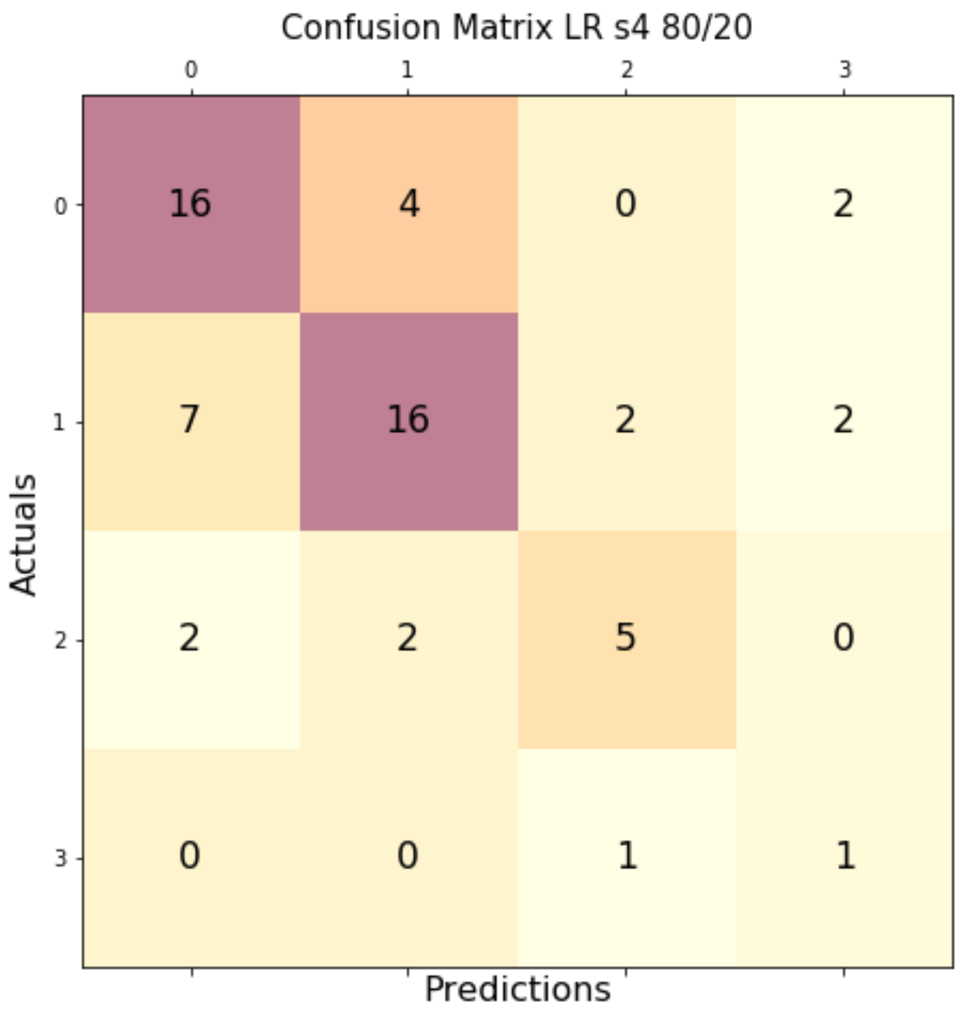


```
print(classification_report(y_test_s4_20, list(lr_s4_20_y_pred), labels=[0,
```

```
              precision    recall  f1-score   support

           0       0.64      0.73      0.68        22
           1       0.73      0.59      0.65        27
           2       0.62      0.56      0.59         9
           3       0.20      0.50      0.29         2

    accuracy                           0.63        60
   macro avg       0.55      0.59      0.55        60
weighted avg       0.66      0.63      0.64        60
```

```python
classifier_lr_s2_20 = LogisticRegression(class_weight='balanced')
classifier_lr_s2_20.fit(x_train_s_20_tfidf, y_train_s2_20)

lr_s2_20_y_pred = classifier_lr_s2_20.predict(x_test_s_20_tfidf)

#confusion matrix LR s2 80/20
create_con_mat(lr_s2_20_y_pred, y_test_s2_20, [0,1], "Confusion Matrix LR s
```

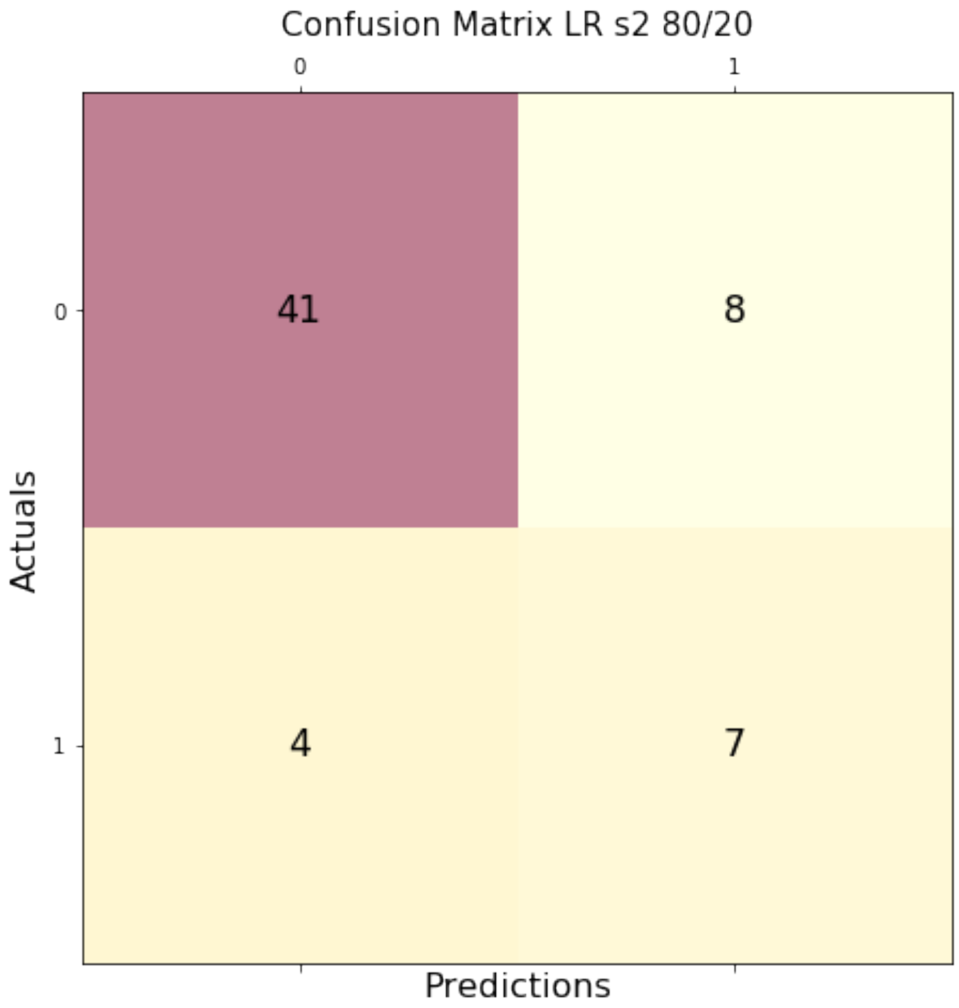


```python
print(classification_report(y_test_s2_20, list(lr_s2_20_y_pred), labels=[0,
```

```
              precision    recall  f1-score   support

           0       0.91      0.84      0.87        49
           1       0.47      0.64      0.54        11

    accuracy                           0.80        60
   macro avg       0.69      0.74      0.71        60
weighted avg       0.83      0.80      0.81        60
```

```
#SVM s4 80/20
classifier_SVM_s4_20 = SVC(kernel='rbf', class_weight='balanced')  # Use ba
classifier_SVM_s4_20.fit(x_train_s_20_tfidf, y_train_s4_20)

# Prediction and Evaluation
SVM_s4_20_y_pred = classifier_SVM_s4_20.predict(x_test_s_20_tfidf)

#confusion matrix SVM s4 80/20
create_con_mat(SVM_s4_20_y_pred, y_test_s4_20, [0,1,2,3], "Confusion Matrix
```

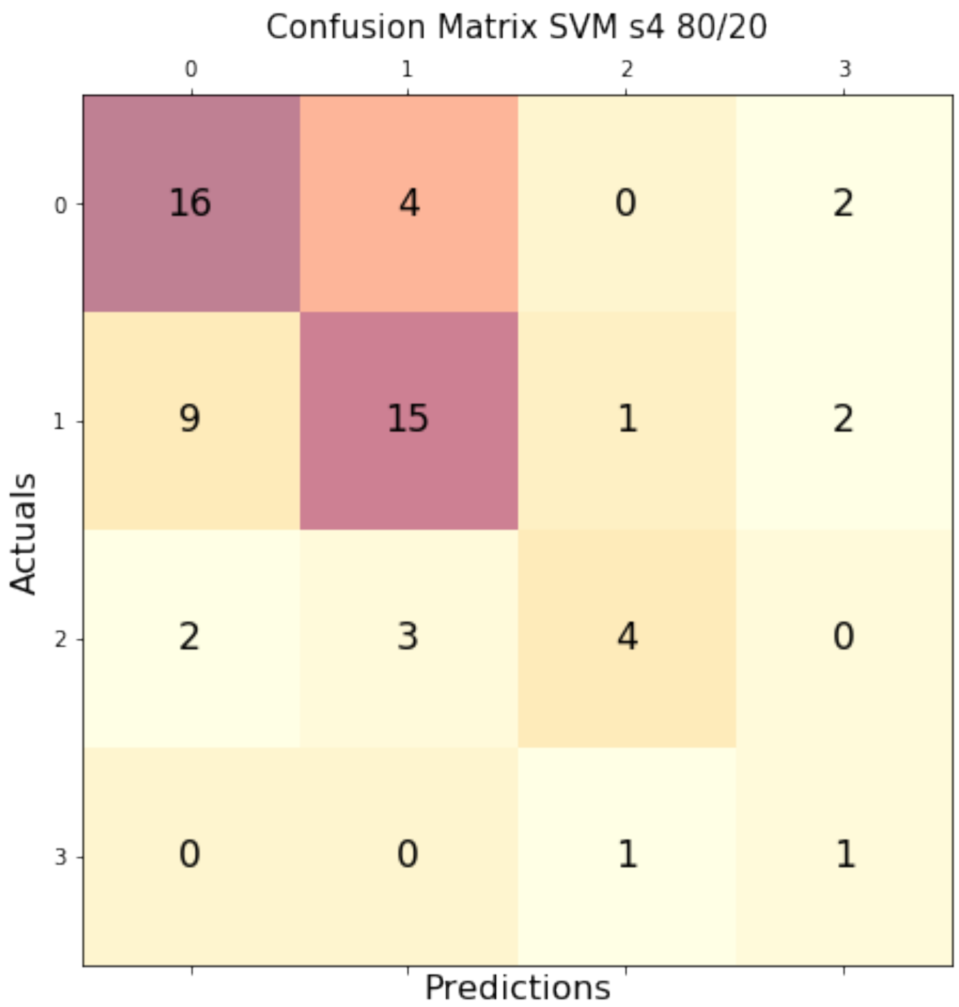


```
print(classification_report(y_test_s4_20, list(SVM_s4_20_y_pred), labels=[0
```

```
              precision    recall  f1-score   support

           0       0.59      0.73      0.65        22
           1       0.68      0.56      0.61        27
           2       0.67      0.44      0.53         9
           3       0.20      0.50      0.29         2

    accuracy                           0.60        60
   macro avg       0.54      0.56      0.52        60
weighted avg       0.63      0.60      0.60        60
```

```
#SVM s2 80/20
classifier_SVM_s2_20 = SVC(kernel='rbf', class_weight='balanced')  # Use ba
classifier_SVM_s2_20.fit(x_train_s_20_tfidf, y_train_s2_20)

# Prediction and Evaluation
SVM_s2_20_y_pred = classifier_SVM_s2_20.predict(x_test_s_20_tfidf)

#confusion matrix SVM s4 80/20
create_con_mat(SVM_s2_20_y_pred, y_test_s2_20, [0,1], "Confusion Matrix SVM
```

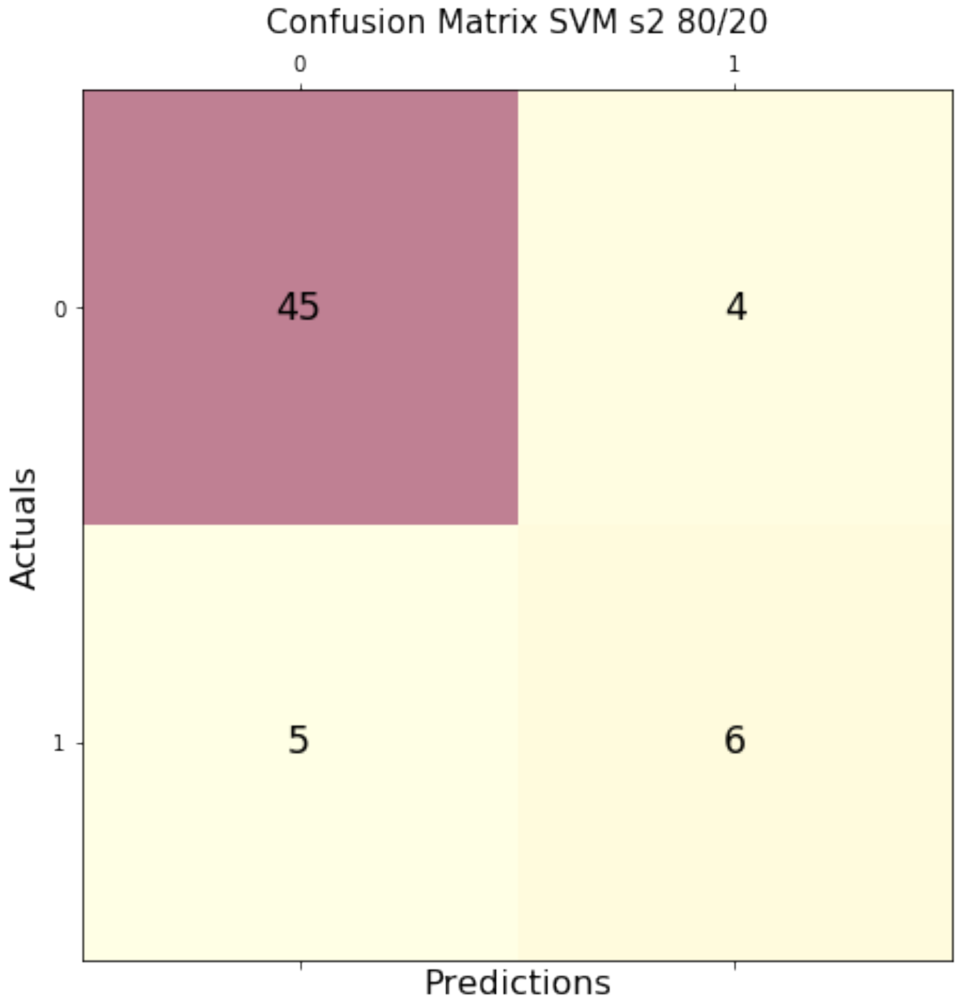


```
print(classification_report(y_test_s2_20, list(SVM_s2_20_y_pred), labels=[0
```

```
              precision    recall  f1-score   support

           0       0.90      0.92      0.91        49
           1       0.60      0.55      0.57        11

    accuracy                           0.85        60
   macro avg       0.75      0.73      0.74        60
weighted avg       0.85      0.85      0.85        60
```

```
# Preparing train&eval data for 75/25 students' records
x_train_s_25 = []
y_train_s4_25 = []
y_train_s2_25 =[]
x_test_s_25 = []
y_test_s4_25 = []
y_test_s2_25 = []

with open('train_s_25.csv', newline='', encoding="utf-8") as f:
    rows = csv.reader(f)
    for row in rows:
        str_raw = []
        str_raw.append(str(row[0]))
        str_raw[0]=ws(str_raw)[0]
        row[0]= " ".join(str_raw[0])
        x_train_s_25.append(row[0])
        y_train_s4_25.append(int(row[1]))
        y_train_s2_25.append(int(row[2]))

with open('test_s_25.csv', newline='', encoding="utf-8") as f:
    rows = csv.reader(f)
    for row in rows:
        count+=1
        print(count)
        str_raw = []
        str_raw.append(str(row[0]))
        str_raw[0]=ws(str_raw)[0]
        row[0]= " ".join(str_raw[0])
        x_test_s_25.append(row[0])
        y_test_s4_25.append(int(row[1]))
        y_test_s2_25.append(int(row[2]))
```

```
#student 75/25 tfidf

tfidf_vectorizer = TfidfVectorizer()
x_train_s_25_tfidf = tfidf_vectorizer.fit_transform(x_train_s_25)
x_test_s_25_tfidf = tfidf_vectorizer.transform(x_test_s_25)

feature_names_tfidf = tfidf_vectorizer.get_feature_names_out()
```

```
classifier_lr_s4_25 = LogisticRegression(class_weight='balanced')
classifier_lr_s4_25.fit(x_train_s_25_tfidf, y_train_s4_25)

lr_s4_25_y_pred = classifier_lr_s4_25.predict(x_test_s_25_tfidf)

#confusion matrix LR s4 75/25
create_con_mat(lr_s4_25_y_pred, y_test_s4_25, [0,1,2,3], "Confusion Matrix
```

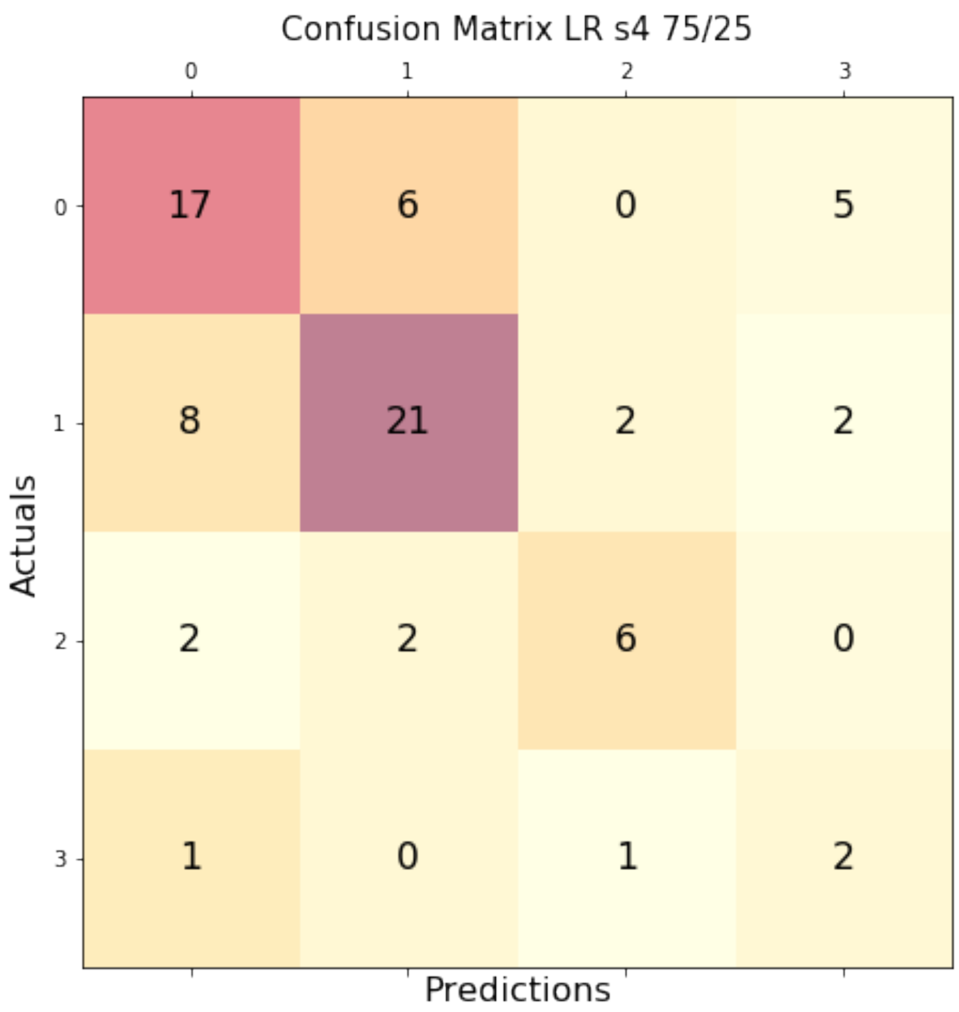


```
print(classification_report(y_test_s4_25, list(lr_s4_25_y_pred), labels=[0,
```

```
              precision    recall  f1-score   support

           0       0.61      0.61      0.61        28
           1       0.72      0.64      0.68        33
           2       0.67      0.60      0.63        10
           3       0.22      0.50      0.31         4

    accuracy                           0.61        75
   macro avg       0.56      0.59      0.56        75
weighted avg       0.65      0.61      0.63        75
```

```python
classifier_lr_s2_25 = LogisticRegression(class_weight='balanced')
classifier_lr_s2_25.fit(x_train_s_25_tfidf, y_train_s2_25)

lr_s2_25_y_pred = classifier_lr_s2_25.predict(x_test_s_25_tfidf)

#confusion matrix LR s4 75/25
create_con_mat(lr_s2_25_y_pred, y_test_s2_25, [0,1], "Confusion Matrix LR s
```

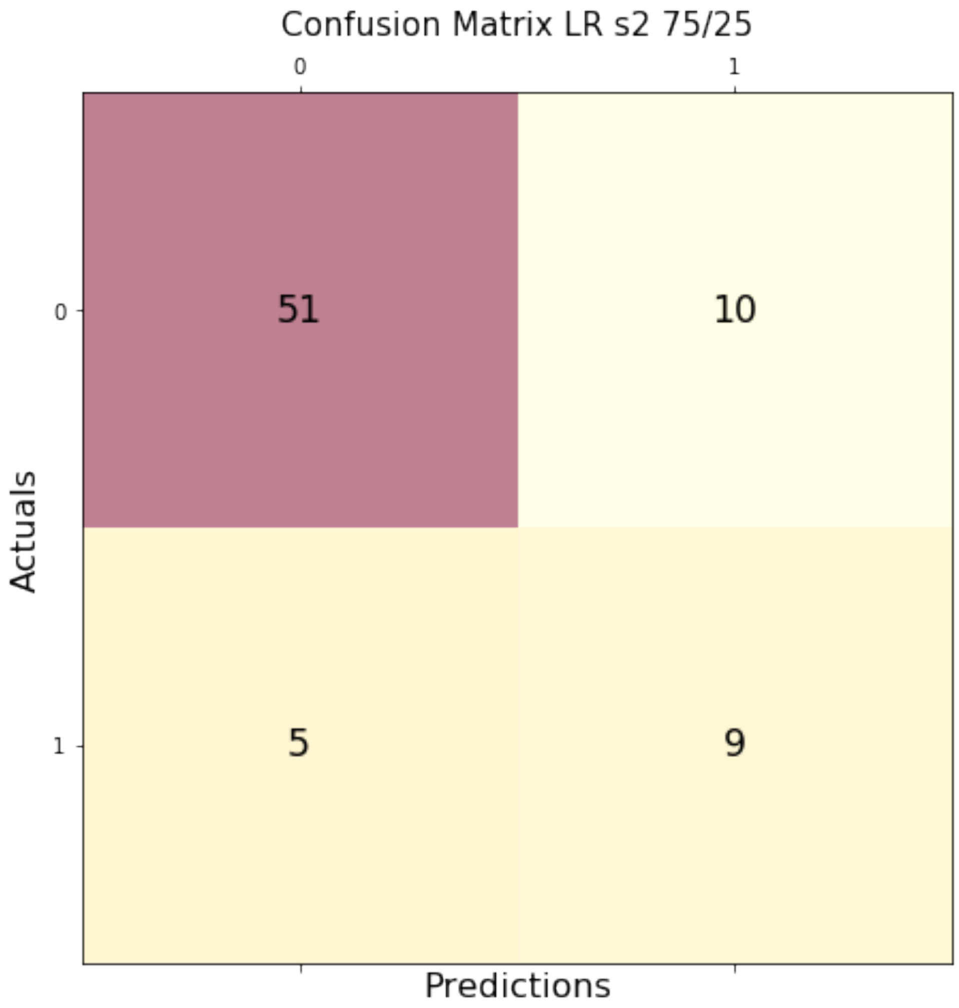


```python
print(classification_report(y_test_s2_25, list(lr_s2_25_y_pred), labels=[0,
```

```
              precision    recall  f1-score   support

           0       0.91      0.84      0.87        61
           1       0.47      0.64      0.55        14

    accuracy                           0.80        75
   macro avg       0.69      0.74      0.71        75
weighted avg       0.83      0.80      0.81        75
```

```
#SVM s4 75/25
classifier_SVM_s4_25 = SVC(kernel='rbf', class_weight='balanced')  # Use ba
classifier_SVM_s4_25.fit(x_train_s_25_tfidf, y_train_s4_25)

# Prediction and Evaluation
SVM_s4_25_y_pred = classifier_SVM_s4_25.predict(x_test_s_25_tfidf)

#confusion matrix SVM s4 80/20
create_con_mat(SVM_s4_25_y_pred, y_test_s4_25, [0,1,2,3], "Confusion Matrix
```

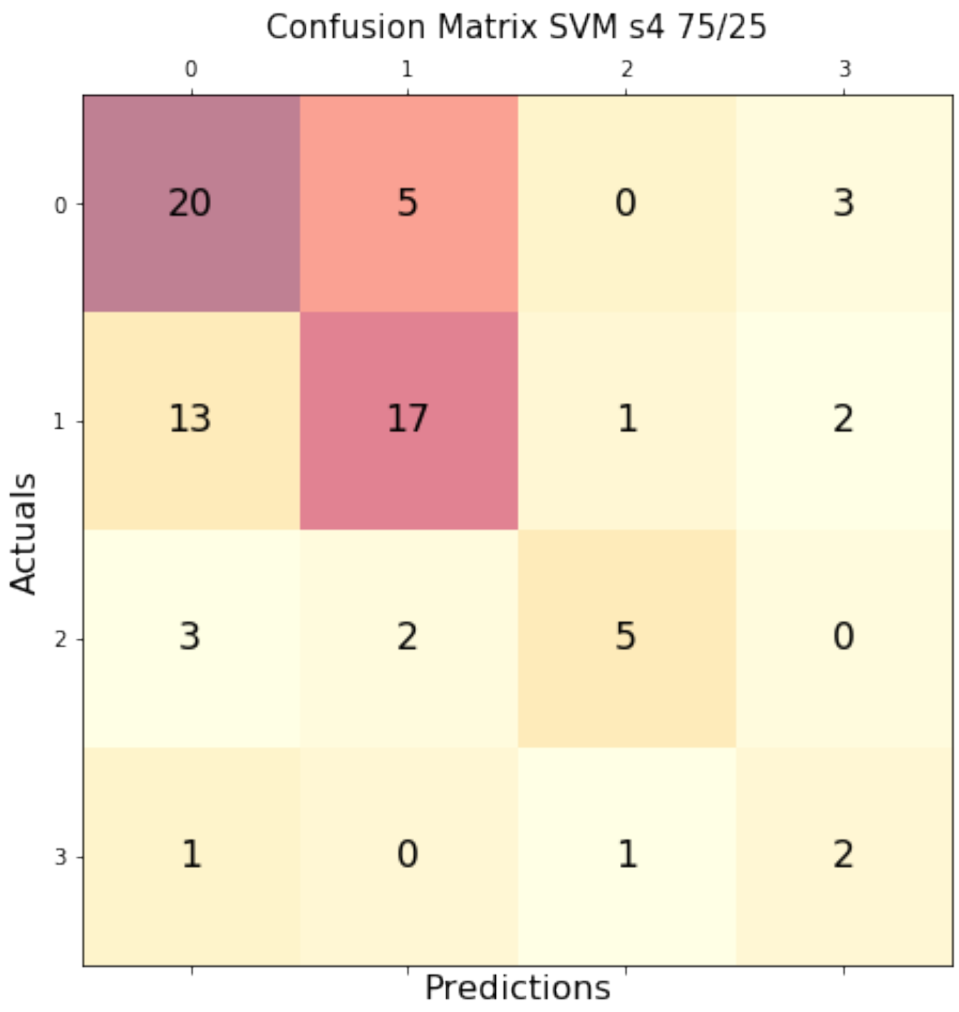


```
print(classification_report(y_test_s4_25, list(SVM_s4_25_y_pred), labels=[0
```

```
              precision    recall  f1-score   support

           0       0.54      0.71      0.62        28
           1       0.71      0.52      0.60        33
           2       0.71      0.50      0.59        10
           3       0.29      0.50      0.36         4

    accuracy                           0.59        75
   macro avg       0.56      0.56      0.54        75
weighted avg       0.62      0.59      0.59        75
```

```
#SVM s2 75/25
classifier_SVM_s2_25 = SVC(kernel='rbf', class_weight='balanced')  # Use ba
classifier_SVM_s2_25.fit(x_train_s_25_tfidf, y_train_s2_25)

# Prediction and Evaluation
SVM_s2_25_y_pred = classifier_SVM_s2_25.predict(x_test_s_25_tfidf)

#confusion matrix SVM s2 75/25
create_con_mat(SVM_s2_25_y_pred, y_test_s2_25, [0,1], "Confusion Matrix SVM
```

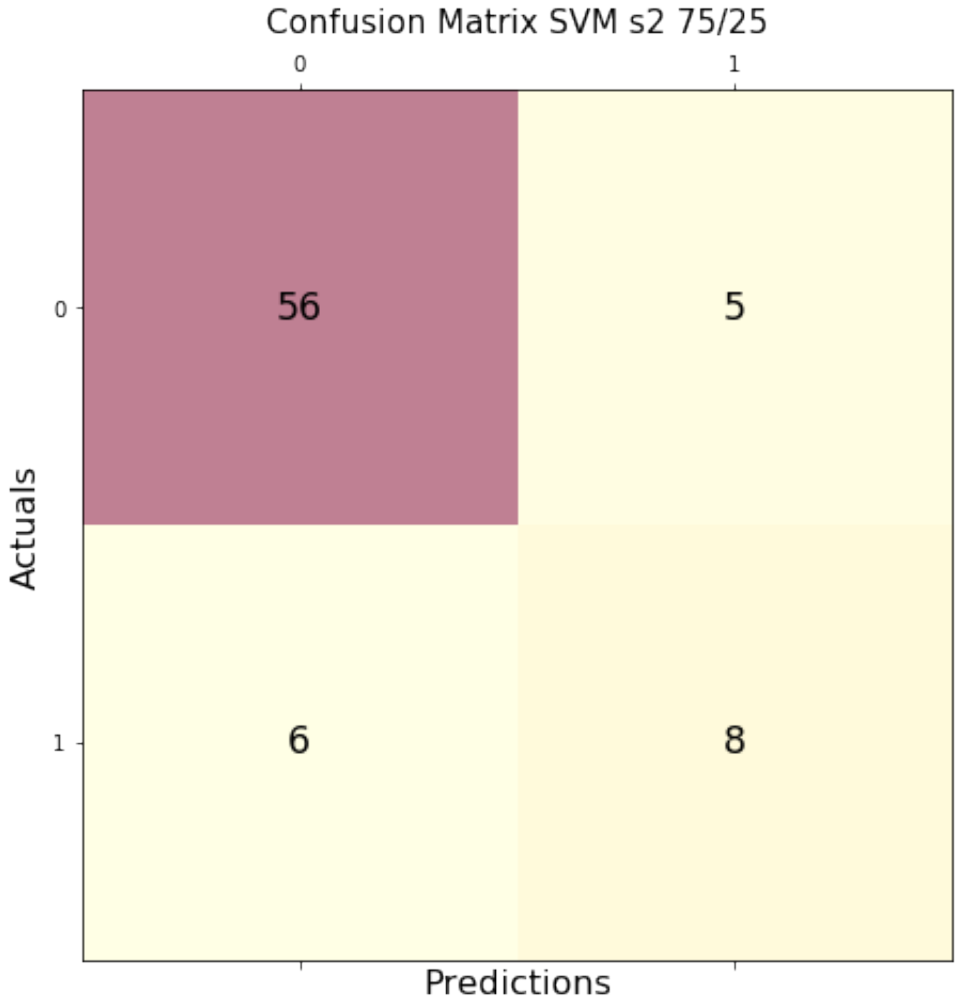


```
print(classification_report(y_test_s2_25, list(SVM_s2_25_y_pred), labels=[0
```

```
              precision    recall  f1-score   support

           0       0.90      0.92      0.91        61
           1       0.62      0.57      0.59        14

    accuracy                           0.85        75
   macro avg       0.76      0.74      0.75        75
weighted avg       0.85      0.85      0.85        75
```

```
# Preparing train&eval data for 80/20 teachers' records
x_train_t_20 = []
y_train_t4_20 = []
y_train_t2_20 =[]
x_test_t_20 = []
y_test_t4_20 = []
y_test_t2_20 = []

with open('train_t_20.csv', newline='', encoding="utf-8") as f:
    rows = csv.reader(f)
    for row in rows:
        str_raw = []
        str_raw.append(str(row[0]))
        str_raw[0]=ws(str_raw)[0]
        row[0]= " ".join(str_raw[0])
        x_train_t_20.append(row[0])
        y_train_t4_20.append(int(row[1]))
        y_train_t2_20.append(int(row[2]))

with open('test_t_20.csv', newline='', encoding="utf-8") as f:
    rows = csv.reader(f)
    for row in rows:
        str_raw = []
        str_raw.append(str(row[0]))
        str_raw[0]=ws(str_raw)[0]
        row[0]= " ".join(str_raw[0])
        x_test_t_20.append(row[0])
        y_test_t4_20.append(int(row[1]))
        y_test_t2_20.append(int(row[2]))
```

```
# Preparing train&eval data for 75/25 teachers' records
x_train_t_25 = []
y_train_t4_25 = []
y_train_t2_25 =[]
x_test_t_25 = []
y_test_t4_25 = []
y_test_t2_25 = []

with open('train_t_25.csv', newline='', encoding="utf-8") as f:
    rows = csv.reader(f)
    for row in rows:
        str_raw = []
        str_raw.append(str(row[0]))
        str_raw[0]=ws(str_raw)[0]
        row[0]= " ".join(str_raw[0])
        x_train_t_25.append(row[0])
        y_train_t4_25.append(int(row[1]))
        y_train_t2_25.append(int(row[2]))

with open('test_t_25.csv', newline='', encoding="utf-8") as f:
    rows = csv.reader(f)
    for row in rows:
        str_raw = []
        str_raw.append(str(row[0]))
        str_raw[0]=ws(str_raw)[0]
        row[0]= " ".join(str_raw[0])
        x_test_t_25.append(row[0])
        y_test_t4_25.append(int(row[1]))
        y_test_t2_25.append(int(row[2]))
```

```
#teacher 80/20 tfidf

tfidf_vectorizer = TfidfVectorizer()
x_train_t_20_tfidf = tfidf_vectorizer.fit_transform(x_train_t_20)
x_test_t_20_tfidf = tfidf_vectorizer.transform(x_test_t_20)


feature_names_tfidf = tfidf_vectorizer.get_feature_names_out()
```

```python
classifier_lr_t4_20 = LogisticRegression(class_weight='balanced')
classifier_lr_t4_20.fit(x_train_t_20_tfidf, y_train_t4_20)

lr_t4_20_y_pred = classifier_lr_t4_20.predict(x_test_t_20_tfidf)

create_con_mat(lr_t4_20_y_pred, y_test_t4_20, [0,1,2,3], "Confusion Matrix
```

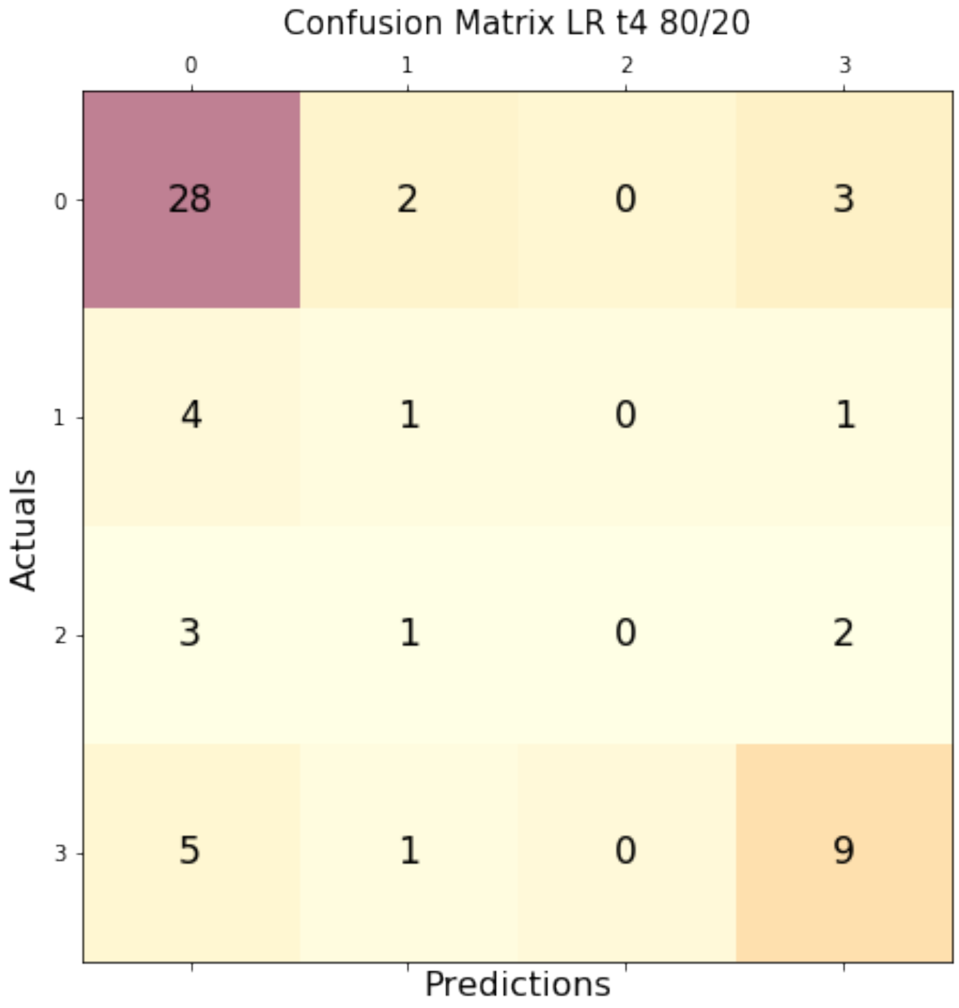

```
1 print(classification_report(y_test_t4_20, list(lr_t4_20_y_pred), labels=[0,
```

```
              precision    recall  f1-score   support

           0       0.70      0.85      0.77        33
           1       0.20      0.17      0.18         6
           2       0.00      0.00      0.00         6
           3       0.60      0.60      0.60        15

    accuracy                           0.63        60
   macro avg       0.38      0.40      0.39        60
weighted avg       0.55      0.63      0.59        60

/usr/local/lib/python3.7/site-packages/sklearn/metrics/_classification.py:1
  _warn_prf(average, modifier, msg_start, len(result))
/usr/local/lib/python3.7/site-packages/sklearn/metrics/_classification.py:1
  _warn_prf(average, modifier, msg_start, len(result))
/usr/local/lib/python3.7/site-packages/sklearn/metrics/_classification.py:1
  _warn_prf(average, modifier, msg_start, len(result))
```

```
classifier_lr_t2_20 = LogisticRegression(class_weight='balanced')
classifier_lr_t2_20.fit(x_train_t_20_tfidf, y_train_t2_20)

lr_t2_20_y_pred = classifier_lr_t2_20.predict(x_test_t_20_tfidf)

create_con_mat(lr_t2_20_y_pred, y_test_t2_20, [0,1], "Confusion Matrix LR t
```

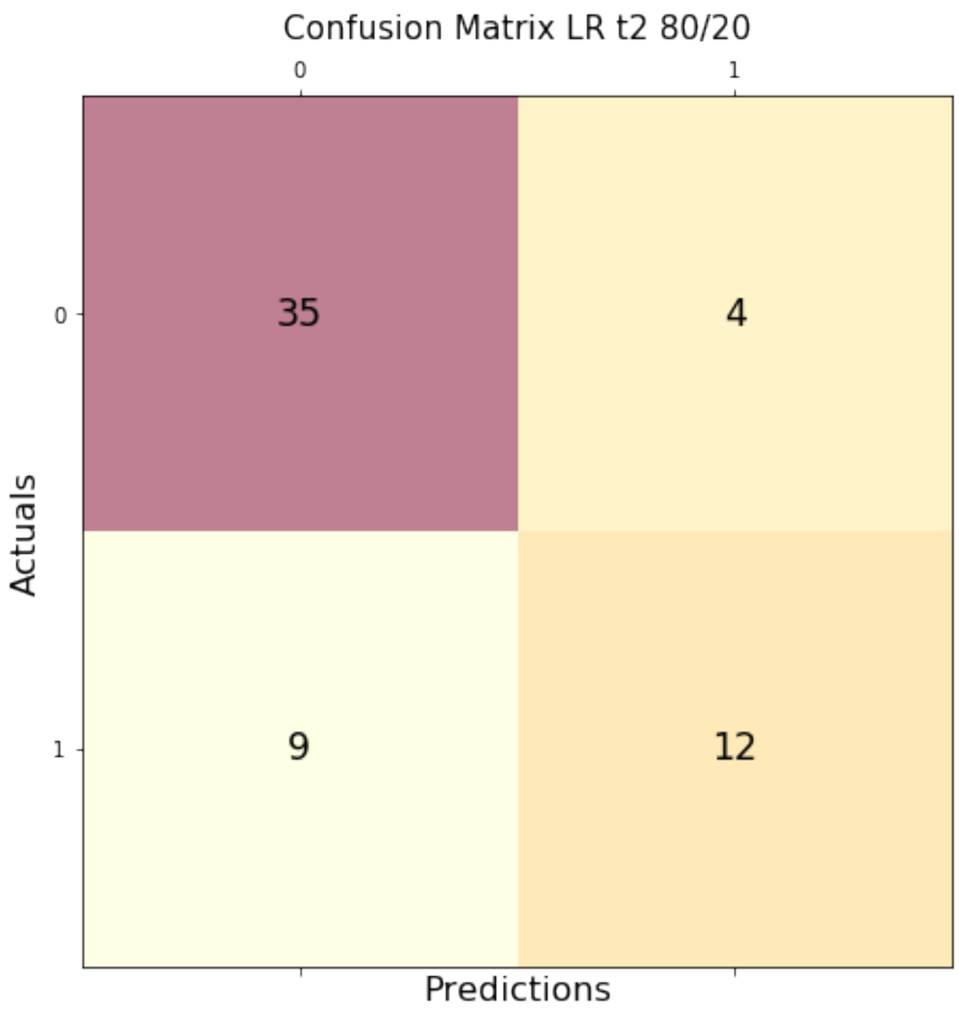


```
print(classification_report(y_test_t2_20, list(lr_t2_20_y_pred), labels=[0,
```

```
              precision    recall  f1-score   support

           0       0.80      0.90      0.84        39
           1       0.75      0.57      0.65        21

    accuracy                           0.78        60
   macro avg       0.77      0.73      0.75        60
weighted avg       0.78      0.78      0.78        60
```

```
#SVM t4 80/20
classifier_SVM_t4_20 = SVC(kernel='rbf', class_weight='balanced')  # Use ba
classifier_SVM_t4_20.fit(x_train_t_20_tfidf, y_train_t4_20)

# Prediction and Evaluation
SVM_t4_20_y_pred = classifier_SVM_t4_20.predict(x_test_t_20_tfidf)

#confusion matrix SVM s4 80/20
create_con_mat(SVM_t4_20_y_pred, y_test_t4_20, [0,1,2,3], "Confusion Matrix
```

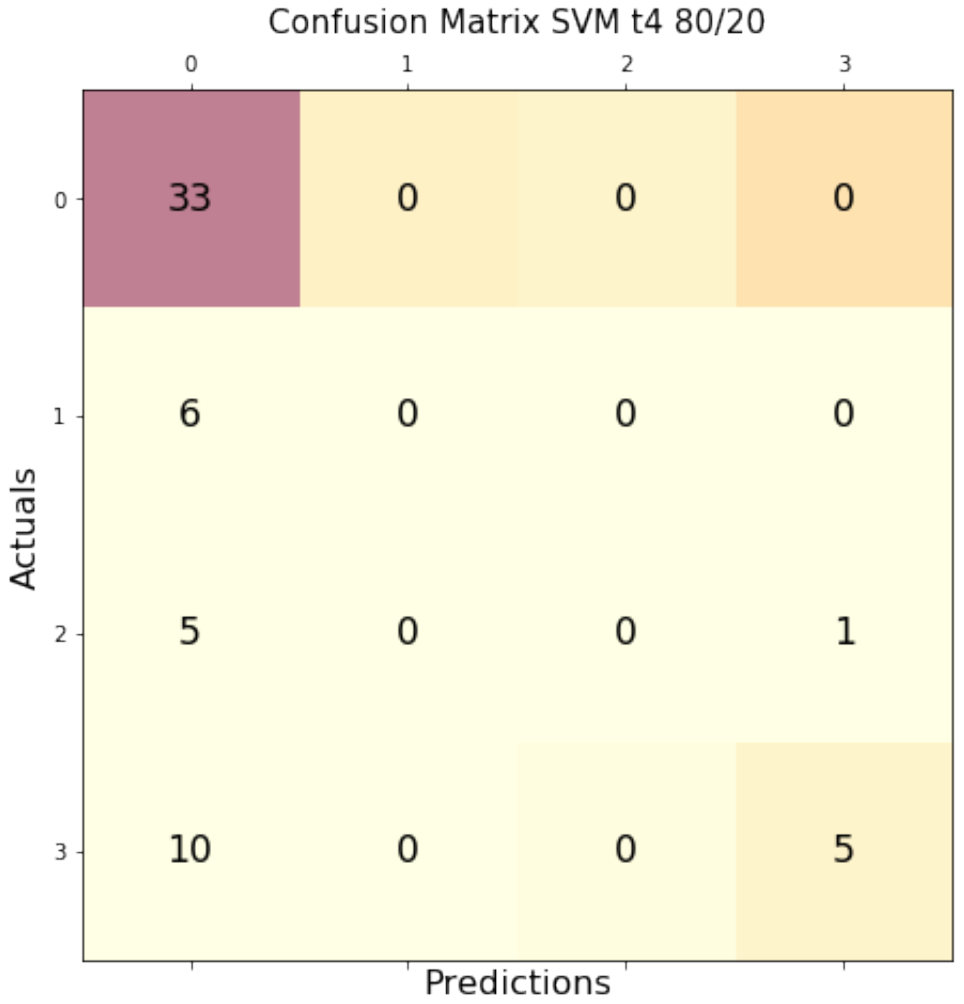

```
1 print(classification_report(y_test_t4_20, list(SVM_t4_20_y_pred), labels=[0
```

```
              precision    recall  f1-score   support

           0       0.61      1.00      0.76        33
           1       0.00      0.00      0.00         6
           2       0.00      0.00      0.00         6
           3       0.83      0.33      0.48        15

    accuracy                           0.63        60
   macro avg       0.36      0.33      0.31        60
weighted avg       0.54      0.63      0.54        60

/usr/local/lib/python3.7/site-packages/sklearn/metrics/_classification.py:1
  _warn_prf(average, modifier, msg_start, len(result))
/usr/local/lib/python3.7/site-packages/sklearn/metrics/_classification.py:1
  _warn_prf(average, modifier, msg_start, len(result))
/usr/local/lib/python3.7/site-packages/sklearn/metrics/_classification.py:1
  _warn_prf(average, modifier, msg_start, len(result))
```

```
#SVM t2 80/20
classifier_SVM_t2_20 = SVC(kernel='rbf', class_weight='balanced')  # Use ba
classifier_SVM_t2_20.fit(x_train_t_20_tfidf, y_train_t2_20)

# Prediction and Evaluation
SVM_t2_20_y_pred = classifier_SVM_t2_20.predict(x_test_t_20_tfidf)

#confusion matrix SVM s4 80/20
create_con_mat(SVM_t2_20_y_pred, y_test_t2_20, [0,1], "Confusion Matrix SVM
```

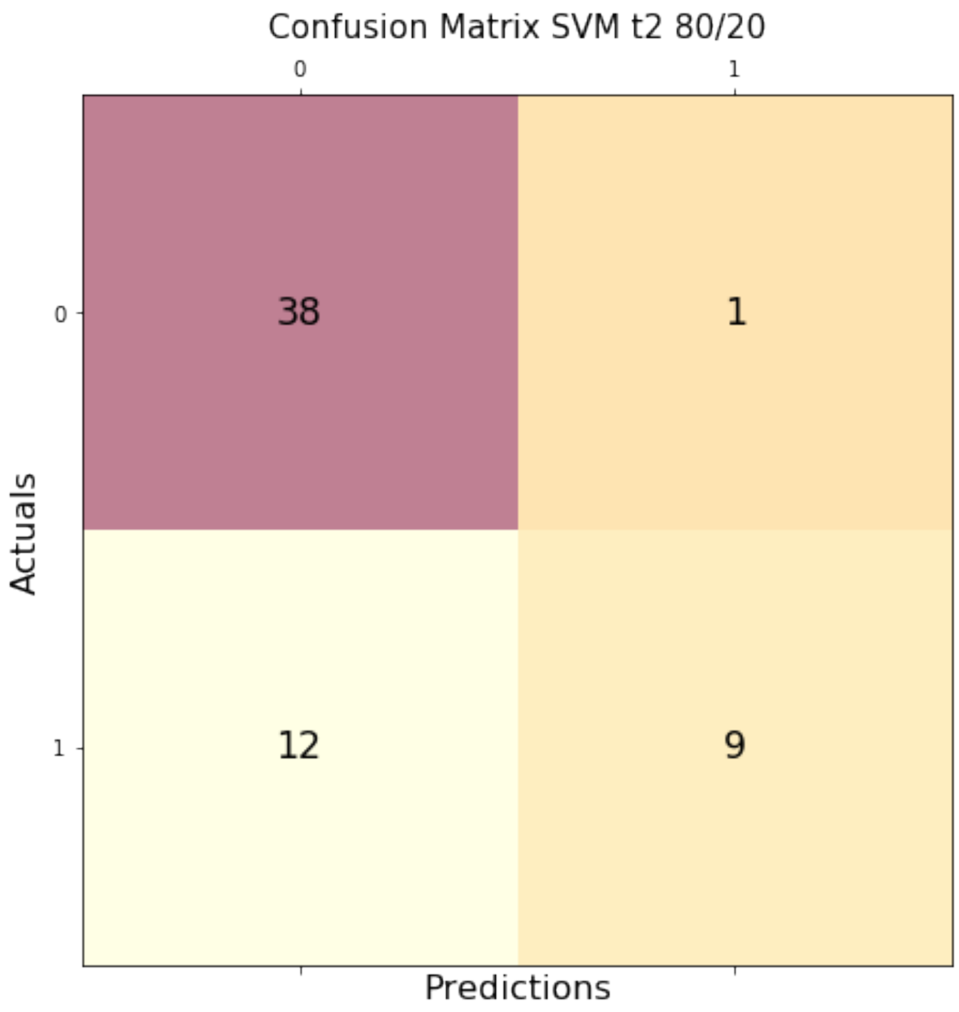


```
print(classification_report(y_test_t2_20, list(SVM_t2_20_y_pred), labels=[0
```

```
              precision    recall  f1-score   support

           0       0.76      0.97      0.85        39
           1       0.90      0.43      0.58        21

    accuracy                           0.78        60
   macro avg       0.83      0.70      0.72        60
weighted avg       0.81      0.78      0.76        60
```

```
#teacher 75/25 tfidf

tfidf_vectorizer = TfidfVectorizer()
x_train_t_25_tfidf = tfidf_vectorizer.fit_transform(x_train_t_25)
x_test_t_25_tfidf = tfidf_vectorizer.transform(x_test_t_25)

feature_names_tfidf = tfidf_vectorizer.get_feature_names_out()
```

```
classifier_lr_t4_25 = LogisticRegression(class_weight='balanced')
classifier_lr_t4_25.fit(x_train_t_25_tfidf, y_train_t4_25)

lr_t4_25_y_pred = classifier_lr_t4_25.predict(x_test_t_25_tfidf)

create_con_mat(lr_t4_25_y_pred, y_test_t4_25, [0,1,2,3], "Confusion Matrix
```

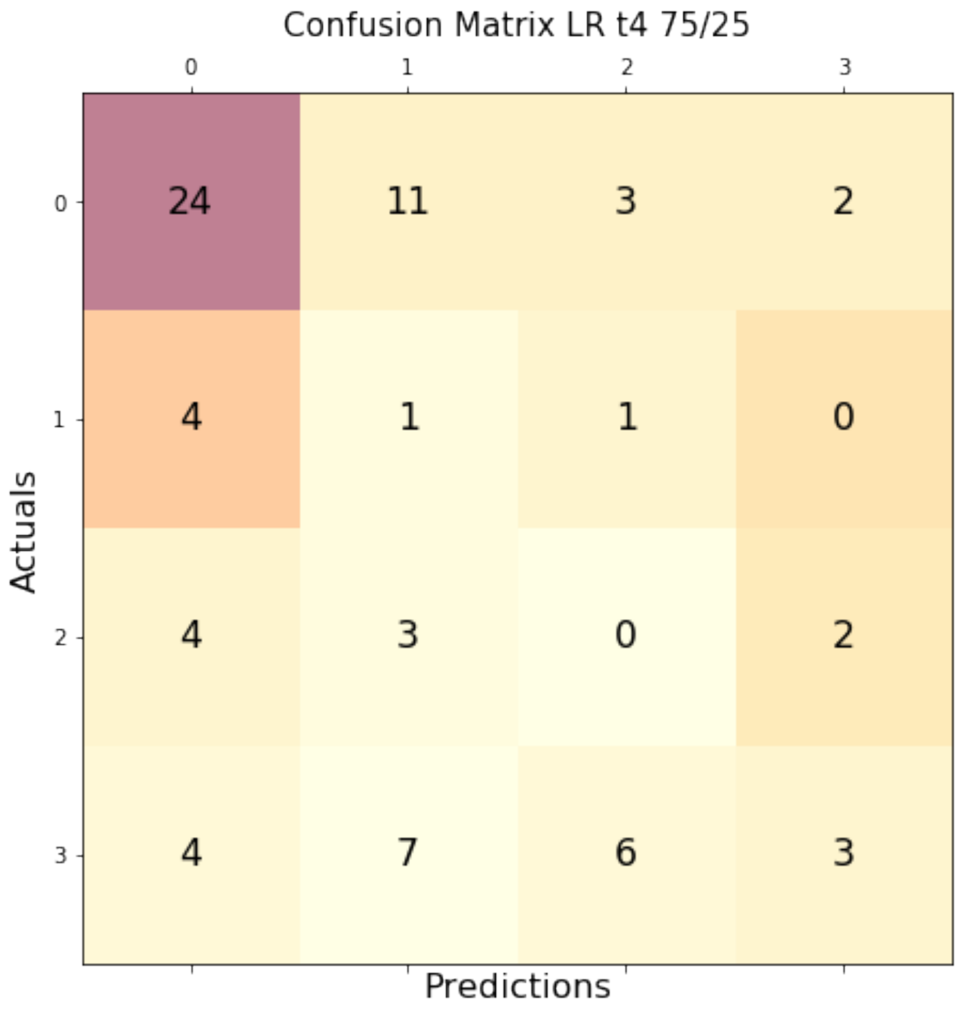

```
print(classification_report(y_test_t4_25, list(lr_t4_25_y_pred), labels=[0,
```

```
              precision    recall  f1-score   support

           0       0.67      0.60      0.63        40
           1       0.05      0.17      0.07         6
           2       0.00      0.00      0.00         9
           3       0.43      0.15      0.22        20

    accuracy                           0.37        75
   macro avg       0.29      0.23      0.23        75
weighted avg       0.47      0.37      0.40        75
```

```
classifier_lr_t2_25 = LogisticRegression(class_weight='balanced')
classifier_lr_t2_25.fit(x_train_t_25_tfidf, y_train_t2_25)

lr_t2_25_y_pred = classifier_lr_t2_25.predict(x_test_t_25_tfidf)

create_con_mat(lr_t2_25_y_pred, y_test_t2_25, [0,1], "Confusion Matrix LR t
```

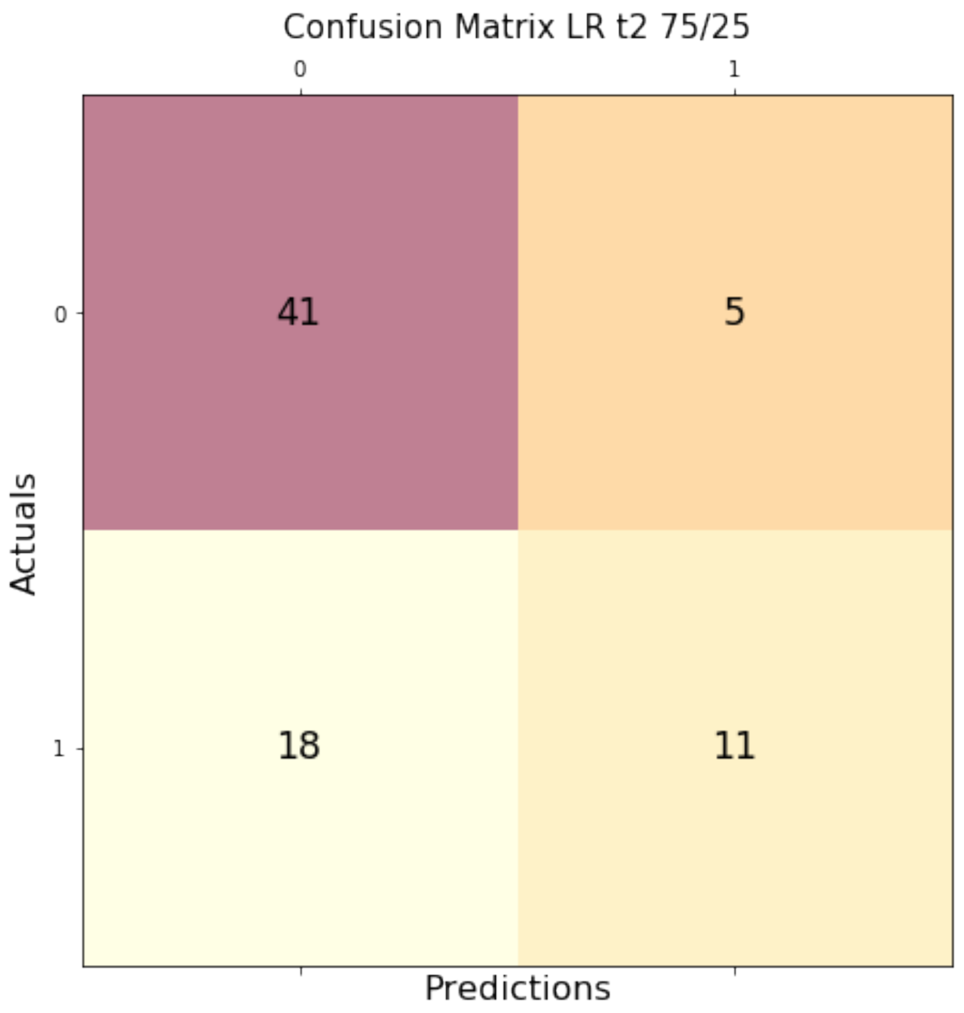

```
print(classification_report(y_test_t2_25, list(lr_t2_25_y_pred), labels=[0,
```

```
              precision    recall  f1-score   support

           0       0.69      0.89      0.78        46
           1       0.69      0.38      0.49        29

    accuracy                           0.69        75
   macro avg       0.69      0.64      0.63        75
weighted avg       0.69      0.69      0.67        75
```

```
#SVM t4 75/25
classifier_SVM_t4_25 = SVC(kernel='rbf', class_weight='balanced')  # Use ba
classifier_SVM_t4_25.fit(x_train_t_25_tfidf, y_train_t4_25)

# Prediction and Evaluation
SVM_t4_25_y_pred = classifier_SVM_t4_25.predict(x_test_t_25_tfidf)

#confusion matrix SVM t4 75/25
create_con_mat(SVM_t4_25_y_pred, y_test_t4_25, [0,1,2,3], "Confusion Matrix
```

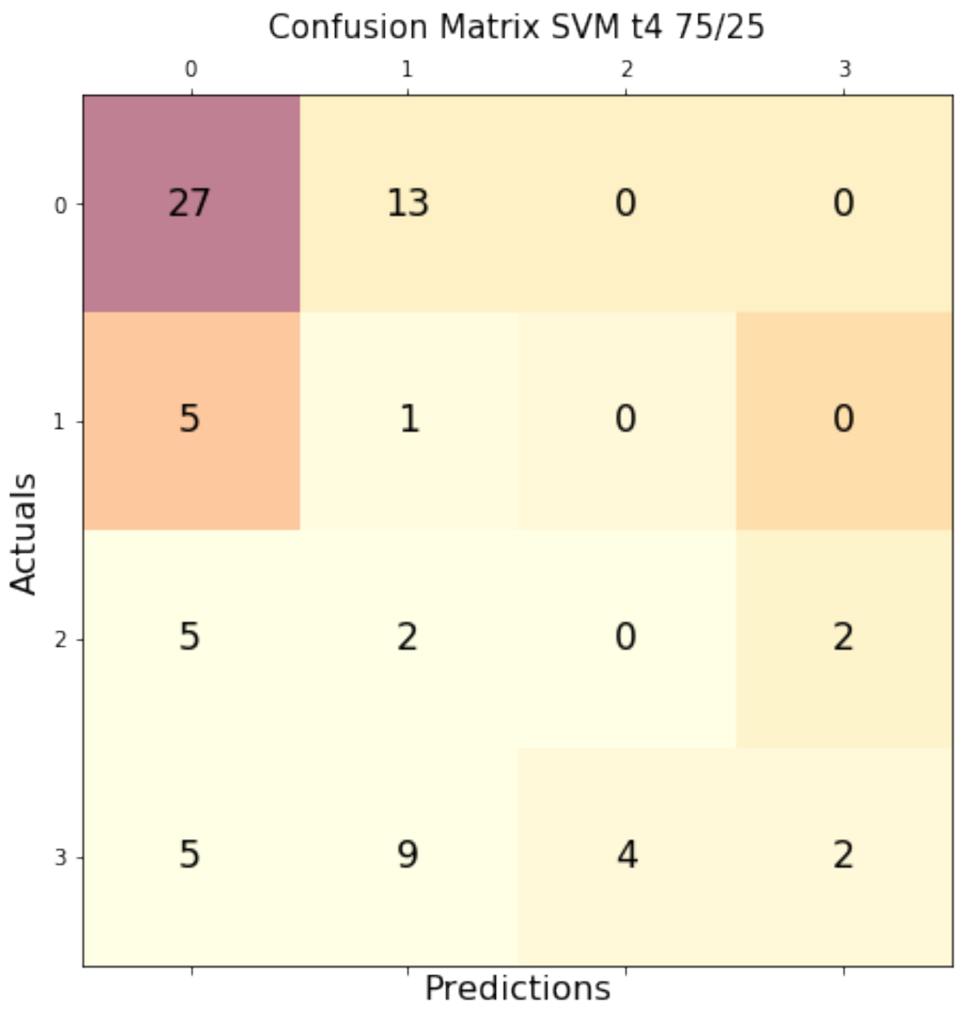

```
print(classification_report(y_test_t4_25, list(SVM_t4_25_y_pred), labels=[0
```

```
              precision    recall  f1-score   support

           0       0.64      0.68      0.66        40
           1       0.04      0.17      0.06         6
           2       0.00      0.00      0.00         9
           3       0.50      0.10      0.17        20

    accuracy                           0.40        75
   macro avg       0.30      0.24      0.22        75
weighted avg       0.48      0.40      0.40        75
```

```
#SVM t2 75/25
classifier_SVM_t2_25 = SVC(kernel='rbf', class_weight='balanced')  # Use ba
classifier_SVM_t2_25.fit(x_train_t_25_tfidf, y_train_t2_25)

# Prediction and Evaluation
SVM_t2_25_y_pred = classifier_SVM_t2_25.predict(x_test_t_25_tfidf)

#confusion matrix SVM t2 75/25
create_con_mat(SVM_t2_25_y_pred, y_test_t2_25, [0,1], "Confusion Matrix SVM
```

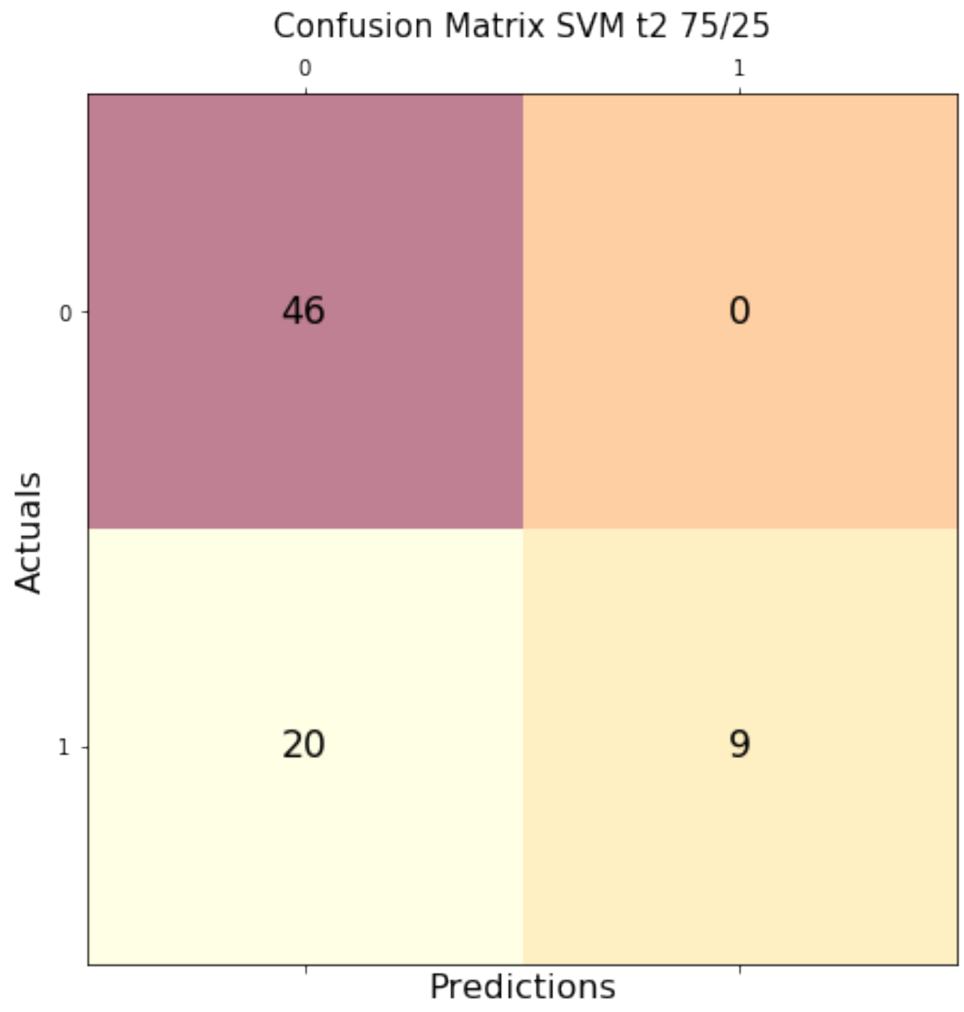

```
1 print(classification_report(y_test_t2_25, list(SVM_t2_25_y_pred), labels=[0
```

```
              precision    recall  f1-score   support

           0       0.70      1.00      0.82        46
           1       1.00      0.31      0.47        29

    accuracy                           0.73        75
   macro avg       0.85      0.66      0.65        75
weighted avg       0.81      0.73      0.69        75
```

Part III

Bert

```
import os
os.environ["CUDA_LAUNCH_BLOCKING"] = "1"
```

```
!pip install transformers==4.40.2
```

顯示隱藏的輸出內容

```
!pip install simpletransformers
```

顯示隱藏的輸出內容

```
from simpletransformers.classification import ClassificationModel, Classific
import pandas as pd
import logging
import csv

logging.basicConfig(level=logging.INFO)
transformers_logger = logging.getLogger("transformers")
transformers_logger.setLevel(logging.WARNING)
```

```
# upload data
from google.colab import drive
drive.mount('/content/drive')
```

```
Mounted at /content/drive
```

student 90/10

```
# Preparing train/test data for 90/10 students' records
train_s4_10 = []
train_s2_10 = []
test_s4_10 = []
test_s2_10 = []
x_test_s_10 = []
y_test_s4_10 = []
y_test_s2_10 = []

with open('/content/drive/MyDrive/HE/train_s_10.csv', newline='', encoding=
    rows = csv.reader(f)
    for row in rows:
        t4 = []
        t4.append(str(row[0]))
        t4.append(int(row[1]))
        train_s4_10.append(t4)
        t2 = []
        t2.append(str(row[0]))
        t2.append(int(row[2]))
        train_s2_10.append(t2)

with open('/content/drive/MyDrive/HE/test_s_10.csv', newline='', encoding="
    rows = csv.reader(f)
    for row in rows:
        count+=1
        t4 = []
        t4.append(str(row[0]))
        t4.append(int(row[1]))
        test_s4_10.append(t4)
        x_test_s_10.append(t4[0])
        y_test_s4_10.append(t4[1])
        t2 = []
        t2.append(str(row[0]))
        t2.append(int(row[2]))
        test_s2_10.append(t2)
        y_test_s2_10.append(t2[1])


train_df_s4_10 = pd.DataFrame(train_s4_10)
train_df_s4_10.columns = ["text", "labels"]

train_df_s2_10 = pd.DataFrame(train_s2_10)
train_df_s2_10.columns = ["text", "labels"]

eval_df_s4_10 = pd.DataFrame(test_s4_10)
eval_df_s4_10.columns = ["text", "labels"]

eval_df_s2_10 = pd.DataFrame(test_s2_10)
eval_df_s2_10.columns = ["text", "labels"]
```

```
# Optional model (Teacher_4class) configuration
model_args = ClassificationArgs(sliding_window=True)
model_args.num_train_epochs = 10
model_args.max_seq_length = 512
model_args.best_model_dir = '/content/drive/MyDrive/HE/outputS4_s/bestModel
model_args.output_dir = '/content/drive/MyDrive/HE/outputS4_s'

# Create a ClassificationModel
model_S4_s = ClassificationModel(
    'bert',
    # 可換成訓練過的model
    'bert-base-multilingual-uncased',
    num_labels=4,
    use_cuda=True,
    args=model_args
)
```

Downloading: 100% 625/625 [00:00<00:00, 28.1kB/s]

Downloading: 100% 672M/672M [00:09<00:00, 70.8MB/s]

```
Some weights of the model checkpoint at bert-base-multilingual-uncased were
- This IS expected if you are initializing BertForSequenceClassification fr
- This IS NOT expected if you are initializing BertForSequenceClassificatio
Some weights of BertForSequenceClassification were not initialized from the
You should probably TRAIN this model on a down-stream task to be able to us
```

Downloading: 100% 28.0/28.0 [00:00<00:00, 1.89kB/s]

Downloading: 100% 872k/872k [00:00<00:00, 1.69MB/s]

```
model_S4_s.train_model(train_df_s4_10)
```

```
100% 270/270 [00:00<00:00, 4.88it/s]
Epoch 10 of 10: 100% 10/10 [04:31<00:00, 27.60s/it]
Epochs 0/10. Running Loss: 1.1724: 100% 34/34 [00:17<00:00, 2.44it/s]
Epochs 1/10. Running Loss: 0.6975: 100% 34/34 [00:16<00:00, 2.06it/s]
Epochs 2/10. Running Loss: 0.1835: 100% 34/34 [00:17<00:00, 1.71it/s]
Epochs 3/10. Running Loss: 1.1389: 100% 34/34 [00:17<00:00, 2.02it/s]
Epochs 4/10. Running Loss: 0.4152: 100% 34/34 [00:20<00:00, 1.78it/s]
Epochs 5/10. Running Loss: 0.0358: 100% 34/34 [00:17<00:00, 1.95it/s]
```

```
result, model_outputs, wrong_predictions_S4_s = model_S4_s.eval_model(eval_
print(result)
print(len(wrong_predictions_S4_s))
```

```
100% 30/30 [00:00<00:00, 7.95it/s]
Running Evaluation: 100% 4/4 [00:00<00:00, 8.12it/s]
{'mcc': 0.4034460957307521, 'eval_loss': 2.032183051109314}
11
```

```
predS4_s, outputsS4_s = model_S4_s.predict(x_test_s_10)
```

```
100% 30/30 [00:00<00:00, 6.38it/s]
100% 4/4 [00:00<00:00, 7.47it/s]
```

```
from sklearn.metrics import confusion_matrix
import matplotlib.pyplot as plt
```

```
# creates confusion matrix
mat_conS4 = (confusion_matrix(list(predS4_s), y_test_s4_10, labels=[0,1,2,3

# Setting the attributes
fig, px = plt.subplots(figsize=(7.5, 7.5))
px.matshow(mat_conS4, cmap=plt.cm.YlOrRd, alpha=0.5)
for m in range(mat_conS4.shape[0]):
    for n in range(mat_conS4.shape[1]):
        px.text(x=m,y=n,s=mat_conS4[m, n], va='center', ha='center', size='

# Sets the labels
plt.xlabel('Predictions', fontsize=16)
plt.ylabel('Actuals', fontsize=16)
plt.title('Confusion Matrix', fontsize=15)
plt.show()
```

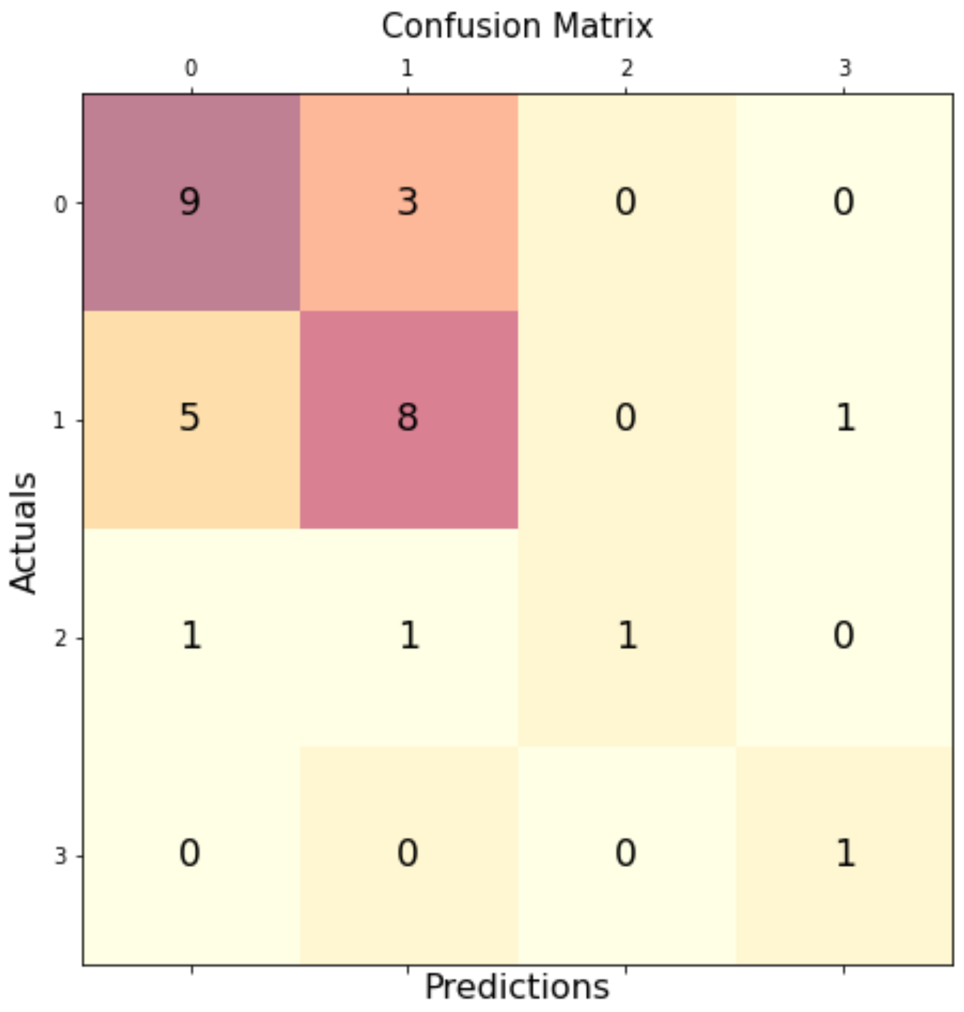

```
from sklearn.metrics import classification_report
print(classification_report(y_test_s4_10, list(predS4_s), labels=[0,1, 2, 3
```

```
              precision    recall  f1-score   support

           0       0.60      0.75      0.67        12
           1       0.67      0.57      0.62        14
           2       1.00      0.33      0.50         3
           3       0.50      1.00      0.67         1

    accuracy                           0.63        30
   macro avg       0.69      0.66      0.61        30
weighted avg       0.67      0.63      0.63        30
```

```
#import torch

# Optional model(student_2class) configuration
model_args = ClassificationArgs(sliding_window=True)
model_args.num_train_epochs = 10
model_args.max_seq_length = 512
model_args.best_model_dir = '/content/drive/MyDrive/HE/outputs2_s/bestModel'
model_args.output_dir = '/content/drive/MyDrive/HE/outputs2_s'

#cuda_available = torch.cuda.is_available()
# Create a ClassificationModel
model_S2_s = ClassificationModel(
    'bert',
    # 可換成訓練過的model
    'bert-base-multilingual-uncased',
    num_labels=2,
    use_cuda=True,
    args=model_args
)
```

```
Some weights of the model checkpoint at bert-base-multilingual-uncased were
- This IS expected if you are initializing BertForSequenceClassification fr
- This IS NOT expected if you are initializing BertForSequenceClassificatio
Some weights of BertForSequenceClassification were not initialized from the
You should probably TRAIN this model on a down-stream task to be able to us
```

```
model_S2_s.train_model(train_df_s2_10)
```

顯示隱藏的輸出內容

```
result, model_outputs, wrong_predictions_S2_s = model_S2_s.eval_model(eval_
print(result)
print(len(wrong_predictions_S2_s))
```

顯示隱藏的輸出內容

```
predS2_s, outputsS2_s = model_S2_s.predict(x_test_s_10)
```

顯示隱藏的輸出內容

```
# creates confusion matrix
mat_conS2 = (confusion_matrix(list(predS2_s), y_test_s2_10, labels=[0,1]))

# Setting the attributes
fig, px = plt.subplots(figsize=(7.5, 7.5))
px.matshow(mat_conS2, cmap=plt.cm.YlOrRd, alpha=0.5)
for m in range(mat_conS2.shape[0]):
    for n in range(mat_conS2.shape[1]):
        px.text(x=m,y=n,s=mat_conS2[m, n], va='center', ha='center', size='

# Sets the labels
plt.xlabel('Predictions', fontsize=16)
plt.ylabel('Actuals', fontsize=16)
plt.title('Confusion Matrix', fontsize=15)
plt.show()
```

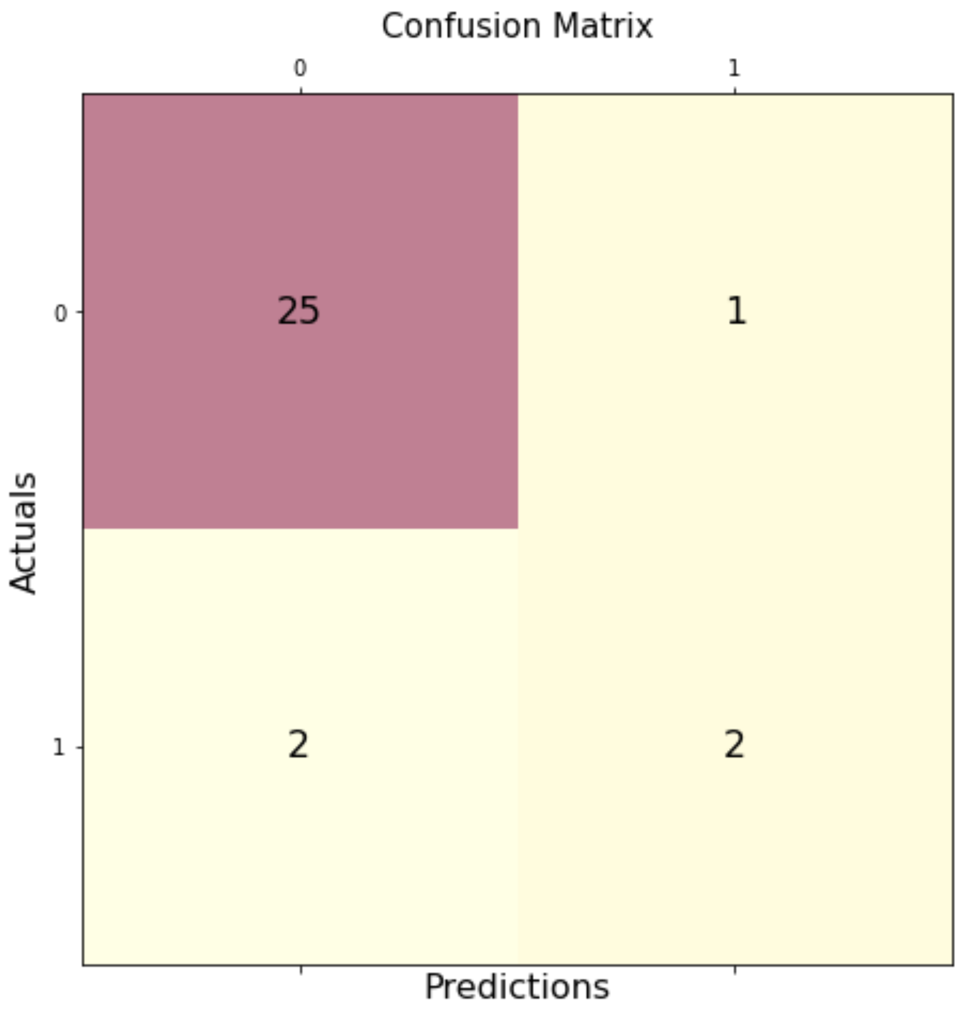

Teacher 90/10

```
# Preparing train data for 90/10 teachers' records
train_t4_10 = []
train_t2_10 = []
```

```
test_t4_10 = []
test_t2_10 = []
x_test_t_10 = []
y_test_t4_10 = []
y_test_t2_10 = []

with open('/content/drive/MyDrive/HE/train_t_10.csv', newline='', encoding=
    rows = csv.reader(f)
    for row in rows:
        t4 = []
        t4.append(str(row[0]))
        t4.append(int(row[1]))
        train_t4_10.append(t4)
        t2 = []
        t2.append(str(row[0]))
        t2.append(int(row[2]))
        train_t2_20.append(t2)

with open('/content/drive/MyDrive/HE/test_t_10.csv', newline='', encoding="
    rows = csv.reader(f)
    for row in rows:
        count+=1
        t4 = []
        t4.append(str(row[0]))
        t4.append(int(row[1]))
        test_t4_10.append(t4)
        x_test_t_10.append(t4[0])
        y_test_t4_10.append(t4[1])
        t2 = []
        t2.append(str(row[0]))
        t2.append(int(row[2]))
        test_t2_10.append(t2)
        y_test_t2_10.append(t2[1])


train_df_t4_10 = pd.DataFrame(train_t4_10)
train_df_t4_10.columns = ["text", "labels"]

train_df_t2_10 = pd.DataFrame(train_t2_10)
train_df_t2_10.columns = ["text", "labels"]

eval_df_t4_10 = pd.DataFrame(test_t4_10)
eval_df_t4_10.columns = ["text", "labels"]

eval_df_t2_10 = pd.DataFrame(test_t2_10)
eval_df_t2_10.columns = ["text", "labels"]
```

```
# Optional model (Teacher_4class) configuration
model_args = ClassificationArgs(sliding_window=True)
model_args.num_train_epochs = 10
model_args.max_seq_length = 512
model_args.best_model_dir = '/content/drive/MyDrive/HE/outputt4_s/bestModel
model_args.output_dir = '/content/drive/MyDrive/HE/outputt4_s'


# Create a ClassificationModel
model_T4_s = ClassificationModel(
    'bert',
    # 可換成訓練過的model
    'bert-base-multilingual-uncased',
    num_labels=4,
    use_cuda=True,
    args=model_args
)
```

顯示隱藏的輸出內容

```
model_T4_s.train_model(train_df_t4_10)
```

顯示隱藏的輸出內容

```
result, model_outputs_T4_s, wrong_predictions_T4_s = model_T4_s.eval_model(
print(result)
print(len(wrong_predictions_T4_s))
```

顯示隱藏的輸出內容

```
predT4_s, outputsT4_s = model_T4_s.predict(x_test_t_10)
```

顯示隱藏的輸出內容

```
# creates confusion matrix
mat_conT4 = (confusion_matrix(list(predT4_s), y_test_t4_10, labels=[0,1,2,3

# Setting the attributes
fig, px = plt.subplots(figsize=(7.5, 7.5))
px.matshow(mat_conT4, cmap=plt.cm.YlOrRd, alpha=0.5)
for m in range(mat_conT4.shape[0]):
    for n in range(mat_conT4.shape[1]):
        px.text(x=m,y=n,s=mat_conT4[m, n], va='center', ha='center', size='

# Sets the labels
plt.xlabel('Predictions', fontsize=16)
plt.ylabel('Actuals', fontsize=16)
plt.title('Confusion Matrix', fontsize=15)
plt.show()
```

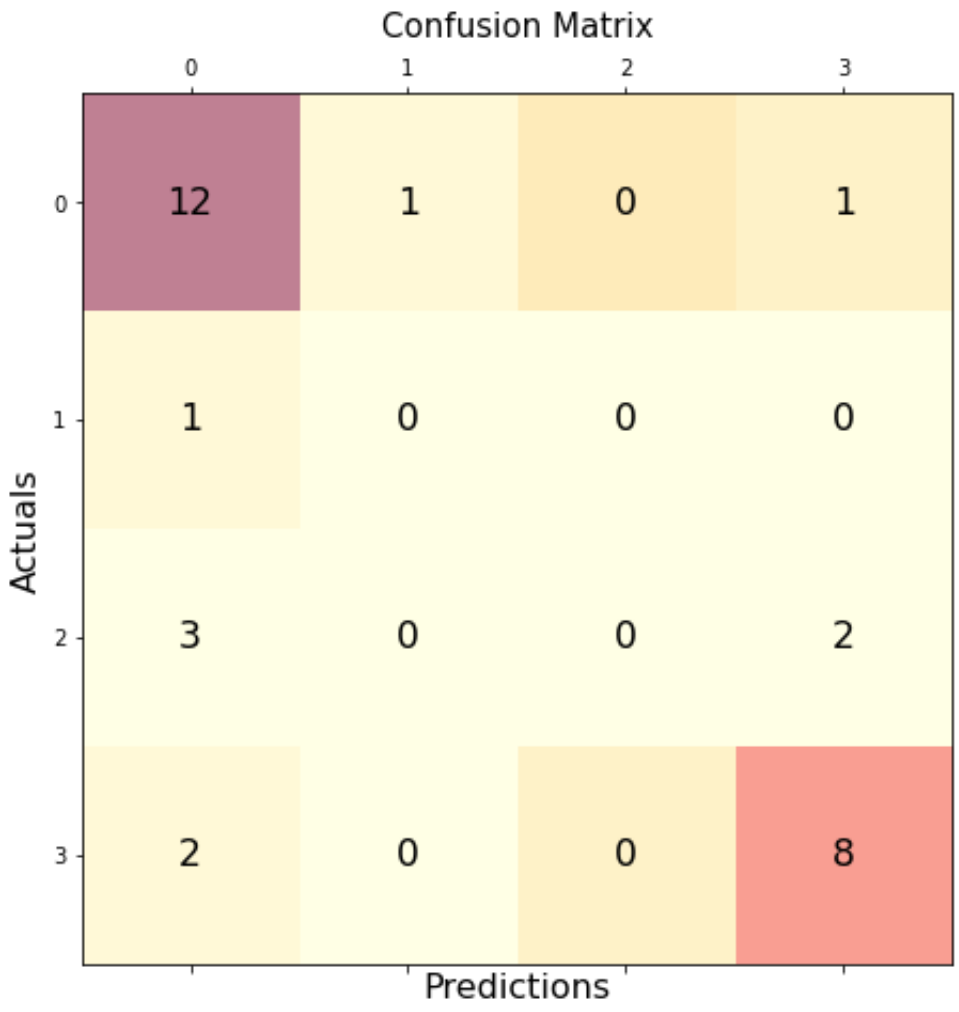

```
print(classification_report(y_test_t4_10, list(predT4_s), labels=[0,1, 2, 3
```

```
              precision    recall  f1-score   support

           0       0.67      0.86      0.75        14
           1       0.00      0.00      0.00         1
           2       0.00      0.00      0.00         5
           3       0.73      0.80      0.76        10

    accuracy                           0.67        30
   macro avg       0.35      0.41      0.38        30
weighted avg       0.55      0.67      0.60        30

/usr/local/lib/python3.8/dist-packages/sklearn/metrics/_classification.py:1
  _warn_prf(average, modifier, msg_start, len(result))
/usr/local/lib/python3.8/dist-packages/sklearn/metrics/_classification.py:1
  _warn_prf(average, modifier, msg_start, len(result))
/usr/local/lib/python3.8/dist-packages/sklearn/metrics/_classification.py:1
  _warn_prf(average, modifier, msg_start, len(result))
```

```
# Optional model (Teacher_2class) configuration
model_args = ClassificationArgs(sliding_window=True)
model_args.num_train_epochs = 10
model_args.max_seq_length = 512
model_args.best_model_dir = '/content/drive/MyDrive/HE/outputt2_s/bestModel
model_args.output_dir = '/content/drive/MyDrive/HE/outputt2_s'

# Create a ClassificationModel
model_T2_s = ClassificationModel(
    'bert',
    # 可換成訓練過的model
    'bert-base-multilingual-uncased',
    num_labels=2,
    use_cuda=True,
    args=model_args
)
```

顯示隱藏的輸出內容

```
model_T2_s.train_model(train_df_t2_10)
```

顯示隱藏的輸出內容

```
result, model_outputs, wrong_predictions_T2_s = model_T2_s.eval_model(eval_
print(result)
print(len(wrong_predictions_T2_s))
```

顯示隱藏的輸出內容

```
predT2_s, outputsT2_s = model_T2_s.predict(x_test_t_10)
```

顯示隱藏的輸出內容

```
# creates confusion matrix
mat_conT2 = (confusion_matrix(list(predT2_s), y_test_t4_10, labels=[0,1]))

# Setting the attributes
fig, px = plt.subplots(figsize=(7.5, 7.5))
px.matshow(mat_conT2, cmap=plt.cm.YlOrRd, alpha=0.5)
for m in range(mat_conT2.shape[0]):
    for n in range(mat_conT2.shape[1]):
        px.text(x=m,y=n,s=mat_conT2[m, n], va='center', ha='center', size='

# Sets the labels
plt.xlabel('Predictions', fontsize=16)
plt.ylabel('Actuals', fontsize=16)
plt.title('Confusion Matrix', fontsize=15)
plt.show()
```

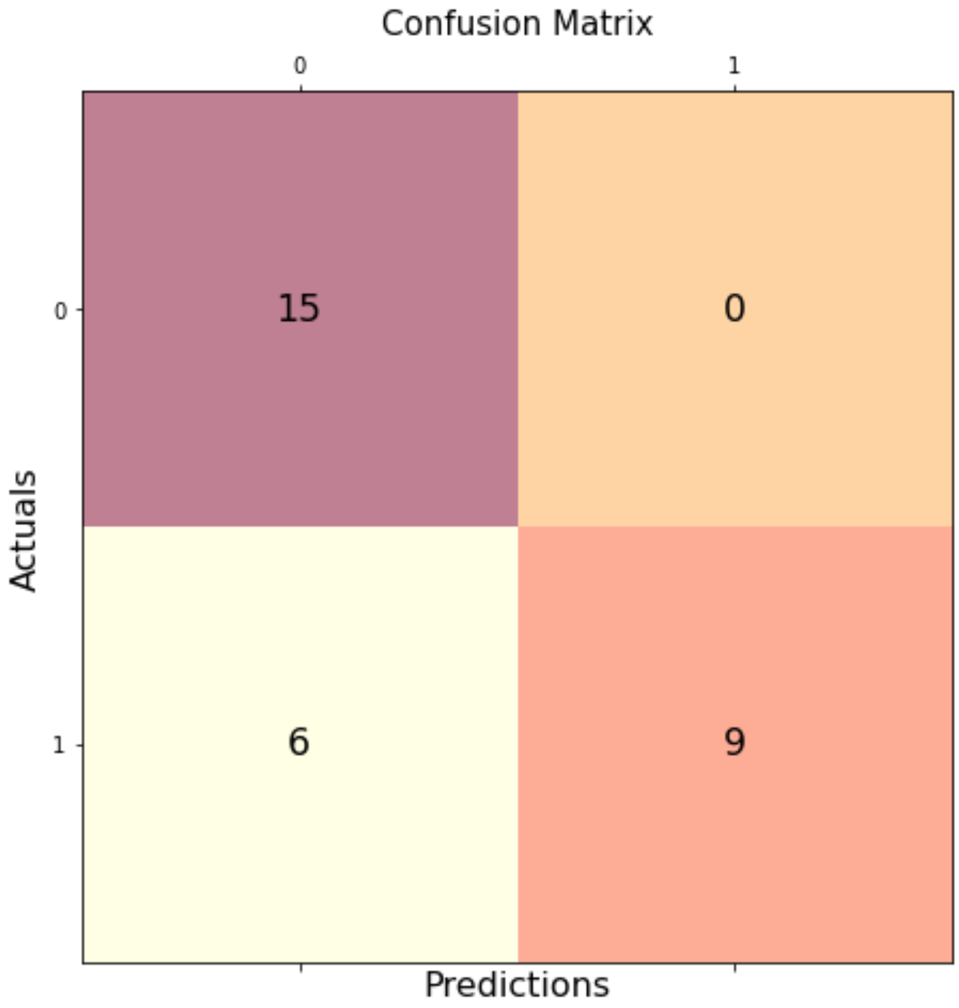

student 80/20

```
# Preparing train data for 80/20 students' records
train_s4_20 = []
train_s2_20 = []
test_s4_20 = []
test_s2_20 = []
x_test_s_20 = []
y_test_s4_20 = []
y_test_s2_20 = []

with open('/content/drive/MyDrive/HE/train_s_20.csv', newline='', encoding="
    rows = csv.reader(f)
    for row in rows:
        t4 = []
        t4.append(str(row[0]))
        t4.append(int(row[1]))
        train_s4_20.append(t4)
        t2 = []
        t2.append(str(row[0]))
        t2.append(int(row[2]))
        train_s2_20.append(t2)

with open('/content/drive/MyDrive/HE/test_s_20.csv', newline='', encoding="u
    rows = csv.reader(f)
    for row in rows:
        count+=1
        t4 = []
        t4.append(str(row[0]))
        t4.append(int(row[1]))
        test_s4_20.append(t4)
        x_test_s_20.append(t4[0])
        y_test_s4_20.append(t4[1])
        t2 = []
        t2.append(str(row[0]))
        t2.append(int(row[2]))
        test_s2_20.append(t2)
        y_test_s2_20.append(t2[1])


train_df_s4_20 = pd.DataFrame(train_s4_20)
train_df_s4_20.columns = ["text", "labels"]

train_df_s2_20 = pd.DataFrame(train_s2_20)
train_df_s2_20.columns = ["text", "labels"]

eval_df_s4_20 = pd.DataFrame(test_s4_20)
eval_df_s4_20.columns = ["text", "labels"]

eval_df_s2_20 = pd.DataFrame(test_s2_20)
eval_df_s2_20.columns = ["text", "labels"]
```

```
#import torch

# Optional model (student_4class) configuration
model_args = ClassificationArgs(sliding_window=True)
model_args.num_train_epochs = 10
model_args.max_seq_length = 512
model_args.best_model_dir = '/content/drive/MyDrive/HE/outputs4_20/bestMode
model_args.output_dir = '/content/drive/MyDrive/HE/outputs4_20'

#cuda_available = torch.cuda.is_available()
# Create a ClassificationModel
model_s4_20 = ClassificationModel(
    'bert',
    # 可換成訓練過的model
    'bert-base-multilingual-uncased',
    num_labels=4,
    use_cuda=True,
    args=model_args
)
```

顯示隱藏的輸出內容

```
model_s4_20.train_model(train_df_s4_20)
```

顯示隱藏的輸出內容

```
pred_s4_20, outputs_s4_20 = model_s4_20.predict(x_test_s_20)
```

顯示隱藏的輸出內容

```
from sklearn.metrics import confusion_matrix
import matplotlib.pyplot as plt
```

```
# creates confusion matrix
def create_con_mat(pred, label, label_range, title):

    mat_con = (confusion_matrix(list(pred), label, labels=label_range))

# Setting the attributes
    fig, px = plt.subplots(figsize=(7.5, 7.5))
    px.matshow(mat_con, cmap=plt.cm.YlOrRd, alpha=0.5)
    for m in range(mat_con.shape[0]):
        for n in range(mat_con.shape[1]):
            px.text(x=m,y=n,s=mat_con[m, n], va='center', ha='center', size

# Sets the labels
    plt.xlabel('Predictions', fontsize=16)
    plt.ylabel('Actuals', fontsize=16)
    plt.title(title, fontsize=15)
    plt.show()
```

```
1 create_con_mat(pred_s4_20, y_test_s4_20, [0,1,2,3], "Confusion Matrix Bert
```

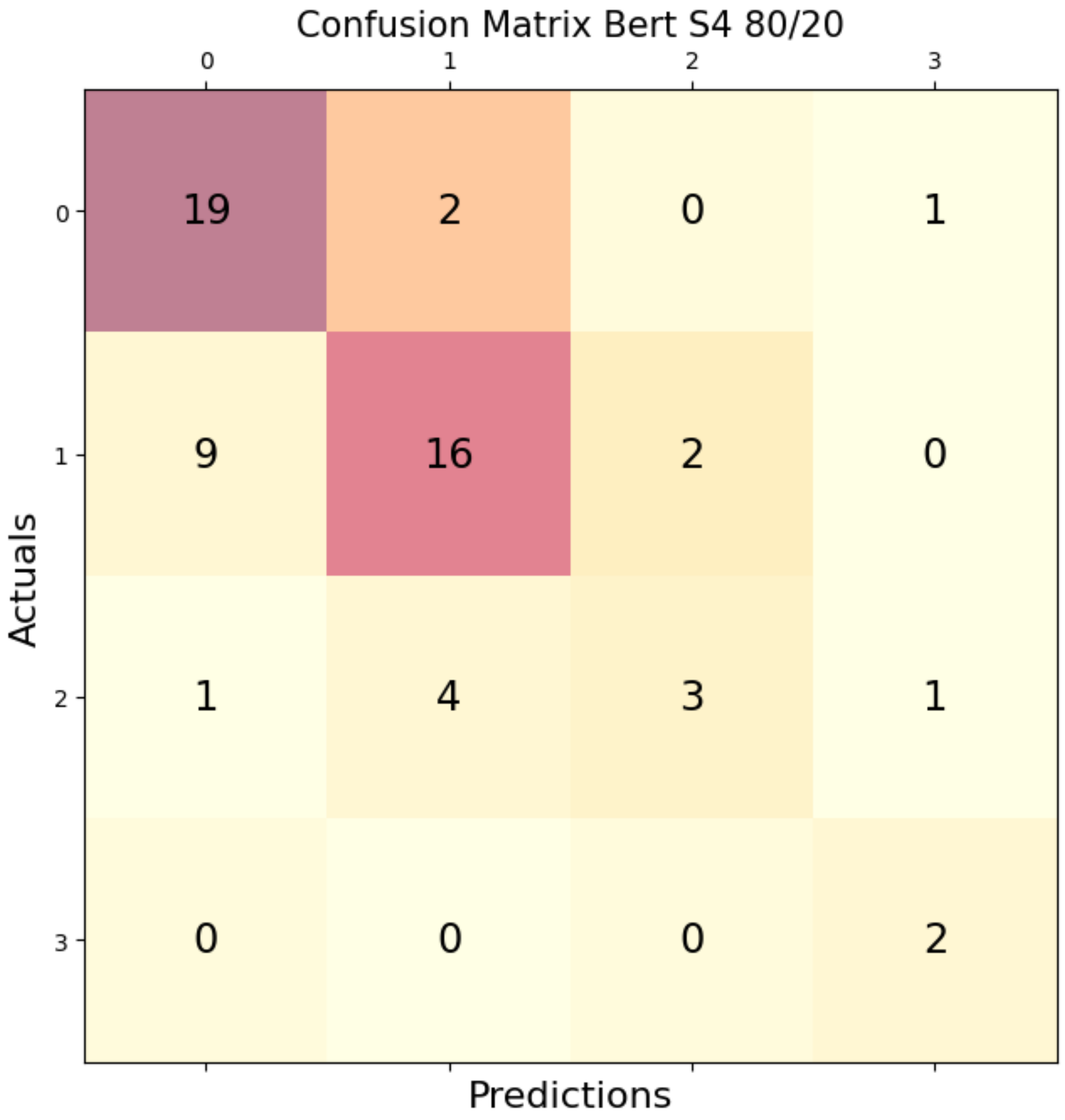

```
from sklearn.metrics import classification_report
print(classification_report(y_test_s4_20, list(pred_s4_20), labels=[0,1, 2,
```

```
              precision    recall  f1-score   support

           0       0.66      0.86      0.75        22
           1       0.73      0.59      0.65        27
           2       0.60      0.33      0.43         9
           3       0.50      1.00      0.67         2

    accuracy                           0.67        60
   macro avg       0.62      0.70      0.62        60
weighted avg       0.67      0.67      0.65        60
```

```
#import torch

# Optional model (student_4class) configuration
model_args = ClassificationArgs(sliding_window=True)
model_args.num_train_epochs = 10
model_args.max_seq_length = 512
model_args.best_model_dir = '/content/drive/MyDrive/HE/outputs2_20/bestModel
model_args.output_dir = '/content/drive/MyDrive/HE/outputs2_20'

#cuda_available = torch.cuda.is_available()
# Create a ClassificationModel
model_s2_20 = ClassificationModel(
    'bert',
    # 可換成訓練過的model
    'bert-base-multilingual-uncased',
    num_labels=2,
    use_cuda=True,
    args=model_args
)
```

顯示隱藏的輸出內容

```
model_s2_20.train_model(train_df_s2_20)
```

顯示隱藏的輸出內容

```
pred_s2_20, outputs_s2_20 = model_s2_20.predict(x_test_s_20)
```

顯示隱藏的輸出內容

```
create_con_mat(pred_s2_20, y_test_s2_20, [0,1], "Confusion Matrix Bert S2 8
```

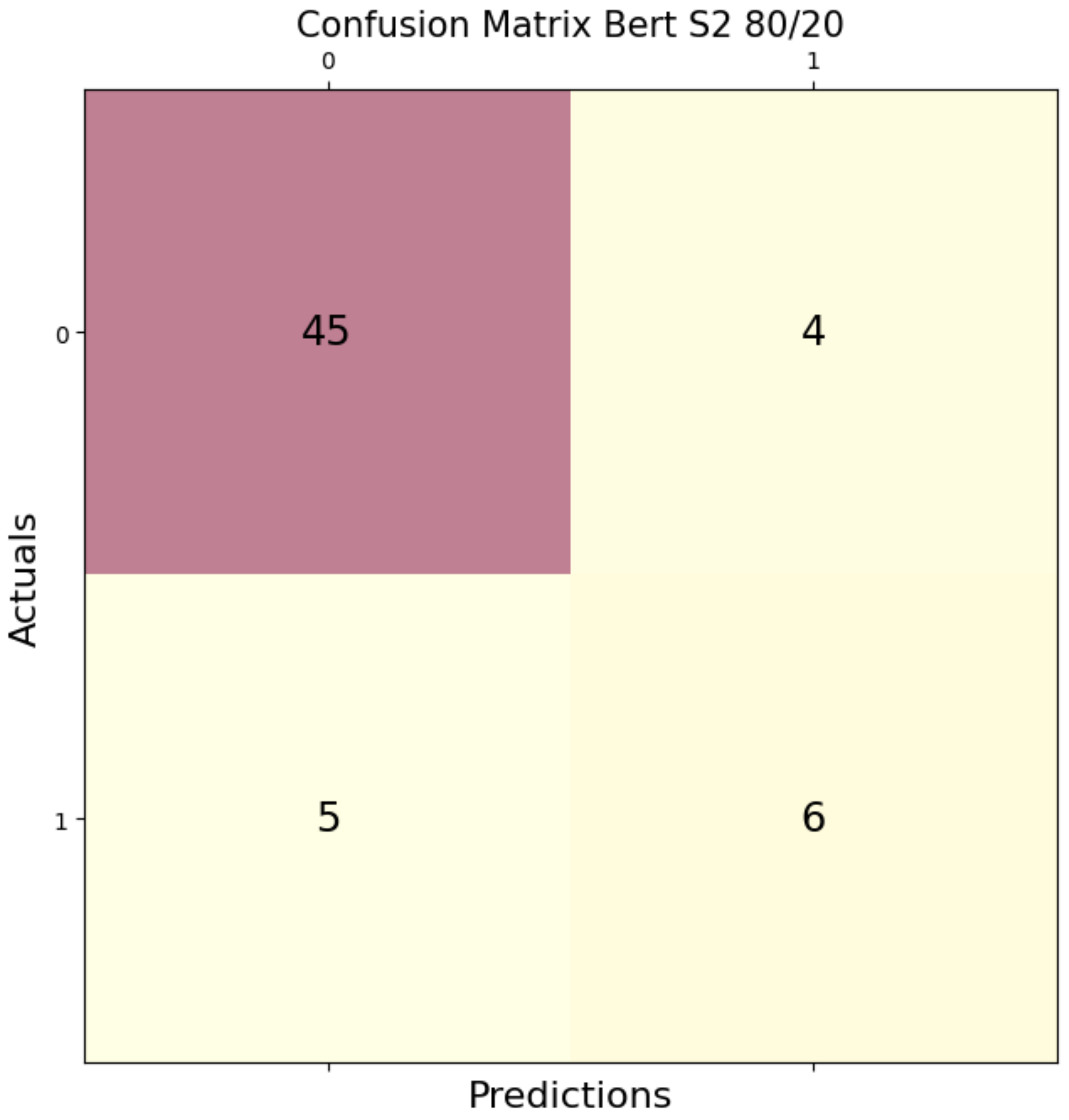

```
print(classification_report(y_test_s2_20, list(pred_s2_20), labels=[0,1]))
```

```
              precision    recall  f1-score   support

           0       0.90      0.92      0.91        49
           1       0.60      0.55      0.57        11

    accuracy                           0.85        60
   macro avg       0.75      0.73      0.74        60
weighted avg       0.85      0.85      0.85        60
```

student 75/25

```
# Preparing data for 75/25 students' records
train_s4_25 = []
train_s2_25 = []
test_s4_25 = []
test_s2_25 = []
x_test_s_25 = []
y_test_s4_25 = []
y_test_s2_25 = []

with open('/content/drive/MyDrive/HE/train_s_25.csv', newline='', encoding="
    rows = csv.reader(f)
    for row in rows:
        t4 = []
        t4.append(str(row[0]))
        t4.append(int(row[1]))
        train_s4_25.append(t4)
        t2 = []
        t2.append(str(row[0]))
        t2.append(int(row[2]))
        train_s2_25.append(t2)

with open('/content/drive/MyDrive/HE/test_s_25.csv', newline='', encoding="u
    rows = csv.reader(f)
    for row in rows:
        count+=1
        t4 = []
        t4.append(str(row[0]))
        t4.append(int(row[1]))
        test_s4_25.append(t4)
        x_test_s_25.append(t4[0])
        y_test_s4_25.append(t4[1])
        t2 = []
        t2.append(str(row[0]))
        t2.append(int(row[2]))
        test_s2_25.append(t2)
        y_test_s2_25.append(t2[1])


train_df_s4_25 = pd.DataFrame(train_s4_25)
train_df_s4_25.columns = ["text", "labels"]

train_df_s2_25 = pd.DataFrame(train_s2_25)
train_df_s2_25.columns = ["text", "labels"]

eval_df_s4_25 = pd.DataFrame(test_s4_25)
eval_df_s4_25.columns = ["text", "labels"]

eval_df_s2_25 = pd.DataFrame(test_s2_25)
eval_df_s2_25.columns = ["text", "labels"]
```

```
#import torch

# Optional model (student_4class) configuration
model_args = ClassificationArgs(sliding_window=True)
model_args.num_train_epochs = 10
model_args.max_seq_length = 512
model_args.best_model_dir = '/content/drive/MyDrive/HE/outputs4_25/bestModel
model_args.output_dir = '/content/drive/MyDrive/HE/outputs4_25'

#cuda_available = torch.cuda.is_available()
# Create a ClassificationModel
model_s4_25 = ClassificationModel(
    'bert',
    # 可換成訓練過的model
    'bert-base-multilingual-uncased',
    num_labels=4,
    use_cuda=True,
    args=model_args
)
```

顯示隱藏的輸出內容

```
model_s4_25.train_model(train_df_s4_25)
```

顯示隱藏的輸出內容

```
pred_s4_25, outputs_s4_25 = model_s4_25.predict(x_test_s_25)
```

顯示隱藏的輸出內容

```
1 create_con_mat(pred_s4_25, y_test_s4_25, [0,1,2,3], "Confusion Matrix Bert
```

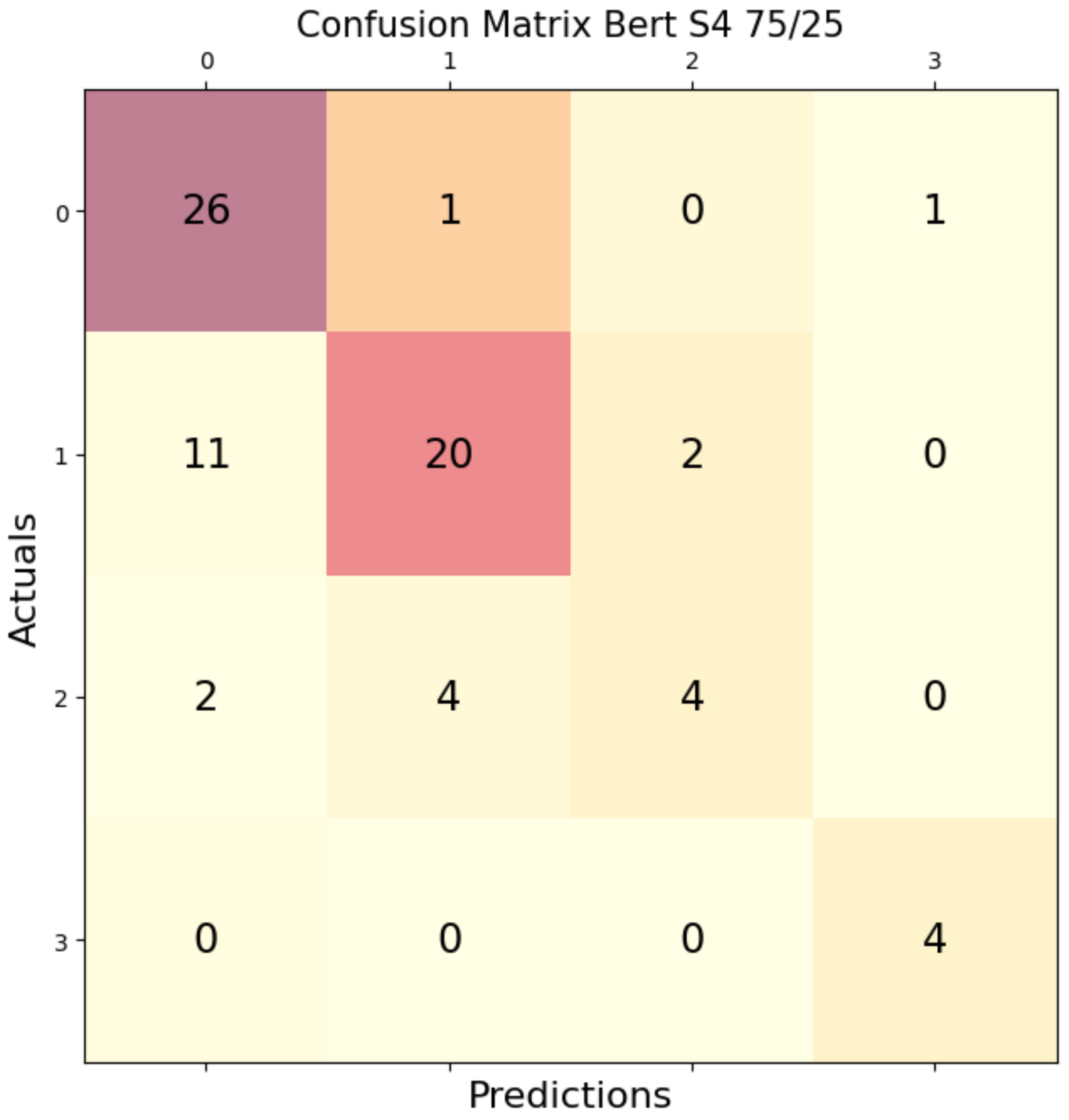

```
print(classification_report(y_test_s4_25, list(pred_s4_25), labels=[0,1,2,3
```

```
              precision    recall  f1-score   support

           0       0.67      0.93      0.78        28
           1       0.80      0.61      0.69        33
           2       0.67      0.40      0.50        10
           3       0.80      1.00      0.89         4

    accuracy                           0.72        75
   macro avg       0.73      0.73      0.71        75
weighted avg       0.73      0.72      0.71        75
```

```
#import torch

# Optional model (student_4class) configuration
model_args = ClassificationArgs(sliding_window=True)
model_args.num_train_epochs = 10
model_args.max_seq_length = 512
model_args.best_model_dir = '/content/drive/MyDrive/HE/outputs2_25/bestModel
model_args.output_dir = '/content/drive/MyDrive/HE/outputs2_25'

#cuda_available = torch.cuda.is_available()
# Create a ClassificationModel
model_s2_25 = ClassificationModel(
    'bert',
    # 可換成訓練過的model
    'bert-base-multilingual-uncased',
    num_labels=2,
    use_cuda=True,
    args=model_args
)
```

顯示隱藏的輸出內容

```
model_s2_25.train_model(train_df_s2_25)
```

顯示隱藏的輸出內容

```
pred_s2_25, outputs_s2_25 = model_s2_25.predict(x_test_s_25)
```

顯示隱藏的輸出內容

```
1 create_con_mat(pred_s2_25, y_test_s2_25, [0,1], "Confusion Matrix Bert S2 7
```

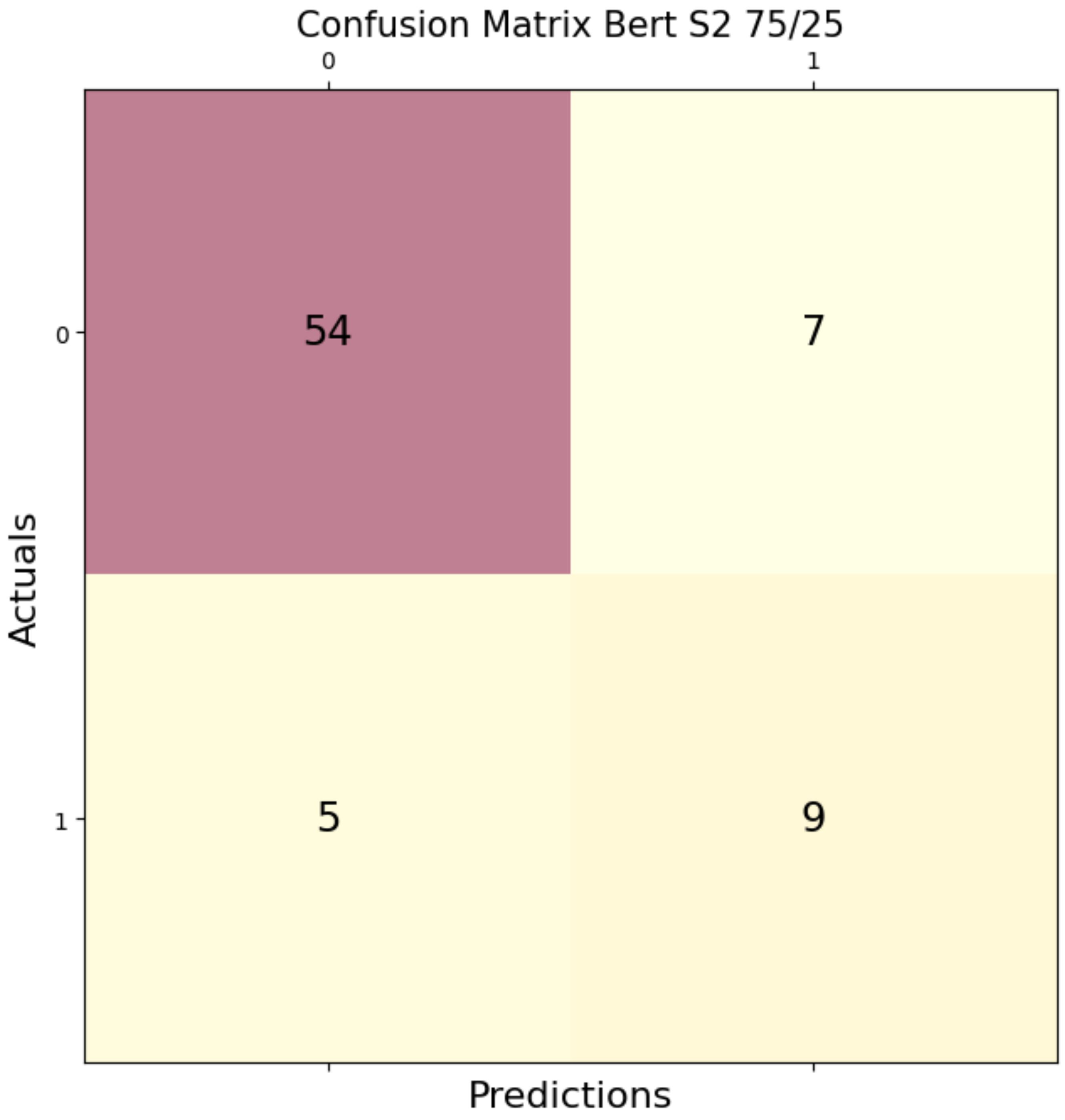

```
print(classification_report(y_test_s2_25, list(pred_s2_25), labels=[0,1]))
```

```
              precision    recall  f1-score   support

           0       0.92      0.89      0.90        61
           1       0.56      0.64      0.60        14

    accuracy                           0.84        75
   macro avg       0.74      0.76      0.75        75
weighted avg       0.85      0.84      0.84        75
```

Teacher 80/20

```
# Preparing data for 80/20 teachers' records
train_t4_20 = []
train_t2_20 = []
test_t4_20 = []
test_t2_20 = []
x_test_t_20 = []
y_test_t4_20 = []
y_test_t2_20 = []



with open('/content/drive/MyDrive/HE/train_t_20.csv', newline='', encoding="
    rows = csv.reader(f)
    for row in rows:
        t4 = []
        t4.append(str(row[0]))
        t4.append(int(row[1]))
        train_t4_20.append(t4)
        t2 = []
        t2.append(str(row[0]))
        t2.append(int(row[2]))
        train_t2_20.append(t2)


with open('/content/drive/MyDrive/HE/test_t_20.csv', newline='', encoding="u
    rows = csv.reader(f)
    for row in rows:
        count+=1
        t4 = []
        t4.append(str(row[0]))
        t4.append(int(row[1]))
        test_t4_20.append(t4)
        x_test_t_20.append(t4[0])
        y_test_t4_20.append(t4[1])
        t2 = []
        [illegible]
```

```
        t2.append(str(row[0]))
        t2.append(int(row[2]))
        test_t2_20.append(t2)
        y_test_t2_20.append(t2[1])


train_df_t4_20 = pd.DataFrame(train_t4_20)
train_df_t4_20.columns = ["text", "labels"]

train_df_t2_20 = pd.DataFrame(train_t2_20)
train_df_t2_20.columns = ["text", "labels"]

eval_df_t4_20 = pd.DataFrame(test_t4_20)
eval_df_t4_20.columns = ["text", "labels"]

eval_df_t2_20 = pd.DataFrame(test_t2_20)
eval_df_t2_20.columns = ["text", "labels"]
```

```
#import torch

# Optional model (student_4class) configuration
model_args = ClassificationArgs(sliding_window=True)
model_args.num_train_epochs = 10
model_args.max_seq_length = 512
model_args.best_model_dir = '/content/drive/MyDrive/HE/outputt4_20/bestMode
model_args.output_dir = '/content/drive/MyDrive/HE/outputt4_20'

#cuda_available = torch.cuda.is_available()
# Create a ClassificationModel
model_t4_20 = ClassificationModel(
    'bert',
    # 可換成訓練過的model
    'bert-base-multilingual-uncased',
    num_labels=4,
    use_cuda=True,
    args=model_args
)
```

顯示隱藏的輸出內容

```
model_t4_20.train_model(train_df_t4_20)
```

顯示隱藏的輸出內容

```
pred_t4_20, outputs_t4_20= model_t4_20.predict(x_test_t_20)
```

顯示隱藏的輸出內容

```
1 create_con_mat(pred_t4_20, y_test_t4_20, [0,1,2,3], "Confusion Matrix Bert
```

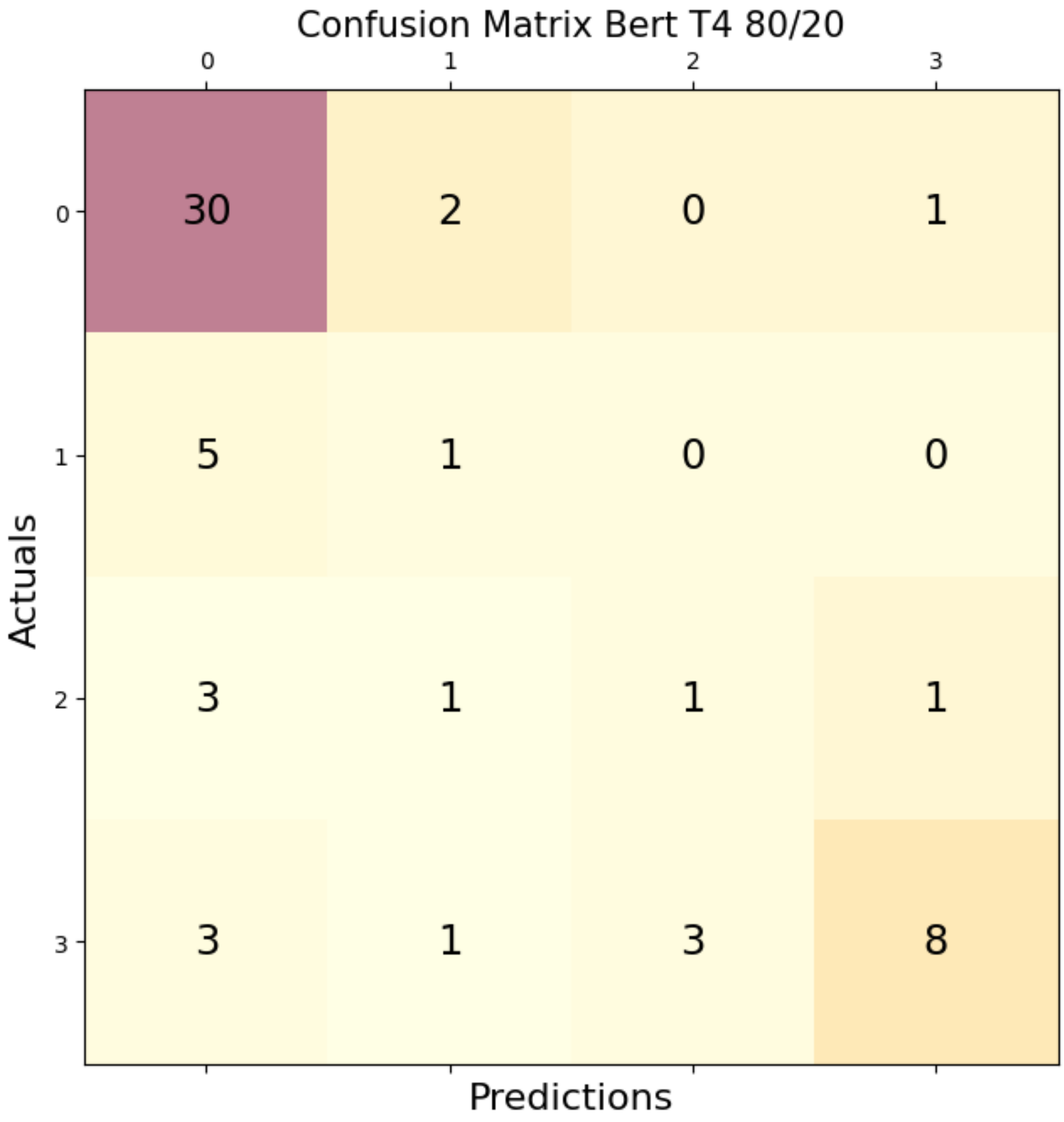

```
print(classification_report(y_test_t4_20, list(pred_t4_20), labels=[0,1,2,3
```

```
              precision    recall  f1-score   support

           0       0.73      0.91      0.81        33
           1       0.20      0.17      0.18         6
           2       0.25      0.17      0.20         6
           3       0.80      0.53      0.64        15

    accuracy                           0.67        60
   macro avg       0.50      0.44      0.46        60
weighted avg       0.65      0.67      0.64        60
```

```
#import torch

# Optional model (student_4class) configuration
model_args = ClassificationArgs(sliding_window=True)
model_args.num_train_epochs = 10
model_args.max_seq_length = 512
model_args.best_model_dir = '/content/drive/MyDrive/HE/outputt2_20/bestModel
model_args.output_dir = '/content/drive/MyDrive/HE/outputt2_20'

#cuda_available = torch.cuda.is_available()
# Create a ClassificationModel
model_t2_20 = ClassificationModel(
    'bert',
    # 可換成訓練過的model
    'bert-base-multilingual-uncased',
    num_labels=2,
    use_cuda=True,
    args=model_args
)
```

顯示隱藏的輸出內容

```
model_t2_20.train_model(train_df_t2_20)
```

顯示隱藏的輸出內容

```
pred_t2_20, outputs_t2_20 = model_t2_20.predict(x_test_t_20)
```

顯示隱藏的輸出內容

```
1 create_con_mat(pred_t2_20, y_test_t2_20, [0,1], "Confusion Matrix Bert T2 8
```

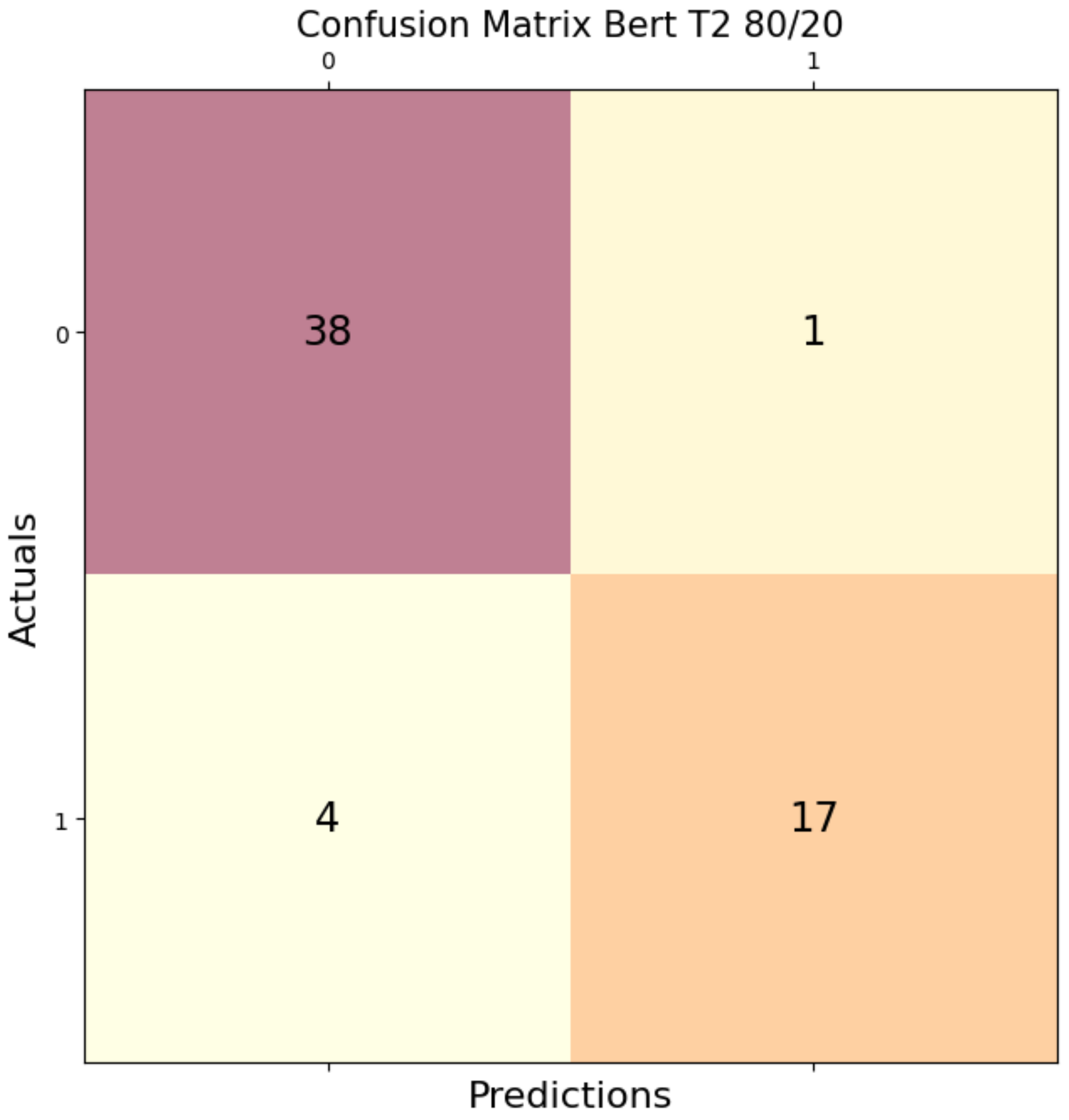

```python
print(classification_report(y_test_t2_20, list(pred_t2_20), labels=[0,1]))
```

```
              precision    recall  f1-score   support

           0       0.90      0.97      0.94        39
           1       0.94      0.81      0.87        21

    accuracy                           0.92        60
   macro avg       0.92      0.89      0.91        60
weighted avg       0.92      0.92      0.92        60
```

Teacher 75/25

```
# Preparing data for 75/25 teachers' records
train_t4_25 = []
train_t2_25 = []
test_t4_25 = []
test_t2_25 = []
x_test_t_25 = []
y_test_t4_25 = []
y_test_t2_25 = []


with open('/content/drive/MyDrive/HE/train_t_25.csv', newline='', encoding=
    rows = csv.reader(f)
    for row in rows:
        t4 = []
        t4.append(str(row[0]))
        t4.append(int(row[1]))
        train_t4_25.append(t4)
        t2 = []
        t2.append(str(row[0]))
        t2.append(int(row[2]))
        train_t2_25.append(t2)

with open('/content/drive/MyDrive/HE/test_t_25.csv', newline='', encoding="
    rows = csv.reader(f)
    for row in rows:
        t4 = []
        t4.append(str(row[0]))
        t4.append(int(row[1]))
        test_t4_25.append(t4)
        x_test_t_25.append(t4[0])
        y_test_t4_25.append(t4[1])
        t2 = []
        t2.append(str(row[0]))
        t2.append(int(row[2]))
        test_t2_25.append(t2)
        y_test_t2_25.append(t2[1])


train_df_t4_25 = pd.DataFrame(train_t4_25)
train_df_t4_25.columns = ["text", "labels"]

train_df_t2_25 = pd.DataFrame(train_t2_25)
train_df_t2_25.columns = ["text", "labels"]

eval_df_t4_25 = pd.DataFrame(test_t4_25)
eval_df_t4_25.columns = ["text", "labels"]

eval_df_t2_25 = pd.DataFrame(test_t2_25)
eval_df_t2_25.columns = ["text", "labels"]
```

```
#import torch

# Optional model (student_4class) configuration
model_args = ClassificationArgs(sliding_window=True)
model_args.num_train_epochs = 10
model_args.max_seq_length = 512
model_args.best_model_dir = '/content/drive/MyDrive/HE/outputt4_25/bestMode
model_args.output_dir = '/content/drive/MyDrive/HE/outputt4_25'

#cuda_available = torch.cuda.is_available()
# Create a ClassificationModel
model_t4_25 = ClassificationModel(
    'bert',
    # 可換成訓練過的model
    'bert-base-multilingual-uncased',
    num_labels=4,
    use_cuda=True,
    args=model_args
)
```

顯示隱藏的輸出內容

```
model_t4_25.train_model(train_df_t4_25)
```

顯示隱藏的輸出內容

```
pred_t4_25, outputs_t4_25 = model_t4_25.predict(x_test_t_25)
```

顯示隱藏的輸出內容

```
1 create_con_mat(pred_t4_25, y_test_t4_25, [0,1,2,3], "Confusion Matrix Bert
```

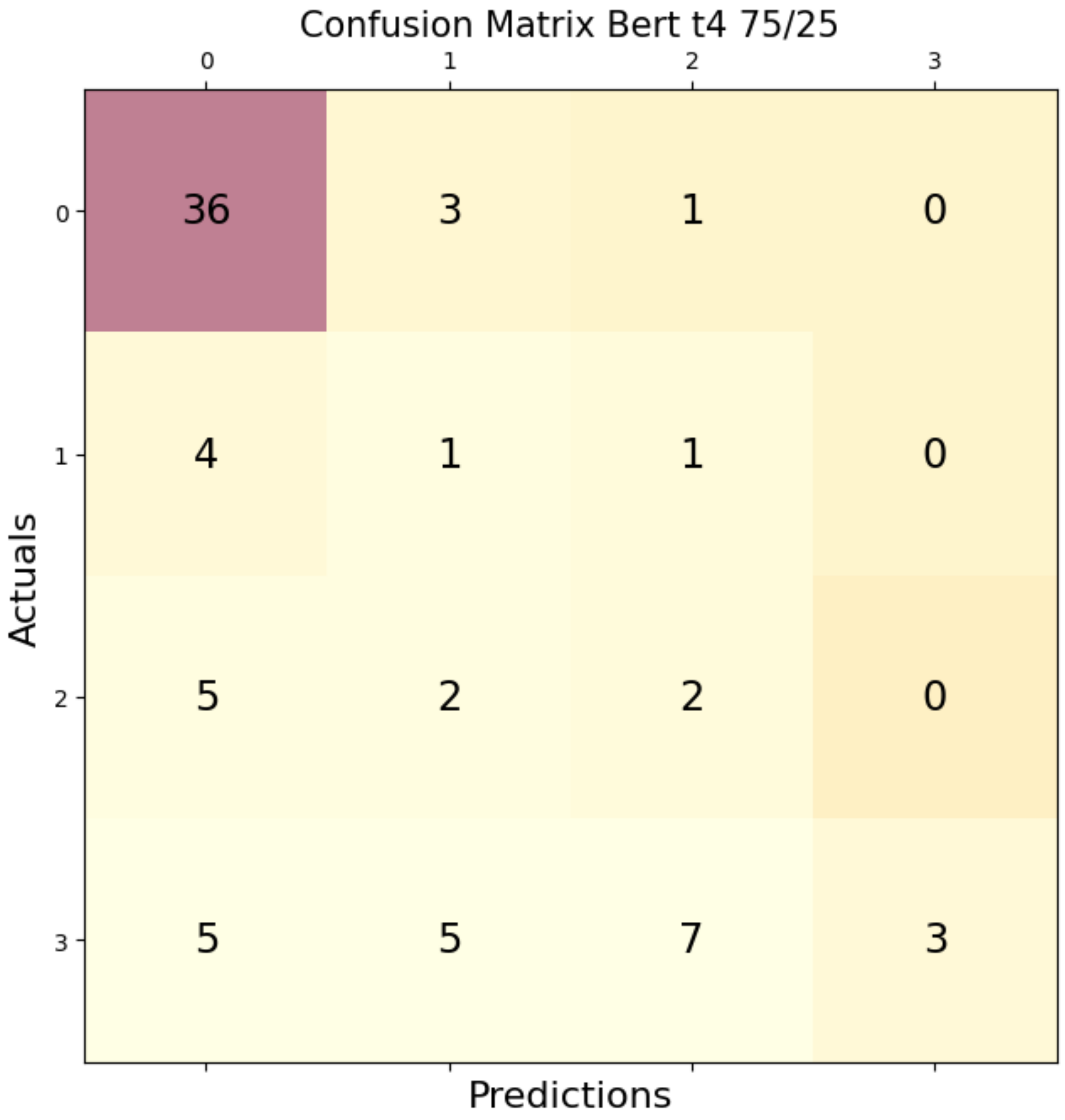

```
print(classification_report(y_test_t4_25, list(pred_t4_25), labels=[0,1,2,3
```

```
              precision    recall  f1-score   support

           0       0.72      0.90      0.80        40
           1       0.09      0.17      0.12         6
           2       0.18      0.22      0.20         9
           3       1.00      0.15      0.26        20

    accuracy                           0.56        75
   macro avg       0.50      0.36      0.34        75
weighted avg       0.68      0.56      0.53        75
```

```

# Optional model (student_4class) configuration
model_args = ClassificationArgs(sliding_window=True)
model_args.num_train_epochs = 10
model_args.max_seq_length = 512
model_args.best_model_dir = '/content/drive/MyDrive/HE/outputt2_25/bestModel
model_args.output_dir = '/content/drive/MyDrive/HE/outputt2_25'

#cuda_available = torch.cuda.is_available()
# Create a ClassificationModel
model_t2_25 = ClassificationModel(
    'bert',
    # 可換成訓練過的model
    'bert-base-multilingual-uncased',
    num_labels=2,
    use_cuda=True,
    args=model_args
)
```

顯示隱藏的輸出內容

```
model_t2_25.train_model(train_df_t2_25)
```

顯示隱藏的輸出內容

```
pred_t2_25, outputs_t2_25 = model_t2_25.predict(x_test_t_25)
```

顯示隱藏的輸出內容

```
1 create_con_mat(pred_t2_25, y_test_t2_25, [0,1], "Confusion Matrix Bert t2 7
```

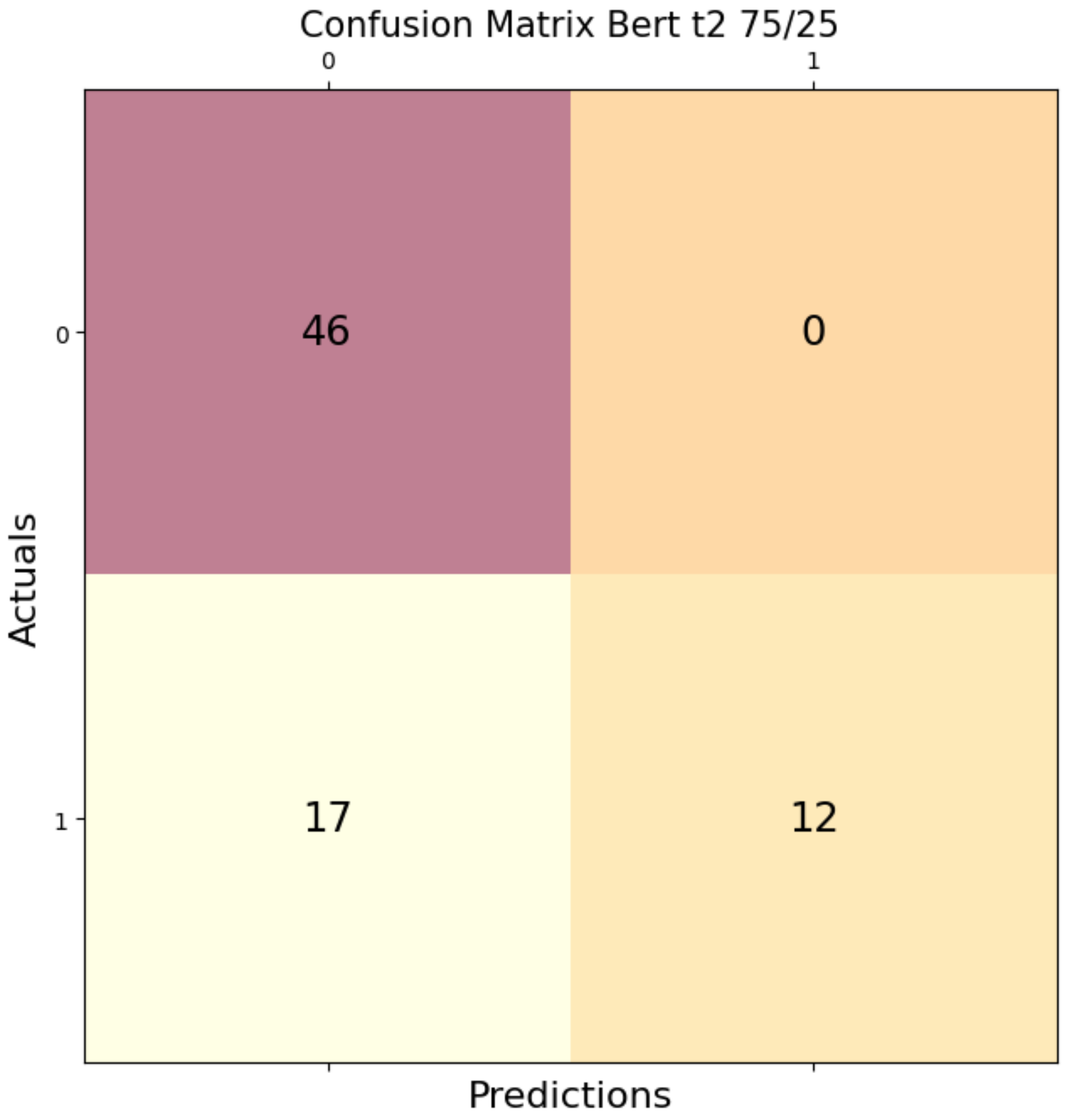

```python
1 print(classification_report(y_test_t2_25, list(pred_t2_25), labels=[0,1]))
```

```
              precision    recall  f1-score   support

           0       0.73      1.00      0.84        46
           1       1.00      0.41      0.59        29

    accuracy                           0.77        75
   macro avg       0.87      0.71      0.71        75
weighted avg       0.83      0.77      0.74        75
```
