# Supplementary material for: Automated Evaluation of Reflection and Feedback Quality in Workplace-Based Assessments by Using Natural Language Processing: Cross-Sectional Competency-Based Medical Education Study
Source: JMIR Med Educ. 2025 Oct 22;11:e81718. doi: 10.2196/81718 (PMC12590046; doi:10.2196/81718)
Supplement: Multimedia Appendix 4 [file mededu_v11i1e81718_app4.pdf]

Multimedia Appendix 4.

Table S2. Sample outputs from the BERT model for classifying narrative quality in resident reflections and faculty feedback. (E: effective; M: moderate; IE: ineffective; IR: irrelevant; H: high-quality; L: low-quality)

Part I: Resident Reflections

Workplaces	EPA	Clinical Diagnosis	Resident Reflections	Expert	BERT	Validation
Ward	EPA07 (Ear)	Right cholesteatoma, suspect with erosion of tegmen	膽脂瘤是一種由皮膚鱗狀角質化上皮所積聚而成的囊性結構，而不是真正的腫瘤，因為富含角質，外觀呈現珍珠樣的白色色澤，所以又它稱為「珍珠瘤」。之所以一定要開刀清除，是因為膽脂瘤所分泌的一些物質是細菌生長的溫床，而且其中所蘊含的酵素會破壞骨質壁，由於我們的中耳腔或乳突與中、後顱窩十分接近，一旦這層薄薄的骨質板被侵蝕，發炎的物質就會進入腦部造成續發性的腦膜炎、腦炎，嚴重者甚至轉變成腦膿瘍等，所以務必以手術根除膽脂瘤。	M (H)	E (H)	Misclassified in 4-level; Correct in 2-level
ER	EPA02 (FB)	Fish bone at posterior pharyngeal wall, removed	魚刺在後咽壁，一開始經口無法看到，用內視鏡看到後又無法順利夾出來，後來經口用力下壓舌頭後，發現可以看見魚刺，便經口夾出。	E (H)	E (H)	Correct in both 4-level and 2-level classification
OR	EPA01 (Airway)	Stridor	今天參與了 OOO 醫師的 Tracheostomy，希望慢慢熟練後，未來有機會可以進行操作學習	IE (L)	IE (L)	Correct in both 4-level and 2-level classification

Part I: Resident Reflections (continued)

Workplaces	EPA	Clinical Diagnosis	Resident Reflections	Expert	BERT	Validation
OR	EPA09 (Larynx)	Laryngeal papilloma	終於換我來照顧賢謝的老病人了，在術中看到小朋友的 papilloma 長的範圍比想像多很多，真的蠻可憐的。最後放了個 T 型管幫住支撐喉頭，這也是我第一次看到 T 型管如何放置，自認為自己見識太淺薄，還有很多東西還沒學。	E (H)	E (H)	Correct in both 4-level and 2-level classification
ER	EPA02 (FB)	Right EAC foreign body	Right EAC foreign body removal	IE (L)	M (H)	Misclassified in both 4-level and 2-level classification

Workplaces (OPD: outpatient department; OR: operation room; ER: emergency room; Ward; Consultation)

Part II: Faculty Feedback

Workplaces	EPA	Clinical Diagnosis	Faculty Feedback	Expert	BERT	Validation
OR	EPA08 (Sinonasal)	Left maxillary sinus recurrence cancer	內視鏡手術治療鼻竇炎進階至良性腫瘤，甚至惡性腫瘤，有賴於進一步學習 approach 的選擇，手術邊緣的辨識與 frozen 確認。	E (H)	E (H)	Correct in both 4-level and 2-level classification
ER	EPA02 (FB)	Fish bone at posterior pharyngeal wall, removed	異物哽塞需配合病人臨床症狀，若有明顯吞嚥困難且能明確說出哽塞時間，真的有異物的機會就上升。	E (H)	E (H)	Correct in both 4-level and 2-level classification

Part II: Faculty Feedback (continued)

Workplaces	EPA	Clinical Diagnosis	Faculty Feedback	Expert	BERT	Validation
Ward	EPA07 (Ear)	Middle ear effusion	技術純熟靈巧。	IE (L)	E (H)	Misclassified in both 4-level and 2-level classification
OR	EPA09 (Larynx)	Epiglottic tumor	年紀大的老人家 Laryngeal (supraglottic) tumor 並沒有影響到很大的呼吸或吞嚥及發聲功能，原則上就是以 minimal invasive endoscopic approach 的方式進行 tumor excision 為主，減少 co-morbidity，也縮短手術麻醉時間以降低麻醉風險，後續的 pathology report 值得追蹤看看到底是什麼東西?	E (H)	E (H)	Correct in both 4-level and 2-level classification
OR	EPA08 (Sinonasal)	Bilateral chronic sinusitis with subacute right periorbital abscess	工作認真負責，上進積極，未來良醫。	IR (L)	IE (L)	Misclassified in 4-level; Correct in 2-level classification

Workplaces (OPD: outpatient department; OR: operation room; ER: emergency room; Ward; Consultation)
