# Supplementary material for: Automated Evaluation of Reflection and Feedback Quality in Workplace-Based Assessments by Using Natural Language Processing: Cross-Sectional Competency-Based Medical Education Study
Source: JMIR Med Educ. 2025 Oct 22;11:e81718. doi: 10.2196/81718 (PMC12590046; doi:10.2196/81718)
Supplement: Multimedia Appendix 5 [file mededu_v11i1e81718_app5.pdf]

Multimedia Appendix 5

Table S3. Distribution of number (percentage) of 4-level and 2-level quality ratings for resident reflections and faculty feedback across pilot year (2021-2022), 2022-2023, 2023-2024, and 2024-2025

	**Resident Reflections**				**Faculty Feedback**			
	**Pilot Year**	**2022-2023**	**2023-2024**	**2024-2025**	**Pilot Year**	**2022-2023**	**2023-2024**	**2024-2025**
**4-Level**								
Effective	667 (46.9)	6,380 (64.2)	6,219 (58.7)	9,977 (82.2)	563 (39.6)	5,491 (55.2)	6,415 (60.5)	10,081 (83.0)
Moderate	352 (24.8)	3,138 (31.6)	4,111 (38.8)	2,018 (16.6)	104 (7.3)	1,220 (12.3)	1,150 (10.9)	889 (7.3)
Ineffective	243 (17.1)	302 (3.0)	229 (2.2)	30 (0.3)	112 (7.9)	964 (9.7)	1,007 (9.5)	505 (4.2)
Irrelevant	160 (11.3)	119 (1.2)	42 (0.4)	114 (0.9)	643 (45.2)	2,264 (22.8)	2,029 (19.1)	664 (5.5)
Total	1,422 (100)	9,939 (100)	10,601 (100)	12,139 (100)	1,422 (100)	9,939 (100)	10,601 (100)	12,139 (100)
**2-Level**								
High-quality	999 (70.3)	9463 (95.2)	10286 (97.0)	12,074 (99.5)	719 (50.6)	6655 (67.0)	7454 (70.3)	10,794 (88.9)
Low-quality	423 (29.7)	476 (4.8)	315 (3.0)	65 (0.5)	703 (49.4)	3284 (33.0)	3147 (29.7)	1,345 (11.1)
Total	1,422 (100)	9,939 (100)	10,601 (100)	12,139 (100)	1,422 (100)	9,939 (100)	10,601 (100)	12,139 (100)
