# Supplementary material for: Automated Evaluation of Reflection and Feedback Quality in Workplace-Based Assessments by Using Natural Language Processing: Cross-Sectional Competency-Based Medical Education Study
Source: JMIR Med Educ. 2025 Oct 22;11:e81718. doi: 10.2196/81718 (PMC12590046; doi:10.2196/81718)
Supplement: Multimedia Appendix 6 [file mededu_v11i1e81718_app6.pdf]

**Multimedia Appendix 5**

Detailed Process for evaluating resident reflections and faculty feedback quality using ChatGPT-4o

1. Role Definition

The first step was to specify ChatGPT's role.

- ChatGPT was instructed to act as a medical education expert.
- Its task was to evaluate Entrustable Professional Activity (EPA) narrative quality in resident reflections (or faculty feedback).
- This role ensured that the model approached the task with domain-specific expectations rather than general text analysis.

2. Rule Specification

The next step involved defining evaluation rules.

- The rubrics (shown in Table 1) were provided to ChatGPT.
- These rubrics serve as structured frameworks for assessing the quality and content of reflections (by residents) and feedback (by faculty members).
- By supplying the rubrics, the grading process was anchored in standardized and validated assessment criteria.

3. Application of Retrieval-Augmented Generation (RAG)

To improve grading consistency and alignment with human judgment, RAG methodology was applied.

- Multiple human-labeled training datasets were introduced.
    - ◆ These included both resident reflections and faculty feedback that had already been graded by expert evaluators.
- During evaluation, ChatGPT would:
    (1) Retrieve examples of similar human-graded texts from the dataset.
    (2) Combine the retrieved labels with the rules.
    (3) Auto-grade the new testing data based on the synthesis of rules and retrieved labels.
- This process provided ChatGPT with contextual grounding, making its judgments less

arbitrary.

4. Evaluation

After setup, ChatGPT was tasked to auto-grade the testing datasets, which were the same as those used by other evaluation models (e.g., BERT).

Resident Reflections Classification Results

- 4-level scale (Effective → Moderate → Ineffective → Irrelevant)
- 2-level scale (High vs. Low quality)

| | Accuracy | Recall | Precision | F1-score |
|---|---|---|---|---|
| 4-level | 42% | 42% | 42% | 37% |
| 2-level | 63% | 63% | 75% | 67% |

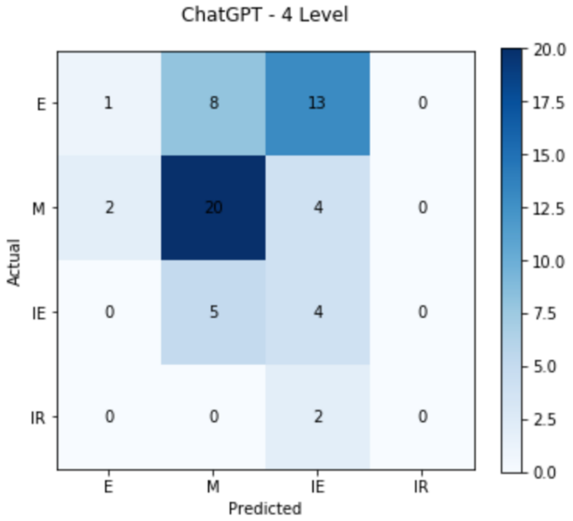


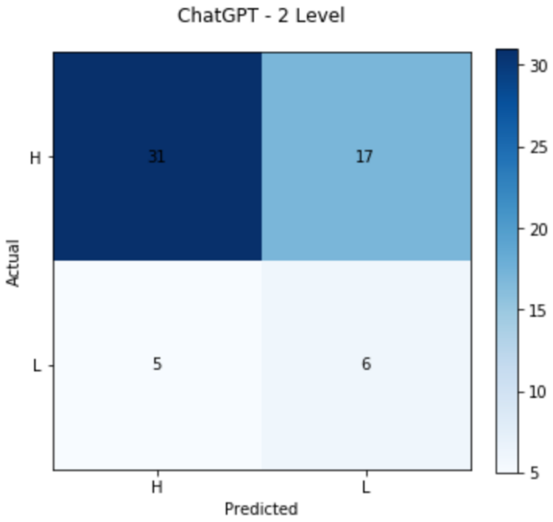


Faculty Feedback Results

- 4-level scale (Effective → Moderate → Ineffective → Irrelevant)
- 2-level scale (High vs. Low quality)

| | Accuracy | Recall | Precision | F1-score |
|---|---|---|---|---|
| 4-level | 12% | 12% | 38% | 13% |
| 2-level | 54% | 54% | 55% | 55% |

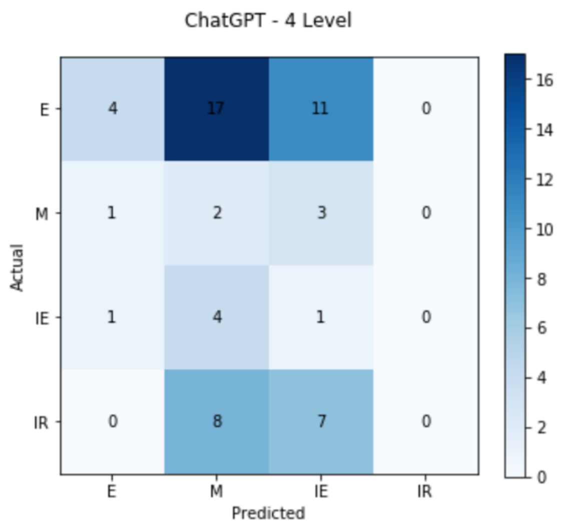


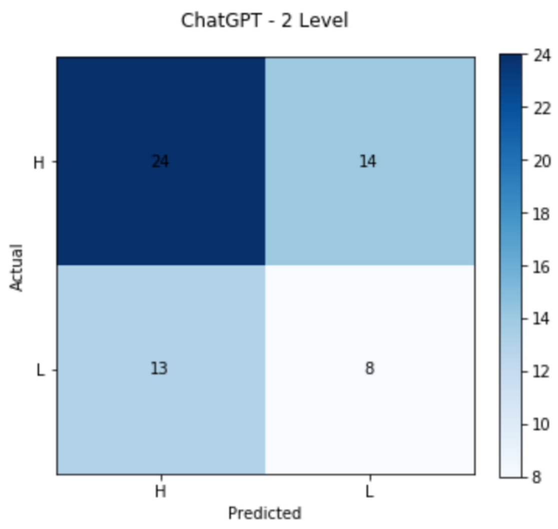


5. Conclusion

- Compared to BERT and other machine learning models, ChatGPT's performance was significantly inferior.
- A major issue observed: ChatGPT sometimes assigned different grades to identical content, showing lack of consistency and reliability.
- Therefore, the study concluded that the current version of ChatGPT is not suitable for this EPA assessment task.
- However, the findings suggested that future versions (e.g., ChatGPT-5) combined with refined prompt engineering strategies (such as prompt chaining) may offer improvements in accuracy and stability.
